# Lactate-driven ATP6V1B2 lactylation triggers asthmatic inflammation by linking lysosomal dysfunction to mitochondrial ROS-dependent pyroptosis

**DOI:** 10.1016/j.redox.2026.104059

**Published:** 2026-01-30

**Authors:** Qiaoyun Bai, Ningpo Ding, Rixin Feng, Fengxiang Shang, Zongqi Wang, Liangchang Li, Zhiguang Wang, Yihua Piao, Guangyu Jin, Yilan Song, Guanghai Yan

**Affiliations:** a Jilin Key Laboratory for Immune and Targeting Research on Common Allergic Diseases, Yanbian University, Yanji, 133002, PR China; b Department of Anatomy, Histology and Embryology, Yanbian University Medical College, Yanji, 133002, PR China; c Department of Respiratory Medicine, Affiliated Hospital of Yanbian University, Yanji, 133000, PR China; d Department of Critical Care Medicine, Affiliated Hospital of Yanbian University, Yanji, 133000, PR China; e Department of Radiology, Affiliated Hospital of Yanbian University, No.1327, Juzi Street, Yanji, Jilin Province, 133000, PR China; f Key Laboratory of Natural Medicines of Changbai Mountain, Ministry of Education, Yanbian University, Yanji, 133002, PR China

**Keywords:** Asthma, Lactylation, ATP6V1B2, Lysosomal dysfunction, Pyroptosis

## Abstract

Immunometabolic reprogramming is increasingly recognized as a driver of asthma pathogenesis, yet the molecular mechanisms linking lactate accumulation to airway inflammation via protein lactylation (Kla) remain elusive. In this study, we integrated a house dust mite (HDM)-induced asthma model with quantitative lactylomics to identify ATP6V1B2, a key V-ATPase subunit, as a core lactylation target. Combined molecular dynamics simulations and biochemical analyses revealed that intracellular l-lactate triggers lactylation at K108/K109. This modification restricts ATP6V1B2 conformational flexibility, leading to the disassembly of the V1–V0 complex and subsequent loss of proton pump activity. Crucially, the lactylation event was validated in primary human bronchial epithelial cells (HBEs), confirming that HDM and l-lactate stimulation induce ATP6V1B2 lactylation, thereby ensuring the clinical relevance of our findings. We demonstrate that this loss-of-function precipitates lysosomal alkalinization and membrane permeabilization (LMP). Crucially, LMP acts as a central node that bifurcates into two pathogenic cascades: it triggers a catastrophic mitochondrial ROS burst via Cathepsin B leakage. This oxidative burst functions as a pivotal redox signal that initiates a non-canonical Caspase-8/3/GSDME-dependent pyroptosis pathway, distinct from intrinsic apoptosis. In vivo, blocking ATP6V1B2 lactylation using an AAV-delivered lactylation-deficient (2 KR) mutant successfully severed this metabolic-inflammatory loop, significantly attenuating airway inflammation, Th2 cytokine release, and tissue pyroptosis. These findings characterize a novel "l-lactate–ATP6V1B2–GSDME" axis, establishing ATP6V1B2 lactylation as a critical metabolic switch connecting lysosomal damage to inflammatory cell death, thereby identifying a potential therapeutic target for metabolic dysregulation in chronic asthma with severe pathology.

## Highlights

• l-lactate triggers ATP6V1B2 lactylation at K108/K109 in asthma.
• ATP6V1B2 lactylation impairs V-ATPase assembly and induces LMP.
• Lysosomal damage drives mitochondrial ROS burst via Cathepsin B-tBid axis.
• Mitochondrial ROS signals GSDME-dependent pyroptosis via Caspase-8/3.
• Blocking ATP6V1B2 lactylation alleviates asthmatic airway inflammation.

## Graphical abstract

**Figure:**
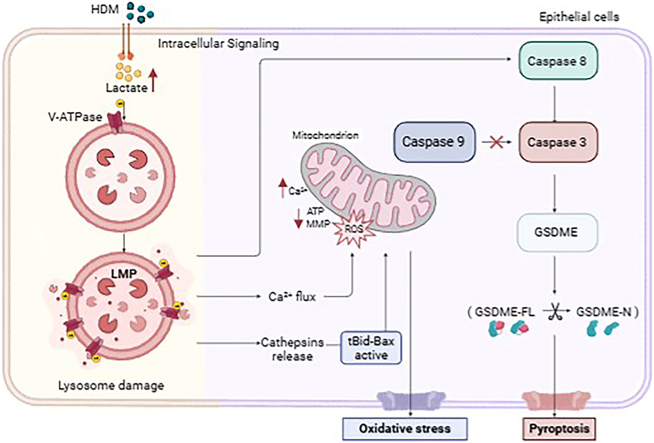

## 1 Introduction

Asthma is a heterogeneous disease characterized by airway hyperresponsiveness, airway remodeling, and chronic airway inflammation, affecting hundreds of millions of people globally. Its classical pathological mechanisms primarily involve eosinophilic infiltration and overexpression of Th2-type cytokines (e.g., IL-4, IL-5, IL-13). Although existing therapies, such as inhaled corticosteroids, effectively control symptoms in most patients, a subset of severe asthma patients exhibits refractory responses [1]. This suggests our understanding of the molecular mechanisms driving airway inflammation remains incomplete, necessitating the exploration of novel therapeutic targets Distinct from classical pathways.

Recent research in the field of immunometabolism revealed that metabolic reprogramming is not merely a consequence of immune activation but a core factor determining immune cell function [2]. In various inflammatory diseases (such as COVID-19), enhanced glycolytic pathways are closely related to inflammatory responses [3]; proinflammatory factors (such as IL-1β) can also induce glycolytic metabolic shifts [4]. Evidence of metabolic dysregulation in asthma is increasingly concrete: patients exhibit significantly elevated serum lactate levels [5], and airway epithelial cells show distinct glycolytic characteristics [6]. Lactate, long viewed as metabolic waste, has been redefined as a critical immunometabolite [[7], [8], [9]].

The discovery of lysine lactylation (Kla) in 2019 established a direct link between cellular metabolic status and protein function [10]. In asthma pathology, lactate dehydrogenase A (LDHA), which mediates lactate generation, is abnormally expressed [11], and inhibiting LDHA can curb inflammatory phenotypes [[12], [13], [14]], suggesting that lactate-driven protein modifications may play a key role in asthma. Although Kla has been confirmed to be involved in regulating Th17 differentiation [15]and fibrosis [16,17], its specific substrates and functions in asthma, a Th2-type inflammation, remain unclear [18].

Lysosomes, as the cellular degradation centers, rely on the acidic environment maintained by vacuolar H^+^-ATPase (V-ATPase) for their function. Disorders of lysosomal acidification are closely related to various diseases, including pancreatitis [19] and neurodegenerative diseases [20,21]. ATP6V1B2 is a key subunit of the V-ATPase complex, crucial for holoenzyme assembly and proton pump activity [22]. ATP6V1B2 dysfunction or gene mutation can lead to disrupted lysosomal homeostasis and cell death [23,24]; in the lung, its abnormal expression is associated with lung injury [25,26]. While ATP6V1B2 is known to be regulated by phosphorylation [22], whether lactylation directly regulates ATP6V1B2 function and thereby affects asthma pathology has not been reported.

Lysosomal dysfunction often induces pyroptosis, a highly proinflammatory programmed cell death [27,28]. However, whether there is a molecular causal link among disordered lactate metabolism, lysosomal damage, and pyroptosis in asthma remains unknown. This study combined quantitative lactylome analysis, molecular dynamics simulations, and AAV gene intervention techniques to systematically investigate the mechanism by which l-lactate induces lysosomal membrane permeabilization (LMP) and GSDME-mediated pyroptosis through ATP6V1B2 lactylation, aiming to provide a new theoretical basis for asthma treatment.

## 2 Materials and methods

### 2.1 Experimental animals and ethical statement

Female BALB/c mice aged 6–8 weeks and weighing 20–22 g were purchased from the Experimental Animal Center of Yanbian University. All mice were housed in independent ventilated cages (IVC) within a specific pathogen-free (SPF) grade barrier system. The housing environment was strictly controlled: temperature maintained at 22 ± 2 °C, relative humidity kept at 50 ± 10%, with a 12-h light/12-h dark cycle enforced (light period 07:00–19:00). Mice had ad libitum access to standard irradiated pellet feed and filtered sterile drinking water. Before experiments began, all animals underwent adaptive housing in the same environment for at least 7 days to minimize the impact of stress on experimental results. All animal experimental operations and protocol designs were rigorously reviewed and approved by the Ethics Committee of Yanbian University Medical College (Approval No.: YD20230710003; Approval Date: July 10, 2023) and strictly complied with the "Guide for the Care and Use of Laboratory Animals" and relevant animal welfare ethical principles.

### 2.2 Asthma model establishment, grouping, and drug intervention

After the adaptation period, mice were randomly assigned to four main experimental groups (n = 10 per group): Control group (Ctrl), house dust mite-induced asthma group (HDM), l-lactate intervention group (L-Lac), and Oxamate intervention group (OXA). During the sensitization phase (Day 0 and Day 7), mice in the HDM, L-Lac, and OXA groups received intraperitoneal (i.p.) injections of 100 μL of a freshly prepared suspension containing 50 μg HDM extract adsorbed in 2 mg aluminum hydroxide adjuvant; the sensitization solution for the L-Lac group additionally contained l-lactate (0.5 g/kg), and the OXA group additionally contained the lactate dehydrogenase inhibitor Oxamate (0.75 g/kg). The control group was injected only with 100 μL of sterile PBS containing an equivalent dose of aluminum hydroxide adjuvant.

During the challenge phase (Day 14 to Day 35), mice received intranasal instillations (i.n.) three times weekly under light isoflurane anesthesia, with the challenge solution consisting of 50 μg HDM dissolved in 50 μL sterile PBS. During this period, the L-Lac group received daily intraperitoneal injections of l-lactate (0.5 g/kg, pH adjusted to 6.8) 2 h before each HDM challenge, and the OXA group received intraperitoneal injections of Oxamate (0.75 g/kg). The control group received intranasal instillations and intraperitoneal injections of equivalent volumes of PBS.

Additionally, to validate the effects of direct airway exposure, we established a separate intranasal administration validation cohort (n = 10 per group). Following HDM sensitization as described above, mice in this group received intranasal instillations of l-lactate solution three times weekly during the challenge phase, in addition to the HDM challenge. Specifically, each mouse received a single dose of 1.25 mg l-lactate, prepared as 50 μL of a 25 mg/mL solution in PBS, with the pH strictly adjusted to 7.0 to prevent local tissue irritation.

### 2.3 AAV-mediated lung gene delivery

To verify the function of ATP6V1B2 lactylation in vivo, adeno-associated virus serotype 9 (AAV9) vectors were used. Wild-type ATP6V1B2 carrying a C-terminal Flag tag (AAV-WT), lactylation-deficient mutant (K108R/K109R, AAV-2KR), lactylation-mimetic mutant (K108Q/K109Q, AAV-2KQ), and negative control vector (AAV-NC) were constructed, packaged, and purified by GeneChem Co., Ltd. (Shanghai). Seven days before initial sensitization (Day −7), after mice were completely anesthetized with isoflurane and suspended vertically, 50 μL of the corresponding AAV viral suspension (titer: 1.5 × 10^12^ v.g./mL) was slowly instilled into the nostrils using a micropipette until the mice completely inhaled the droplets into the lungs. One week after viral transduction (Day 0), all AAV intervention group mice underwent HDM sensitization and challenge according to the complete protocol of the aforementioned "L-Lac group," combined with l-lactate injections throughout the process to construct an asthma model under a high lactate environment.

### 2.4 Sample collection and processing

Twenty-four hours after the last challenge, mice were euthanized by intraperitoneal injection of an overdose sodium pentobarbital (50 mg/kg). Whole blood was collected via cardiac puncture, allowed to clot at room temperature for 30 min, then centrifuged at 4 °C, 3000 rpm for 15 min to collect serum supernatant, which was aliquoted and stored at −80 °C. Subsequently, the trachea was exposed and cannulated, and the lungs were slowly lavaged 3 times with 0.8 mL ice-cold sterile PBS, with recovery rate controlled above 80%; the recovered fluid was centrifuged at 4 °C, 1500 rpm for 10 min, supernatant was used for cytokine ELISA detection (stored at −80 °C), and cell pellet was resuspended in PBS for cell counting and classification. Finally, lungs were harvested; left lung was immediately snap-frozen in liquid nitrogen and transferred to −80 °C freezer for protein and RNA extraction; right lung tissue was immersed in 4% paraformaldehyde (PFA) solution for fixation for 24–48 h for histopathological analysis.

### 2.5 Cell culture, drug treatment, and transfection

Primary Human Bronchial Epithelial Cells (HBEpiC) were purchased from ScienCell Research Laboratories (Cat# 3210, Carlsbad, CA, USA). Cells were cultured in specialized Bronchial Epithelial Cell Medium (BEpiCM, Cat# 3211, ScienCell) supplemented with Bronchial Epithelial Cell Growth Supplement (BEpiCGS) and antibiotics. To ensure proper cell attachment, culture vessels were strictly pre-coated with Poly-l-Lysine (Cat# 0413, ScienCell) according to the manufacturer's instructions. All validation experiments were performed using cells between passages 2 and 4 (P2–P4) to ensure physiological consistency.

The human bronchial epithelial cell line, BEAS-2B (RRID: CVCL_0168), was purchased from the Cell Resource Center of the Shanghai Institutes for Biological Sciences, Chinese Academy of Sciences (Shanghai, China). The cell line was confirmed to be mycoplasma-free. Cells were cultured in high-glucose DMEM medium containing 10% fetal bovine serum (FBS) (BaiDi Biotechnology Co., Ltd., Guangzhou, China), 100 μg/mL streptomycin, and 100 U/mL penicillin in a constant temperature incubator at 37 °C with 5% CO_2_ saturated humidity.

For experimental treatments, both primary HBEpiC and BEAS-2B cells were switched to their respective complete media and co-incubated with sodium l-lactate (10 mM, pH 6.8–7.0, Sigma-Aldrich) and/or HDM (10 μg/mL) for 24 h. Specifically, for the cell type specificity validation experiments, primary HBEpiC were treated with Control, HDM (10 μg/mL), L-Lac (10 mM), or HDM + L-Lac for 24 h before Western Blot and IP analysis. In mechanistic studies, cells were pre-incubated with the following specific inhibitors for 2 h before stimulation: Ferrostatin-1, Necrosulfonamide, Z-VAD-FMK (10 μM), Z-IETD-FMK (10 μM), Z-DEVD-FMK (10 μM), Bafilomycin A1 (100 nM), Chloroquine (10 μM), 3-Methyladenine (3-MA, 3 mM), Cathepsin B inhibitor CA-074 Methyl Ester (Cat# HY-100350, MCE), BID inhibitor BI-6C9 (Cat# HY-103661, MCE), and calcium chelator BAPTA-AM (10 μM, Cat# HY-100545, MCE).

Plasmid transfection was performed using Lipofectamine 3000 (Cat# L3000015, Thermo Scientific); plasmid DNA was mixed with P3000 reagent and then incubated with diluted Lipofectamine 3000 for 15 min to form complexes, which were added dropwise to the cells. Gene silencing was performed using Lipofectamine RNAiMAX (Thermo Scientific) to transfect siRNA at a final concentration of 50 nM. Targets included: Bid (Thermo, s1985), GSDME (MCE, HY-RS05858), GSDMD (RiboBio, siG000079702A-1–5), Caspase-3 (RiboBio, siB1208141220-1-5), Caspase-8 (Santa Cruz, sc-29930), Caspase-9 (Santa Cruz, sc-29931), and RIPK1 (RiboBio, siG000008737A-1–5). For V-ATPase functional loss-of-function controls, siRNA targeting ATP6V1B2 (RiboBio, siG00000526A-1–5) was used to compare with the 2KQ mutant in proton-pumping and lysosomal pH assays.

### 2.6 Plasmids and gene transfection

All expression plasmids used in this study were synthesized, constructed, and sequence-verified by HonorGene Co., Ltd. (Changsha, China). Specifically, the overexpression constructs included pcDNA3.1-PQBP1-Flag (wild-type) and its key lactylation mutants (K108R, K109R, K108Q, K109Q), with the empty vector pcDNA3.1-Flag serving as the negative control. Transient transfections of these plasmids were performed in BEAS-2B cells. When cells achieved 70–80% confluency, the respective plasmids were transfected using Lipofectamine 3000 Transfection Reagent (#L3000015; Thermo Scientific, Sunnyvale, CA, USA) strictly following the manufacturer's instructions. After transfection, cells were cultured for an additional 24 h to ensure adequate expression of the exogenous protein or effective knockdown of the target gene before proceeding to downstream experimental stimulations, such as treatment with l-lactic acid or HDM.

### 2.7 Subcellular fractionation

Nuclear/cytoplasmic separation used Nuclear and Cytoplasmic Protein Extraction Kit (Cat# P0027, Beyotime), strictly following instructions. Cell membranes were lysed by adding reagents A and B to release cytoplasmic proteins; after centrifugation, nuclear proteins were obtained by lysing the pellet with reagent C. To precisely detect Cathepsin B leakage, Mitochondria/Cytosol Fractionation Kit (Cat# ab65320, Abcam) was used. Briefly, 1–5 × 10^7^ cells were collected, washed with PBS, resuspended in 1X Cytosol Extraction Buffer (containing DTT and protease inhibitors), and incubated in ice bath for 10 min. After homogenization using a glass homogenizer, centrifugation was performed at 4 °C, 700×*g* for 10 min to remove nuclei and unlysed cells. Supernatant was transferred to a new centrifuge tube and centrifuged at high speed at 4 °C, 10,000×*g* for 30 min. The pellet at this time was mitochondrial and lysosomal fractions, and the supernatant was a pure cytosolic fraction, which was immediately used for Western Blot analysis.

### 2.8 Histopathology and immunofluorescence

Fixed lung tissues were dehydrated through graded ethanol, cleared in xylene, embedded in paraffin, and cut into 4 μm thick sections. Sections were deparaffinized in xylene and rehydrated through graded ethanol, then subjected to Hematoxylin and Eosin (H&E) staining (assessing inflammatory cell infiltration), PAS staining (Cat# G1281, Solarbio) (assessing goblet cells and mucus secretion), and Masson's trichrome staining (Cat# G1345, Solarbio) (assessing collagen deposition).

For cell immunofluorescence (IF), live cells were seeded in confocal-specific dishes. For mitochondrial or lysosomal labeling, MitoTracker Red CMXRos (100–200 nM, Cat# M7512, Thermo) or LysoTracker Red DND-99 (1 μM, Thermo) was directly added to the medium 30 min before the end of treatment, incubating at 37 °C in the dark.

For tissue immunofluorescence and cell-type specificity validation of AAV transduction, lung tissue frozen sections were fixed with 4% PFA for 15 min, washed 3 times with PBS, permeabilized with 0.1% Triton X-100 for 10 min, and blocked with 5% BSA at room temperature for 1 h. To identify the cellular localization of AAV-delivered mutants, sections were incubated with Anti-Flag (Cat# 14793S) and Anti-EpCAM antibody [323/A3] (Cat# ab 85987, Abcam) at 4 °C overnight. Other primary antibodies used included Anti-L-Lactyl Lysine (Cat# PTM-1401RM, PTMBIO), DRP1 (Cat# 8570S, CST), ATP6V1B2 (Cat# sc-166045, SCBT), ATP6V0C (Cat# A16350, Abclonal), LAMP1 (Cat# 15665, CST), and Cathepsin B (Cat# A0967, Abclonal). The next day, samples were incubated with AlexaFluor 488 (Cat# R37118, Life Technologies) or Cy3 conjugated (Cat# 97035, Abcam) secondary antibodies at room temperature in the dark for 1 h. Nuclei were counterstained with DAPI mounting medium. Images were acquired using Cytation 5 or a laser scanning confocal microscope.

### 2.9 BALF cell classification and flow cytometry

After resuspending BALF cell pellets, 50 μL was taken for smearing, air-dried, then stained using Diff-Quik staining set (Cat# G1541, Solarbio). At least 200 cells were counted under a light microscope to distinguish eosinophils, macrophages, etc., based on morphological characteristics. For flow cytometric phenotypic analysis, cells were resuspended in FACS buffer (PBS containing 1% BSA), incubated with Fc receptor blockers, then added with fluorescent antibodies: PE-Siglec-F (Cat# 552126, BD), APC-CD45.2 (Cat# 558702, BD), and PerCP-Cy5.5 CD11c (Cat# 45-0114-82, Invitrogen), incubated at 4 °C in the dark for 30 min. For intracellular cytokine detection, single-cell suspensions were prepared from mediastinal lymph nodes and spleens, first subjected to surface staining PE-CD4 (Cat# 11-0041-82, Invitrogen), then treated using the Fixation/Permeabilization kit, followed by intracellular staining APC–IFN–γ (Cat# 554413, BD) and PE-Cy7 IL-4 (Cat# 504117, Biolegend). Data were acquired using a CytoFLEX flow cytometer and analyzed with CytExpert 2.4 software.

### 2.10 Cell viability, toxicity, and enzyme activity assays

For cell viability detection, cells were seeded in 96-well plates (6000 cells/well). After treatment, 10 μL CCK-8 solution (Cat# K1018, ApexBio) was added per well, incubated at 37 °C for 2 h, and absorbance at 450 nm was measured using a microplate reader. Cell death was detected by collecting floating cells in culture supernatant and cells, washing with PBS, resuspending in 1X Binding Buffer, adding 5 μL PE-Annexin V and 5 μL 7-AAD (Beckman Coulter), incubating at room temperature in the dark for 15 min, then immediately loading for detection. Cell cytotoxicity was determined by the LDH Cytotoxicity Assay Kit (Cat# BC0685, Solarbio); cell culture supernatant was centrifuged to remove debris, reaction solution was added, and absorbance at 450 nm was measured. Release rate (%) = [(Sample well – Spontaneous well)/(Maximum release well – Spontaneous well)] × 100%. LDH enzyme activity in lung tissue homogenate supernatant or serum samples was detected using Lactate Dehydrogenase Activity Assay Kit (Cat# MAK066, Sigma-Aldrich); samples were mixed with Master Reaction Mix, absorbance at 450 nm was measured every 5 min at 37 °C, and enzyme activity (milliunits/mL) was quantified by calculating NADH generation rate.

### 2.11 Metabolic flux and mitochondrial function analysis

BEAS-2B cells were seeded in Seahorse XF96 cell culture microplates and changed to unbuffered assay medium 1 h before the experiment. Oxygen consumption rate (OCR) measurement used Mito Stress Test Kit (#103015–100), final concentrations: Oligomycin (1.5 μM), FCCP (0.5 μM), Rotenone/Antimycin A (0.5 μM). Mitochondrial membrane potential was evaluated by incubating the JC-1 probe (5 μM, Cat# C2006, Beyotime) for 20 min and detecting the red/green fluorescence ratio. mPTP opening was detected using Calcein-AM/CoCl_2_ kit (Cat# C2009, Beyotime). Mitochondrial calcium was detected by co-staining Fluo-4 AM (1 μM) with MitoTracker Red, and colocalization analysis showed mitochondrial calcium levels. Intracellular ATP content was determined after lysing cells using the Luciferase ATP Assay Kit (Cat# S0026, Beyotime), measuring RLU values via a chemiluminescence instrument, and normalizing to protein concentration. V-ATPase hydrolysis activity detection involved extracting total cellular protein, removing endogenous phosphate interference, using ATPase Assay Kit (Cat# ab234055, Abcam) to react in buffer containing ATP substrate, and measuring released inorganic phosphate by colorimetry. To evaluate V-ATPase-dependent proton-pumping activity and lysosomal acidification, cells were incubated with LysoTracker Red DND-99 (1 μM, Thermo) for 30 min at 37 °C in the dark. Representative images were captured using a fluorescence microscope, and the mean fluorescence intensity (MFI) of the lysosomal compartments was quantified using ImageJ software. For refined functional validation, ATP6V1B2 knockdown (siRNA) was included as a positive control for V-ATPase dysfunction to compare with the effects of 2KQ and 2 KR mutants.

### 2.12 ROS detection and transmission electron microscopy

For intracellular total ROS and mitochondrial ROS detection, cells were incubated with DCFH-DA (10 μM, Cat# S0033S, Beyotime) or MitoSOX Red (5 μM, Cat# M36008, Thermo), respectively in serum-free medium at 37 °C for 20 min, washed 3 times with PBS, and then detected using flow cytometry or fluorescence microscope. For transmission electron microscopy (TEM) sample preparation, collected cells were rapidly fixed in 2.5% glutaraldehyde (prepared in 0.1 M phosphate buffer) for 2 h, followed by post-fixation in 1% osmium tetroxide for 1 h. Samples were dehydrated through graded acetone, embedded in epoxy resin, and cut into 70 nm ultrathin sections. Sections were double-stained with uranyl acetate and lead citrate, then observed and photographed using a Hitachi HT7700 transmission electron microscope.

### 2.13 ELISA and intracellular lactate measurement

Mouse serum total IgE and HDM-specific IgE (R&D Systems), as well as IL-4 (E-EL-M0043), IL-5 (E-EL-M0722), IL-13 (E-EL-M0727), HMGB1 (E-EL-M0676/E-EL-H1554), IL-1β (E-EL-H0149), and IL-18 (E-EL-H0253) in BALF or cell supernatants were all detected using ELISA kits from Elabscience. Experiments were strictly conducted according to instructions: standards and samples added, incubated then washed, biotinylated antibody and HRP conjugate added, developed, and absorbance measured at 450 nm. Intracellular lactate concentration was measured using the l-Lactate Assay Kit (Cat# BC2235, Solarbio); samples were deproteinized, then reacted with enzyme working solution, and absorbance was measured at 570 nm.

### 2.14 Immunoprecipitation, immunoblotting, and lactylome

For immunoprecipitation (IP), cell lysates (RIPA buffer containing protease/phosphatase/deacetylase inhibitors) were incubated with 2 μg anti-ATP6V1B2 antibody (Cat# ab73404, Abcam) or isotype control IgG at 4 °C with rotation overnight. The next day, 20 μL Protein A/G magnetic beads (Cat# HY-K0202, MCE) were added and incubated for 2 h; beads were washed, then eluted by boiling. To assess the impact of pathological stimuli on endogenous complex stability, cells were treated with HDM (10 μg/mL) and/or l-lactate (10 mM). Endogenous V1 subunits were immunoprecipitated using anti-ATP6V1B2, and the amount of co-precipitated V0 subunits was determined by immunoblotting with anti-ATP6V0A1 (13828-1-AP). Co-IP used mild lysis buffer (20 mM Tris-HCl pH 7.5, 150 mM NaCl, 1% NP-40, and 10% glycerol) to preserve the integrity of the V1–V0 complex for cells transfected with Flag plasmids, supernatant was incubated with Anti-Flag M2 Affinity Gel (Cat# A2220, Sigma) at 4 °C overnight. For Western Blot analysis, protein samples were separated by SDS-PAGE electrophoresis, transferred to PVDF membranes, blocked, then incubated with primary antibodies at 4 °C overnight. Primary antibodies included: L-Lactyl Lysine (Cat# PTM-1401RM), TRPML1 (Cat# ab272608), CAMKK2 (Cat# ab96531), GSDME (Cat# ab215191), GSDMD (Cat# ab219800), GSDMC (Cat# ab225635), Caspase-8 (Cat# ab308013), Caspase-3 (Cat# ab32351), Caspase-9 (Cat# ab32539), RIPK1 (Cat# ab276121), LC3B (Cat# ab192890), p62 (Cat# ab207305), ATP6V1B2 (Cat# ab73404), ATP6V0A1 (Cat# 13828-1-AP), Bid (Cat# 2002), XIAP (Cat# 14334), Flag (Cat# 14793S), β-actin (Cat# 3700s), Bcl-2 (Cat# CY5032), BAX (Cat# CY5059), and Cytochrome C (Cat# MA5-11674),DRP1 (Cat# 8570S), DRP1 (p)(ser616) (Cat# 3455S). Secondary antibodies, Goat Anti-Rabbit IgG H&L (#ab6721, Abcam) and Goat Anti-Mouse IgG H&L (#ab6789, Abcam), were incubated for 2 h at room temperature. Chemiluminescent signals were captured, and band intensities were quantified using Quantity One software (BioRad, Hercules, CA, USA). Densitometric measurements were performed with local background subtraction and confirmed to be within the linear detection range by analyzing serial dilutions of control samples. (BioRad, Hercules, CA, USA). The densitometric intensity of each target protein was normalized to the corresponding β-actin loading control from the same blot.

### 2.15 Quantitative lactylomics methods for BEAS-2B cells

The core objective of this quantitative proteomics approach was to specifically investigate how the elevated metabolic product, l-Lactate, drives protein lactylation. To ensure an unbiased and specific identification of direct lactylation targets without interference from other inflammatory or enzymatic components found in crude allergens like HDM, we limited the initial screening comparison to only the l-lactate-stimulated group (10 mM, 24 h) versus the control group. This strategy aimed to confirm that increased lactate levels are sufficient to directly induce the observed modifications and to establish a robust pool of potential targets for subsequent disease-specific validation experiments.

The core objective of this study was to employ quantitative proteomics to analyze protein lactylation modifications and their functional consequences in human bronchial epithelial cells (BEAS-2B) after l-lactate stimulation (10 mM, 24 h). Following cell culture and stimulation, samples from 3 biological replicates per group (n = 3) were first lysed, and total protein concentration was measured using a BCA assay kit. Subsequently, equal amounts of protein were precipitated with TCA and washed with cold acetone. The precipitates were digested overnight using trypsin (1:50 enzyme-to-protein ratio), and the resulting peptides were reduced with DTT and alkylated with IAA.

The critical step involved Lactyl-Peptide Enrichment: Peptides were dissolved in IP buffer and incubated overnight at 4 degrees C with specific pan-lactylation antibody beads (provided by PTM Bio). Modified peptides were isolated via affinity enrichment and finally eluted and lyophilized using 0.1% trifluoroacetic acid.

The enriched peptides were separated using the NanoElute ultra-high-performance liquid chromatography system and injected into the timsTOF Pro mass spectrometer. Data acquisition and analysis were performed using the data-independent parallel accumulation serial fragmentation (dia-PASEF) mode. Mass spectrometry data were searched against the *Homo sapiens* database using Spectronaut (v.17.0) software, with the false discovery rate (FDR) set to less than 1% for protein, peptide, and PSM levels. Quantitative analysis was performed using relative intensity values, and differential modification sites were filtered using a T-test (P value < 0.05, Fold Change [FC] > 1.5). Subsequent bioinformatics analysis showed that differentially modified proteins were significantly enriched in KEGG "Lysosome" and other related pathways.

### 2.16 Molecular dynamics simulation and bioinformatics

For structural and mechanistic investigation, AlphaFold3 was used to predict the ATP6V1B2 monomer and complex structures. The simulation systems for both the wild-type and the K108/K109 dual-site lactylation (KLA) mutant were built using CHARMM-GUI, employing the CHARMM36 m force field and the TIP3P water model. The system was solvated and neutralized by adding potassium (K+) and chloride (Cl-) ions to achieve a physiological salt concentration of 0.15 mol/L. All-atom molecular dynamics (MD) simulations were performed using GROMACS 2023.5. The system underwent energy minimization using the Steepest Descent algorithm (convergence criterion: maximum force <1000 kJ/(mol·nm) followed by 200 ps of NVT (300 K) and NPT (1 atm) equilibration. A 100 ns production run was conducted with a 2 fs integration time step. All simulations were performed in triplicate. Structural stability and convergence were evaluated by calculating the Root-Mean-Square Deviation (RMSD); all RMSD curves stabilized rapidly after initial equilibration, confirming sufficient conformational sampling. Root-Mean-Square Fluctuation (RMSF) was utilized as the primary quantitative flexibility metric to assess the impact of lactylation on the V1 and V0 subunit contact regions. Additionally, structural integrity was evaluated by calculating Radius of Gyration (Rg), Gibbs free energy landscape (FEL), and inter-subunit hydrogen bond numbers, which were used to quantify the lactylation-induced disruption of the V1–V0 assembly.

For bioinformatics analysis, we performed single-gene GSEA to analyze the correlation between ATP6V1B2 expression and KEGG/GO pathways (Fig. 8A). The public dataset used for this analysis was retrieved from the GEO database (GSE 43696). This dataset contains 108 samples of human bronchial epithelial cells. The samples were stratified into two groups based on the median expression level of ATP6V1B2: a high-expression group (n = 54) and a low-expression group (n = 54). The GSEA was then performed using standard procedures to identify gene sets significantly enriched in either the high or low ATP6V1B2 expression groups. Additionally, we utilized the same GSE43696 dataset to compare the mRNA expression levels of ATP6V1B2 and GSDME (DFNA5) across different patient groups (Control, Moderate, and Severe asthma) for clinical relevance analysis.

### 2.17 Measurement of cathepsin B activity

Intracellular Cathepsin B activity in BEAS-2B cells was quantified using the Cathepsin B Activity Assay Kit (ab65300, Abcam) according to the manufacturer's protocol. Briefly, cells were harvested after treatments, lysed in chilled Cell Lysis Buffer on ice, and centrifuged to obtain supernatants. Protein concentrations were determined for normalization. Lysates were incubated with Reaction Buffer and the specific substrate Ac-RR-AFC (200 μM) at 37 °C for 1–2 h in the dark. Fluorescence intensity was measured at Ex/Em = 400/505 nm using a microplate reader. Specificity was confirmed by including a negative control group treated with the Cathepsin B inhibitor.

### 2.18 Quantitative real-time PCR

Total RNA was extracted from BEAS-2B cells using the RNA Easy Fast Cell Total RNA Extraction Kit (#DP451; TIANGEN) following the manufacturer's protocol. Subsequently, 1 μg of total RNA was reverse transcribed into cDNA using the Fast One-Step Reverse Transcription Kit (#KR118; TIANGEN). Quantitative real-time PCR (RT-qPCR) was performed on an Azure Cielo 6 system (Azure Biosystems) using SuperReal PreMix Plus (SYBR Green) (#FP205; TIANGEN). The specific primer sequences were as follows: TRPML1 (Forward: 5′-TCCTGTTTGACGTGGTGGTC-3'; Reverse: 5′-TGAACCCCACAAACTCGTTCT-3'; product size: 105 bp) and GAPDH (Forward: 5′-TGCACCACCAACTGCTTAGC-3'; Reverse: 5′-GGCATGGACTGTGGTCATGAG-3′). Relative gene expression levels were calculated using the 2^-ΔΔCt method, with GAPDH serving as the internal control.

### 2.19 Statistical analysis

All experimental data were derived from at least three independent replicates (n ≥ 3 for in vitro experiments and n ≥ 10 for in vivo experiments), and results are expressed as Mean ± standard error of the mean (SEM). For normally distributed data comparisons between groups: unpaired two-tailed Student's t-test was used for two groups; one-way ANOVA followed by Tukey's multiple comparison test was used for multiple groups. Non-parametric tests were used for non-normally distributed data. Omics data P values were corrected for multiple hypothesis testing using the Benjamini-Hochberg (BH) method. All statistical analyses and plotting were completed using GraphPad Prism 10 and R software (v4.2.0). P < 0.05 was considered statistically significant.

## 3 Results

### 3.1 l-lactate metabolism exacerbates HDM-induced airway inflammation by driving Th2 immune responses

**Fig. 1:**
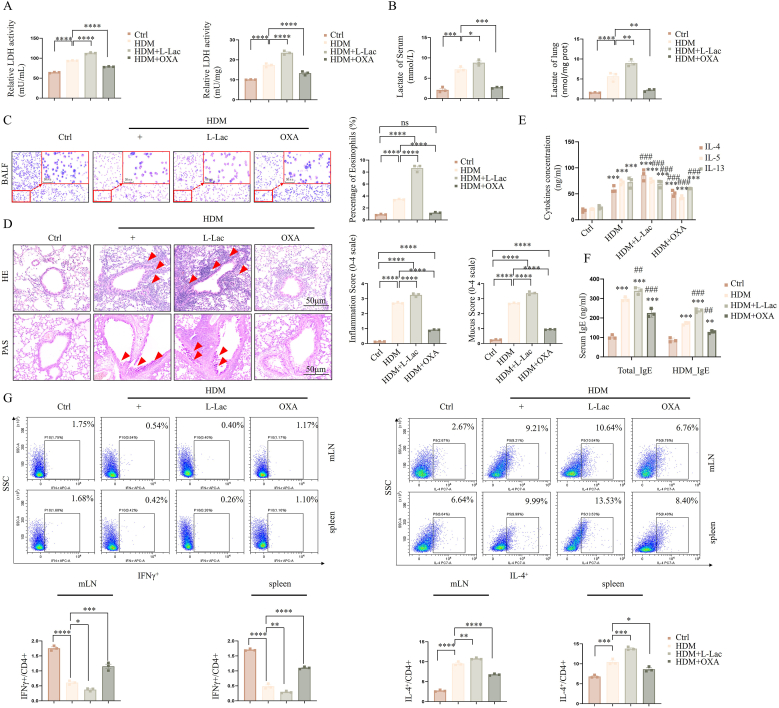
**Impact of****l****-lactate metabolism on HDM-induced murine airway inflammation and Th2 immune response.** (A) LDH enzyme activity detection in serum (left) and lung tissue homogenates (right) of mice in each group. (B) Quantitative analysis of lactate concentrations in serum and lung tissue. (C) Diff-Quik staining images of BALF cell smears (left, red box indicates magnified field) and statistics of eosinophil proportions (right). (D) H&E staining (top, showing inflammatory infiltration) and PAS staining (bottom, showing goblet cells and mucus) of lung tissue sections. Red arrows indicate lesion areas. Right side shows pathological scores. (E) ELISA detection of IL-4, IL-5, and IL-13 concentrations in BALF. (F) ELISA detection of serum total IgE and HDM-specific IgE levels. (G) Flow cytometric analysis of Th1 (IFN-γ^+^CD4^+^) and Th2 (IL-4^+^CD4^+^) cell subsets in mediastinal lymph nodes (mLN) and spleen. Data are expressed as Mean ± SEM. n = 10 per group. ∗P < 0.05, ∗∗P < 0.01, ∗∗∗P < 0.001, ∗∗∗∗P < 0.0001; ns, not significant (One-way ANOVA).

Using an HDM-induced mouse asthma model, this study systematically elucidated the core role of l-lactate (L-Lac) metabolism in driving airway inflammation. We found significant glycolytic reprogramming in the asthma model, characterized by markedly enhanced lactate dehydrogenase (LDH) enzymatic activity in lung tissue and serum (Fig. 1A), leading to elevated l-lactate levels (Fig. 1B). Functional intervention results showed that administration of exogenous L-Lac significantly aggravated asthma pathological features, including a sharp increase in eosinophil infiltration in BALF (Fig. 1C) and intensified airway inflammation and mucus hypersecretion (Fig. 1D). Immunologically, L-Lac further boosted levels of key Th2 cytokines (IL-4, IL-5, IL-13) in BALF (Fig. 1E) and led to elevated serum total IgE and specific IgE levels (Fig. 1F). Mechanistic investigation confirmed this process was directly related to Th2 cell expansion and a significant shift of the Th1/Th2 balance towards Th2 (Fig. 1G). Crucially, intervention with the LDH inhibitor OXA not only successfully inhibited HDM-induced LDH activity and the subsequent generation of endogenous l-lactate but also exhibited potent inhibitory effects on all pathological and inflammatory indices, demonstrating that endogenous l-lactate metabolism is a key upstream proinflammatory signal driving HDM-induced asthmatic inflammation and Th2 immune responses.

To rule out potential biases associated with the intraperitoneal (i.p.) administration route and further validate our findings, we performed orthogonal experiments using intranasal (i.n.) l-lactate administration to mimic local metabolic accumulation. As shown in Supplementary Fig. S1, the results from the i.n. group were highly consistent with those of the i.p. group, confirming that direct local airway delivery of l-lactate similarly exacerbates HDM-induced inflammation. This reproducibility was evident across all metabolic, immunological, and molecular indices. Specifically, i.n. Administration significantly elevated LDH activity (Fig. S1A) and l-lactate levels (Fig. S1B) in both lung tissue and serum. Furthermore, pathological phenotypes were intensified, characterized by increased inflammation scores, mucus hypersecretion (Fig. S1C), robust secretion of Th2 cytokines (IL-4, IL-5, IL-13) (Fig. S1D), and elevated serum IgE levels (Fig. S1E). Crucially, we confirmed that local l-lactate exposure was sufficient to induce significant ATP6V1B2 lactylation in lung tissue (Fig. S1F and 1G). These robust data provide compelling evidence that airway l-lactate accumulation is a decisive determinant of ATP6V1B2 lactylation and asthma pathology, independent of the administration route.

### 3.2 Elevated pan-lactylation levels in asthma and identification of the key lysosomal target ATP6V1B2

**Fig. 2:**
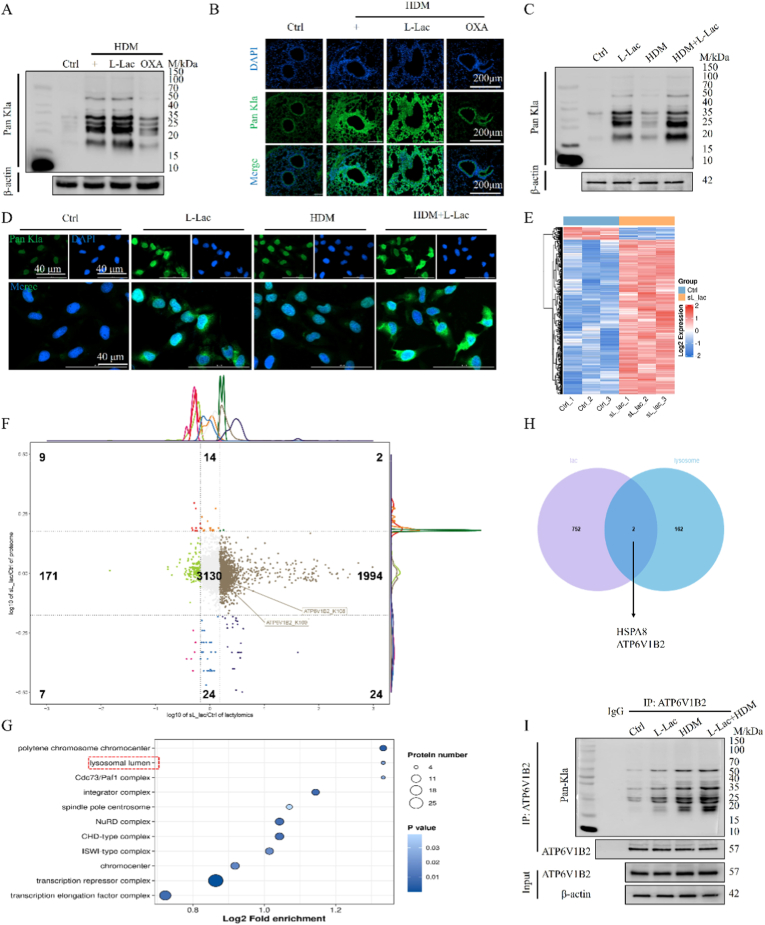
**Detection of pan-lactylation levels in asthma model and screening of target protein ATP6V1B2.** (A) Immunoblot analysis of pan-lactylation (Pan-Kla) levels in lung tissue lysates. (B) Immunofluorescence staining of Pan-Kla (green) in lung tissue sections. Nuclei stained with DAPI (blue). Scale bar: 200 μm. (C) Immunoblot detection of Pan-Kla levels in BEAS-2B cells under different treatment conditions (Ctrl, L-Lac, HDM, HDM + L-Lac). (D) Immunofluorescence staining of Pan-Kla (green) in BEAS-2B cells. Scale bar: 40 μm. (E) Heatmap of quantitative lactylome based on 4D-FastDIA (L_Lac vs Ctrl). (F) Nine-quadrant plot of omics data, labeling K108 and K109 sites of ATP6V1B2. (G) KEGG cellular component enrichment analysis of differentially lactylated proteins. (H) Venn diagram intersection analysis of highly methylated proteins from omics with GeneCards lysosome-related genes. (I) Immunoprecipitation (IP) of endogenous ATP6V1B2 in BEAS-2B cells and immunoblot analysis of lactylation levels (Pan-Kla).

Based on the aforementioned findings of metabolic abnormalities in lactate, we hypothesized that protein lactylation (Kla) is a key link in asthma pathology. Western Blot (WB) and immunofluorescence (IF) analysis confirmed that pan-lactylation (Pan-Kla) levels were significantly upregulated in the lung tissues of HDM-induced asthma mice (Fig. 2A and B). Exogenous L-Lac intervention further exacerbated this phenomenon, whereas blocking endogenous lactate production with the LDH inhibitor OXA significantly attenuated Kla levels, indicating that high lactylation is directly controlled by the lactate metabolic state. In vitro experiments yielded consistent results: both pathological concentrations of L-Lac or HDM stimulation induced significant elevation and nuclear-cytoplasmic diffuse distribution of Pan-Kla in BEAS-2B cells (Fig. 2C and D).

To identify key downstream molecules mediating lactate effects, we performed 4D-FastDIA quantitative lactylome analysis on L-Lac-treated BEAS-2B cells. Results showed significant upregulation of lactylation on a global scale (Fig. 2E and F). Subcellular localization analysis showed differential modified proteins were widely distributed in the nucleus, cytoplasm, and mitochondria (Supplementary Fig. S2A). However, KEGG enrichment analysis revealed that differentially modified proteins were significantly enriched in the "Lysosomal lumen" pathway (Fig. 2G), suggesting lactylation might specifically regulate lysosomal function. By intersecting differential modified proteins with lysosome-related genes in the GeneCards database (Fig. 2H), we screened two candidate proteins: HSPA8 and ATP6V1B2.

We rigorously discriminated between candidate molecules based on the dual criteria of "modification abundance change" and "biological function". Although HSPA8 was included, its K601 site lactylation showed a downward trend, and it primarily functions as a molecular chaperone, having a weaker specific association with lysosomal lumen function. In contrast, ATP6V1B2 showed high consistency: lactylation levels at its K108 and K109 sites were significantly upregulated (Ratio >1.60) (Supplementary Fig. S2B), and as a key subunit of V-ATPase directly responsible for lysosomal acidification, it closely aligned with the characteristics of the KEGG-enriched pathway. Therefore, ATP6V1B2 was identified as the core target. Finally, immunoprecipitation (IP) experiments confirmed that combined stimulation with L-Lac and HDM significantly increased the lactylation level of endogenous ATP6V1B2 (Fig. 2I). In summary, we determined ATP6V1B2 as a key lactylated protein responding to high lactate environments and potentially regulating lysosomal function.

Furthermore, to ensure the robustness and non-cell-type-specific nature of this finding, we performed orthogonal validation in primary human bronchial epithelial cells (HBEs). Western Blot and IP analyses in HBEs consistently demonstrated that HDM challenge alone was sufficient to induce specific ATP6V1B2 lactylation, which was further enhanced by L-Lac co-treatment (Supplementary Fig. S2C and 2D).

### 3.3 Lactylation at ATP6V1B2 K108/K109 sites disrupts V-ATPase assembly and proton pump activity by restricting conformational dynamics

**Fig. 3:**
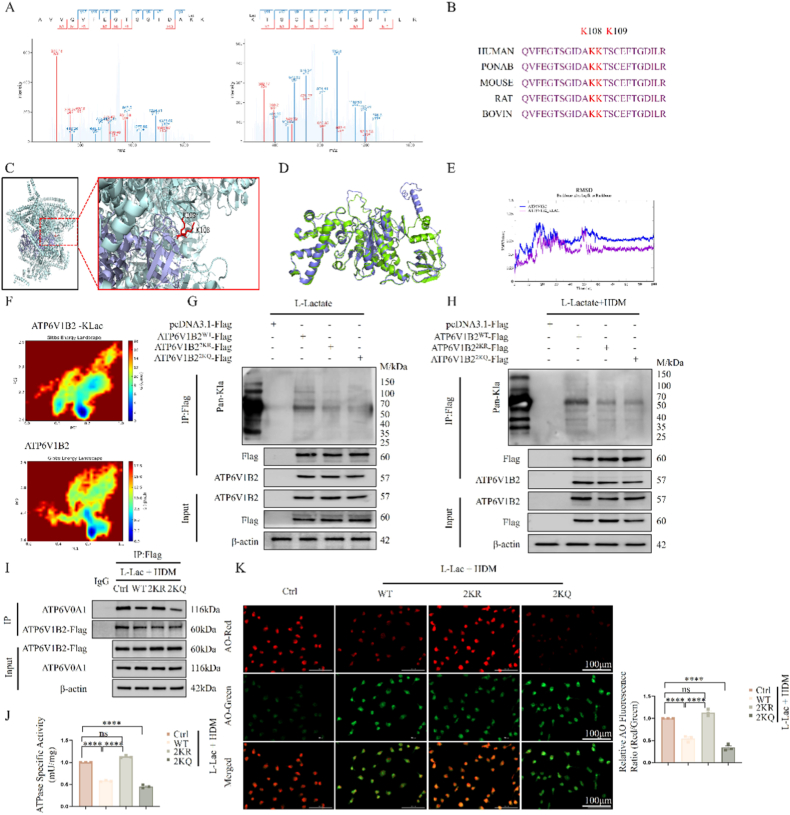
**Effect of lactylation at ATP6V1B2 K108/K109 sites on V-ATPase structure, assembly, and activity.** (A) MS/MS spectra of lactylated peptides at ATP6V1B2 K108 and K109 sites. (B) Amino acid sequence alignment of ATP6V1B2 across different species, red indicates K108/K109 sites. (C) Schematic diagram of ATP6V1B2 protein structural model and spatial positions of K108/K109 sites. (D) Superimposition of molecular dynamics (MD) simulation structures of wild-type (WT) and lactylated (KLac) ATP6V1B2. (E) Root-mean-square deviation (RMSD) analysis during MD simulation. (F) Gibbs free energy landscape (FEL) of WT and KLac models. (G, H) IP analysis of cells transfected with WT, 2 KR, 2KQ plasmids after L-Lac (G) or L-Lac + HDM (H) treatment, detecting lactylation levels of Flag-tagged proteins. (I) Co-IP detection of binding between ATP6V1B2-Flag (V1 subunit) and endogenous ATP6V0A1 (V0 subunit). (J) Quantitative detection of V-ATPase ATP hydrolysis activity in cells of each group. (K) Acridine orange (AO) staining detects lysosomal acidity (red: acidic lysosomes; green: cytosolic/DNA binding). Scale bar: 100 μm. The ratio of red to green fluorescence intensity was quantified to assess lysosomal acidification. Data are expressed as Mean ± SEM. (n = 3 independent experiments) ∗∗∗∗P < 0.0001; ns, not significant.

Building on our omics findings, we sought to delineate the specific sites of ATP6V1B2 lactylation and their structural and functional consequences. 4D-FastDIA analysis precisely pinpointed K108 and K109 as the key lactylation sites, with MS/MS spectra confirming the fidelity of this modification (Fig. 3A; Supplementary Fig. S2B). Sequence alignment and structural mapping revealed that these residues are highly conserved across species (Fig. 3B) and are situated in surface-exposed regions of the V-ATPase V1 domain (Fig. 3C; Supplementary Fig. S3A), suggesting a potential regulatory role in protein-protein interactions.

To elucidate the structural impact of lactylation at the atomic level, we performed 100 ns molecular dynamics (MD) simulations. The results demonstrated that introducing lactylation (KLAC) forced ATP6V1B2 to transition from a flexible wild-type (WT) state to a conformationally constrained state (Fig. 3D). Root-mean-square deviation (RMSD) analysis indicated significantly increased structural rigidity in the KLAC model (Fig. 3E). Furthermore, reductions in the radius of gyration (Rg) and root-mean-square fluctuation (RMSF) corroborated the lactylation-induced structural compaction and loss of local flexibility (Supplementary Fig. S3B–D). Finally, the Gibbs free energy landscape (FEL) revealed that while the WT protein occupies multiple local energy minima representing structural plasticity, the KLAC model is confined within a single thermodynamic basin (Fig. 3E), indicating a loss of the allosteric capacity required for function.

To validate these predictions, we generated a lactylation-deficient mutant (K108R/K109R, "2 KR") and a lactylation-mimetic mutant (K108Q/K109Q, "2KQ"). Immunoprecipitation confirmed that both mutants completely lost recognition by pan-lactylation antibodies (Fig. 3G and H), establishing K108/K109 as the predominant lactylation sites under these conditions.

Mechanistically, Co-IP analysis unveiled the disruptive effect of lactylation: combined HDM and L-Lac treatment significantly compromised the interaction between the V1 subunit (ATP6V1B2) and the V0 subunit (ATP6V0A1). Notably, the 2KQ mutant mimicked this disassembly, whereas the 2 KR mutant maintained holoenzyme integrity even under stimulation (Fig. 3I). To provide robust physical evidence of this dissociation in situ, we performed super-resolution quantitative co-localization imaging. As shown in Supplementary Fig. S3F, compared to the 2 KR mutant, the 2KQ mutant exhibited significantly reduced spatial co-localization between the V1 subunit (ATP6V1B2) and the V0 subunit (ATP6V0A1), confirmed by a marked decrease in the Pearson's Correlation Coefficient. Furthermore, endogenous Co-IP assays demonstrated that HDM challenge, and particularly l-lactate supplementation, significantly attenuated the interaction strength between endogenous V1 and V0 subunits (Supplementary Fig. S3G), proving that the asthmatic pathological microenvironment inhibits effective V-ATPase assembly. This assembly defect translated directly into functional impairment: the 2KQ mutant exhibited significantly reduced ATPase hydrolysis activity (Fig. 3J) and defective lysosomal acidification, as indicated by a decreased Red/Green ratio in acridine orange (AO) staining (Fig. 3K). To rigorously confirm that the functional defect induced by the 2KQ mutation is equivalent to protein loss-of-function, we optimized the proton-pumping assay by including ATP6V1B2 knockdown (siRNA) as a control (Supplementary Fig. S3H). Strikingly, the 2KQ mutant caused a significant elevation in lysosomal pH that was comparable to the degree observed in the ATP6V1B2 knockdown group, whereas the 2 KR mutant fully preserved acidification capacity. Consistent with this, HDM and l-lactate treatment caused a marked loss of red fluorescence in AO staining, signifying severe lysosomal alkalization. To dissect the specific contribution of l-lactate, we compared HDM alone versus the HDM/l-lactate combination. While HDM challenge alone induced moderate lysosomal de-acidification and mild mitochondrial fragmentation, l-lactate supplementation significantly exacerbated these defects (Supplementary Fig. S3I and 3J). This confirms that l-lactate acts as a critical synergistic factor, amplifying basal inflammatory stress into severe organelle dysfunction. Importantly, the 2 KR mutant was sufficient to completely resist L-Lac-induced enzyme inhibition and acidification failure. In summary, lactylation at ATP6V1B2 K108/K109 sites blocks correct V-ATPase assembly.

### 3.4 ATP6V1B2 lactylation triggers lysosomal acidification obstacles, membrane permeability changes (LMP), and content leakage

**Fig. 4:**
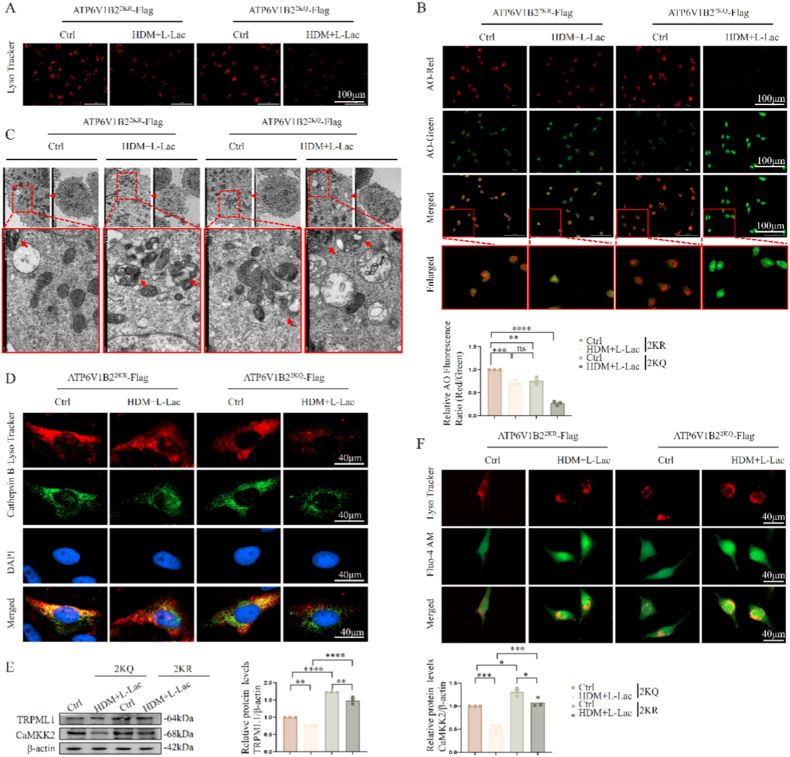
**ATP6V1B2 lactylation affects lysosomal acidification, membrane permeability (LMP), and calcium channels.** (A) Lysosomal fluorescence imaging labeled with LysoTracker Red probe.Scale bar: 100 μm. (B) Acridine orange (AO) staining detects LMP. Red box indicates magnified field. Scale bar: 100 μm. The red/green fluorescence ratio was quantified to evaluate lysosomal membrane permeabilization. (C) Transmission electron microscopy (TEM) images of lysosomal ultrastructure. Arrows indicate lysosomal morphology. (D) Immunofluorescence colocalization analysis of Cathepsin B (green) and LysoTracker (red). Scale bar: 40 μm. (E) Immunoblot analysis and quantification of lysosomal channel protein TRPML1 and its downstream CaMKK2. (F) Live-cell confocal imaging of intracellular calcium (Fluo-4 AM, green) and lysosomes (LysoTracker, red). Scale bar: 40 μm. Data are expressed as Mean ± SEM. (n = 3 independent experiments) ∗P < 0.05, ∗∗P < 0.01, ∗∗∗P < 0.001, ∗∗∗∗P < 0.0001.

Given the loss of V-ATPase proton pump function, we further investigated the direct impact of ATP6V1B2 K108/K109 lactylation on lysosomal homeostasis. First, we assessed lysosomal acidification capacity. LysoTracker tracing showed that compared to 2 KR (deficient) cells maintaining a normal acidic environment, 2KQ (mimetic) cells exhibited significant red fluorescence quenching and enlarged lysosomal volume (Fig. 4A), indicating severe lysosomal luminal alkalinization. This acidification obstacle further led to changes in lysosomal membrane permeability (LMP). Acridine orange (AO) staining analysis showed that 2KQ mutation caused lysosomes to shift from red fluorescence representing acidic integrity to green fluorescence representing cytosolic diffusion (inversion of red/green ratio) (Fig. 4B). Ultrastructural evidence provided by transmission electron microscopy (TEM) finally confirmed this: unlike the intact membrane structures in the 2 KR group, cells in the 2KQ group showed extensive lysosomal swelling, with some lysosomal membranes undergoing obvious physical rupture (Fig. 4C).

The direct consequence of LMP is the cytosolic leakage of lysosomal contents. Immunofluorescence imaging clearly revealed the spatial translocation of Cathepsin B: in 2 KR cells, Cathepsin B presented a typical lysosomal punctate pattern; whereas in 2KQ cells, its signal transformed into a diffuse cytosolic distribution (Fig. 4D), confirming protease leakage. We further confirmed that the contribution of l-lactate is synergistic. Consistent with the LMP data, immunofluorescence analysis showed that while HDM challenge alone induced mild Cathepsin B translocation, the addition of l-lactate significantly aggravated this leakage, confirming the synergistic role of l-lactate in driving severe lysosomal damage (Supplementary Fig. S4A).

Besides proteases, we found that lactylation disrupted lysosomal calcium homeostasis. Western Blot showed that 2KQ mutation significantly inhibited the expression of the lysosomal calcium release channel TRPML1 and its downstream effector molecule CAMKK2 (Fig. 4E). To clarify the regulatory mechanism behind TRPML1 downregulation, we performed qPCR and cycloheximide (CHX) chase assays. qPCR data confirmed that the 2KQ mutant did not alter TRPML1 mRNA levels (Supplementary Fig. S4B), suggesting regulation is post-transcriptional. However, the CHX chase assay revealed that TRPML1 protein stability was significantly reduced in 2KQ cells, resulting in a markedly shorter protein half-life (Supplementary Fig. S4C). This indicates that ATP6V1B2 lactylation promotes the post-translational degradation of TRPML1.

This downregulation of channel proteins resulted in the inability to normally release calcium ions. Colocalization analysis confirmed highly overlapping and significant accumulation of Ca^2+^ signals with lysosomal markers in 2KQ cells (Fig. 4F). In summary, ATP6V1B2 K108/K109 lactylation not only leads to lysosomal alkalinization and LMP but also specifically causes cytosolic leakage of Cathepsin B and TRPML1-mediated lysosomal calcium accumulation.

### 3.5 ATP6V1B2 lactylation triggers mitochondrial bioenergetic crisis and dynamic imbalance

**Fig. 5:**
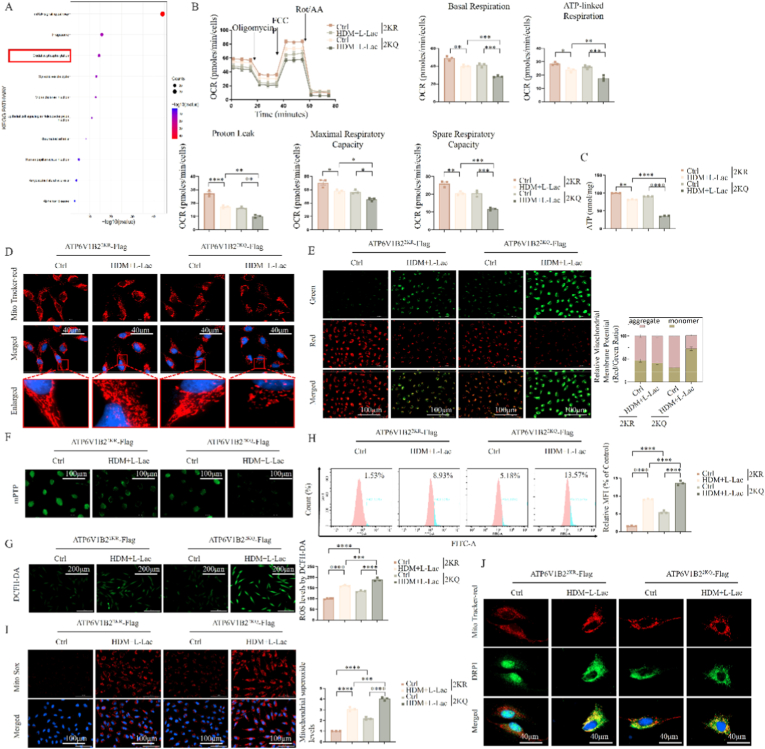
**Effect of ATP6V1B2 lactylation on mitochondrial respiratory function, morphology, and ROS levels.** (A) KEGG pathway enrichment analysis of ATP6V1B2 interaction network. (B) Seahorse XF mitochondrial stress test. Left is OCR real-time curve, right is quantitative analysis of respiratory parameters. (C) Measurement of intracellular ATP content. (D) Mitochondrial morphology observation via MitoTracker Red staining. Red box shows local magnification. Scale bar: 40 μm. (E) JC-1 probe detection of mitochondrial membrane potential (red aggregates/green monomers). Scale bar: 100 μm. The ratio of red (aggregates) to green (monomers) fluorescence was quantified to evaluate mitochondrial membrane potential. (F) Calcein-AM/CoCl_2_ assay detecting mitochondrial permeability transition pore (mPTP) opening. Scale bar: 100 μm. (G, H) Fluorescence imaging (G) and flow cytometric quantification (H) of intracellular total ROS using DCFH-DA probe. Scale bar: 200 μm. Mean fluorescence intensity (MFI) was quantified in (H). (I) MitoSOX Red probe detection of mitochondrial superoxide. Scale bar: 100 μm. Mitochondrial superoxide levels were quantified by measuring the mean fluorescence intensity (MFI). (J) Immunofluorescence colocalization analysis of DRP1 (green) and mitochondria (red). Scale bar: 40 μm. Data are expressed as Mean ± SEM. (n = 3 independent experiments) ∗P < 0.05, ∗∗P < 0.01, ∗∗∗P < 0.001.

To explore the downstream cascade effects triggered by ATP6V1B2 lactylation, we first performed protein-protein interaction network analysis based on the STRING database. KEGG enrichment analysis suggested a significant potential association between ATP6V1B2 and the "Oxidative Phosphorylation (OXPHOS)" pathway (Fig. 5A), implying its modification might distantly regulate mitochondrial function.

To verify this prediction, we utilized a Seahorse XF analyzer to evaluate mitochondrial respiratory function. Results showed that unlike 2 KR (deficient) cells maintaining normal respiratory curves, 2KQ (mimetic) mutation led to significant decreases in basal oxygen consumption rate (OCR), maximal respiratory capacity, and ATP production rate (Fig. 5B and C), indicating cells fell into a severe bioenergetic crisis.

This functional failure was accompanied by extensive destruction of mitochondrial structure. MitoTracker staining showed mitochondrial networks in 2KQ cells presenting hyper-fragmentation and swollen morphology, losing normal tubular network structures (Fig. 5D). Further functional tests confirmed loss of mitochondrial integrity: JC-1 probe showed 2KQ caused significant mitochondrial membrane potential depolarization (decreased red/green fluorescence ratio) (Fig. 5E); simultaneously, Calcein-AM quenching assay indicated pathological opening of the mitochondrial permeability transition pore (mPTP) (Fig. 5F).

Electron leakage and potential collapse typically lead to oxidative stress. Indeed, 2KQ mutation caused sharp elevations in intracellular total ROS (DCFH-DA) and mitochondrial superoxide (MitoSOX) levels (Fig. 5G–I). Finally, colocalization analysis revealed 2KQ significantly promoted the recruitment of fission DRP1 to mitochondria (Fig. 5J), leading to uncontrolled mitochondrial fission.

Additionally, to further substantiate the involvement of DRP1 in mitochondrial fission, we performed Western Blot analysis on DRP1 phosphorylation status (Supplementary Fig. S5). We found that the 2KQ mutant significantly promoted DRP1 activation by increasing the phosphorylation level at the pro-fission site Ser616. This molecular evidence strongly supports that ATP6V1B2 lactylation directly drives DRP1-mediated hyper-fragmentation.

In summary, ATP6V1B2 K108/K109 lactylation leads to comprehensive mitochondrial dysfunction by disrupting oxidative phosphorylation, inducing potential collapse, and DRP1-mediated hyper-fragmentation.

### 3.6 Lysosomal cathepsin B leakage drives mitochondrial dysfunction by activating the tBid-Bax axis

**Fig. 6:**
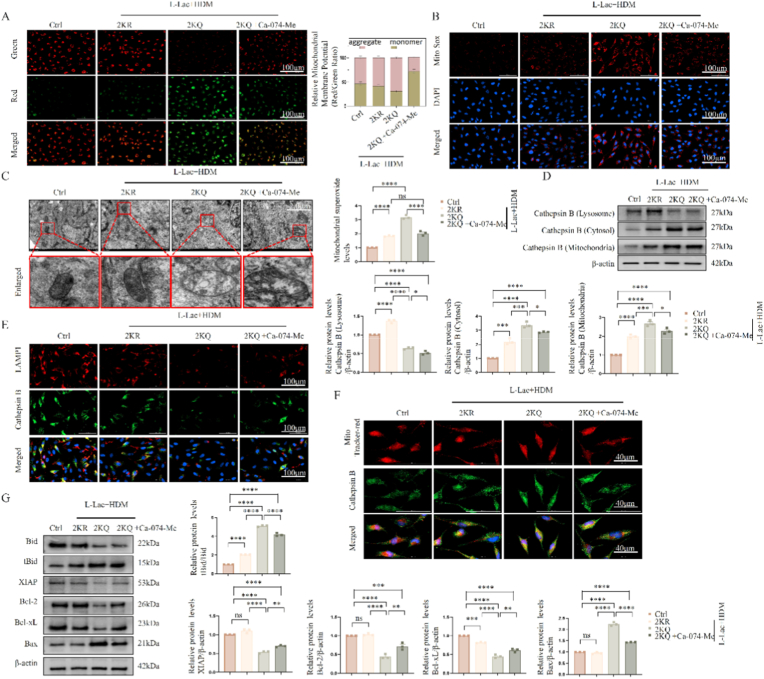
**Effect of inhibiting Cathepsin B on mitochondrial dysfunction and tBid-Bax pathway.** (A) Fluorescence imaging of mitochondrial membrane potential (JC-1) after administration of Cathepsin B inhibitor (Ca-074-Me). Scale bar: 100 μm. The ratio of red/green fluorescence intensity was quantified. (B) MitoSOX Red staining detects mitochondrial ROS. Scale bar: 100 μm. Quantification of MitoSOX Red fluorescence intensity is shown. (C) TEM images of mitochondrial ultrastructure. Scale bar: 2.0 μm. (D) Immunoblot analysis of Cathepsin B distribution in subcellular fractions (lysosome, cytosol, mitochondria). (E) Immunofluorescence staining of Cathepsin B (green) and LAMP1 (red). Scale bar: 100 μm. (F) Immunofluorescence staining of Cathepsin B (green) and MitoTracker (red). Scale bar: 40 μm. (G) Immunoblot analysis of Bid, tBid, XIAP, Bcl-2, Bcl-xL, and Bax protein expression. Data are expressed as Mean ± SEM. (n = 3 independent experiments) ∗P < 0.05, ∗∗P < 0.01, ∗∗∗P < 0.001, ∗∗∗∗P < 0.0001; ns, not significant.

To elucidate the molecular hierarchical relationship between lysosomal damage and mitochondrial dysfunction, we investigated the Cathepsin B-mediated "Lysosomal-Mitochondrial Axis." First, we used the Cathepsin B-specific inhibitor Ca-074-Me to block its activity. Results showed that Ca-074-Me significantly rescued 2KQ mutant-induced mitochondrial phenotypes, including restoring mitochondrial membrane potential (Fig. 6A), curbing mitochondrial ROS burst (Fig. 6B), and improving swollen mitochondrial ultrastructure (Fig. 6C).

Mechanistically, subcellular fractionation (Fig. 6D) and immunofluorescence colocalization (Fig. 6E and F) analyses consistently confirmed that Ca-074-Me effectively blocked the pathological translocation of Cathepsin B from lysosomes to the cytoplasm. Blocking this translocation subsequently cut off downstream lethal signals: Western Blot showed that in 2KQ cells, leaked Cathepsin B mediated the proteolytic processing of Bid protein, generating truncated Bid (tBid).

To further substantiate the direct involvement of Cathepsin B in Bid cleavage, we performed an immunoprecipitation (Co-IP) assay to assess the physical interaction between endogenous Cathepsin B and Bid. Experimental results (Supplementary Fig. S6A) demonstrated an enhanced binding affinity between Cathepsin B and Bid in 2KQ mutant cells compared to 2 KR cells, coinciding with the occurrence of LMP. This finding supports the model that Cathepsin B, upon leakage into the cytosol, makes contact with and acts as a protease to cleave Bid. tBid, as a pro-apoptotic messenger, further translocated to mitochondria, disrupting Bcl-2/Bax balance and downregulating the anti-apoptotic protein XIAP. Ca-074-Me treatment completely reversed this process (Fig. 6G).

To exclude drug off-target effects and confirm this mechanism genetically, we used Bid-specific siRNA for genetic silencing. Results were highly consistent with pharmacological inhibition: knockdown of Bid or use of a Bid inhibitor (BI–6C9) could significantly block 2KQ-induced mitochondrial membrane potential collapse, ROS accumulation, and Cytochrome C release (Supplementary Fig. S6B–E). In summary, these orthogonal verification data strongly prove that Cathepsin B cytosolic leakage caused by ATP6V1B2 lactylation is an upstream initiating event, which cascades to induce downstream mitochondrial damage by activating the tBid-Bax signal axis.

### 3.7 Lysosomal calcium leakage leads to cytosolic calcium overload and secondary mitochondrial calcium toxicity

Besides Cathepsin B, we further confirmed that calcium homeostasis imbalance is another parallel critical mechanism connecting lysosomal damage and mitochondrial failure. Based on previous findings (Fig. 4E and F), ATP6V1B2 lactylation (2KQ) inhibited TRPML1 channel expression, leading to pathological accumulation of calcium ions within the lysosomal lumen. We hypothesized that subsequent lysosomal membrane rupture (LMP) resulted in uncontrolled release of these sequestered high-concentration calcium ions.

**Fig. 7:**
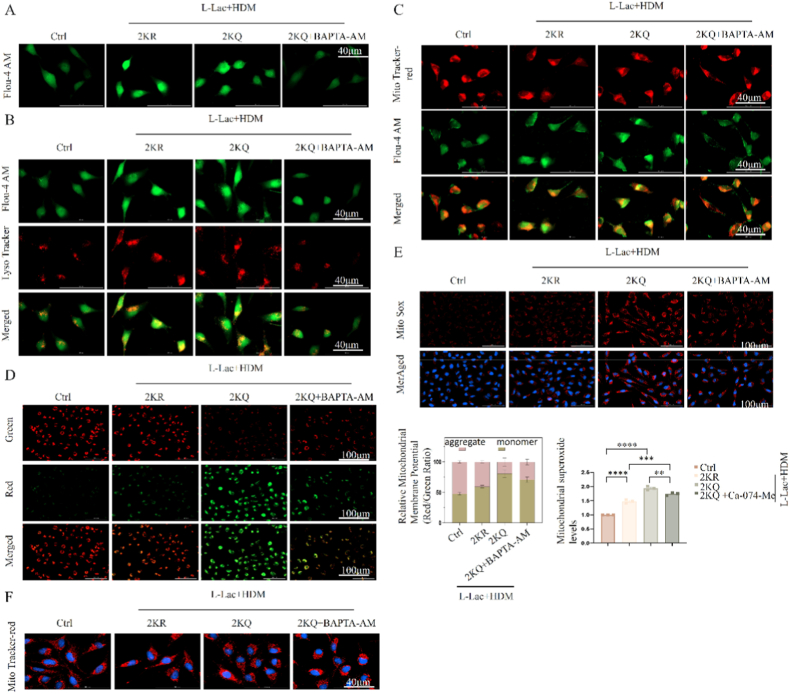
**Detection of cytosolic calcium overload and mitochondrial calcium uptake.** (A) Fluo-4 AM probe detecting cytosolic calcium concentration. BAPTA-AM is a calcium chelator. Scale bar: 40 μm. (B) Dual staining of Fluo-4 AM (green) and LysoTracker (red). Scale bar: 40 μm. (C) Colocalization analysis of MitoTracker Red and Fluo-4 AM, showing mitochondrial calcium levels. Scale bar: 40 μm. (D) JC-1 mitochondrial membrane potential detection after BAPTA-AM treatment. Scale bar: 100 μm. The red/green fluorescence ratio was quantified. (E) MitoSOX Red mitochondrial ROS detection. Scale bar: 100 μm. Quantification of fluorescence intensity is shown. (F) MitoTracker Red mitochondrial morphology detection. Scale bar: 40 μm. (n = 3 independent experiments).

Fluo-4 AM calcium imaging confirmed this hypothesis: 2KQ cells exhibited significant cytosolic calcium overload (Fig. 7A). Crucially, colocalization analysis revealed the source of calcium ions; high-intensity cytosolic calcium signals (green) highly overlapped spatially with functionally impaired lysosomes (red) (Fig. 7B), intuitively tracing the trajectory of calcium leakage from ruptured lysosomes to the cytoplasm.

**Fig. 8:**
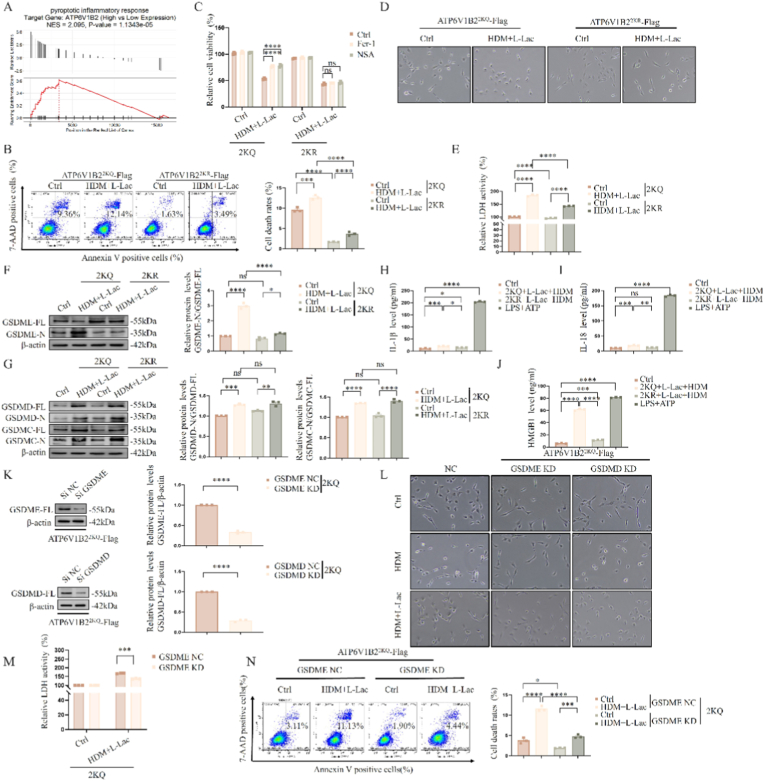
**Identification of cell death type mediated by ATP6V1B2 lactylation and GSDME pathway analysis.** (A) GSEA enrichment analysis plot of ATP6V1B2 high-expression samples. (B) Annexin V/7-AAD double staining flow cytometry detecting cell death rate. (C) Cell viability (CCK-8) analysis after treatment with ferroptosis inhibitor (Fer-1) and necroptosis inhibitor (NSA). (D) Cellular morphology observation under bright-field microscope. Scale bar: 100 μm. (E) LDH release rate detection. (F, G) Immunoblot analysis of GSDME (F) and GSDMD/GSDMC (G) cleavage. (H–J) ELISA detection of IL-1β (H), IL-18 (I), and HMGB1 (J) in cell supernatants. LPS + ATP as positive control. (K) Verification of GSDME and GSDMD siRNA knockdown efficiency. (L) Cellular morphology observation after knockdown of GSDME or GSDMD. (M, N) Analysis of LDH release (M) and cell death rate (N) after knockdown of GSDME. Data are expressed as Mean ± SEM. (n = 3 independent experiments) ∗P < 0.05, ∗∗∗P < 0.001, ∗∗∗∗P < 0.0001; ns, not significant.

This cytosolic calcium surge rapidly affected mitochondria. Co-staining of mitochondrial calcium probe with MitoTracker showed that compared to the 2 KR group, 2KQ cells exhibited significantly increased mitochondrial calcium uptake (Fig. 7C). This mitochondrial calcium overload is severe as it disrupts the electron transport chain and leads to membrane potential collapse.

To confirm the causal role of calcium overload, we used the intracellular calcium-specific chelator BAPTA-AM. Results showed that clearing excess cytosolic calcium could significantly rescue 2KQ-induced mitochondrial pathological phenotypes, including restoring mitochondrial membrane potential (JC-1) (Fig. 7D), inhibiting mitochondrial ROS (MitoSOX) generation (Fig. 7E), and reversing mitochondrial swelling and fragmentation (Fig. 7F). In summary, these data established a specific pathological cascade reaction where "TRPML1 inhibition or LMP occurrence leads to cytosolic calcium overload and mitochondrial calcium toxicity."

### 3.8 ATP6V1B2 lactylation specifically drives GSDME-dependent non-canonical pyroptosis

Given that lysosomal damage often triggers lethal signals, we first explored the association between ATP6V1B2 and cell death modes through single-gene GSEA analysis. Results showed that the "Pyroptotic inflammatory response" pathway was significantly enriched in ATP6V1B2 high-expression samples (Fig. 8A), suggesting a positive correlation between the two.

To enhance clinical relevance, we further analyzed the publicly available human bronchial epithelial cell transcriptome dataset (GEO: GSE43696) to correlate our key molecules with clinical disease severity. We first investigated the expression of the pyroptosis execution molecule, GSDME (DFNA5). GSDME mRNA expression was significantly upregulated in severe asthma patients compared to healthy controls (Supplementary Fig. S7A), suggesting that pyroptosis is actively involved in the pathogenesis of severe asthma. In contrast, the expression level of ATP6V1B2 mRNA did not show significant differences across control, moderate, and severe asthma patient groups (Supplementary Fig. S7B). This translational finding suggests that the pathological function of ATP6V1B2 in human asthma is primarily regulated at the post-translational modification level (lactylation), rather than transcriptional abundance, which strongly aligns with the core mechanism presented in our study.

To verify this prediction, we identified the type of death induced by ATP6V1B2 lactylation (2KQ) through pharmacological screening. Flow cytometry showed that the 2KQ mutant significantly exacerbated L-Lac + HDM induced cell death (Annexin V+/7-AAD+) (Fig. 8B). Crucially, neither ferroptosis inhibitor (Ferrostatin-1) nor necroptosis inhibitor (Necrosulfonamide) could save cells, whereas blocking lactylation (2 KR) completely restored cell survival (Fig. 8C). Morphologically, 2KQ cells exhibited typical pyroptotic features, namely cellular ballooning and membrane rupture (Fig. 8D), accompanied by massive release of cytosolic LDH (Fig. 8E).

At the molecular mechanism level, Western Blot revealed the specificity of pyroptosis execution proteins: the 2KQ mutant specifically induced cleavage of the GSDME protein N-terminal pore-forming domain (GSDME-N), without significant impact on GSDMD or GSDMC (Fig. 8F and G). This indicates that ATP6V1B2 lactylation activates the GSDME pathway.

Further ELISA analysis ruled out the involvement of classical inflammasomes. Unlike LPS + ATP (classical NLRP3 activator), 2KQ treatment did not induce maturation and secretion of IL-1β and IL-18, but significantly released a key damage-associated molecular pattern (DAMP)—HMGB1 (Fig. 8H–J). This feature confirmed the process is a non-canonical pyroptosis independent of the NLRP3-Caspase-1 axis.

Finally, we confirmed the central role of GSDME through genetic knockdown experiments (Fig. 8K). Specific silencing of GSDME (siRNA-GSDME), but not GSDMD, significantly reversed 2KQ-induced pyroptotic phenotypes, including reducing the number of ballooning cells (Fig. 8L), decreasing LDH release (Fig. 8M), and inhibiting cell death (Fig. 8N). In summary, these results established that ATP6V1B2 K108/K109 lactylation drives a proinflammatory non-canonical pyroptosis by activating GSDME (rather than GSDMD).

### 3.9 Caspase-8/3 proteolytic cascade mediates GSDME activation and pyroptosis

**Fig. 9:**
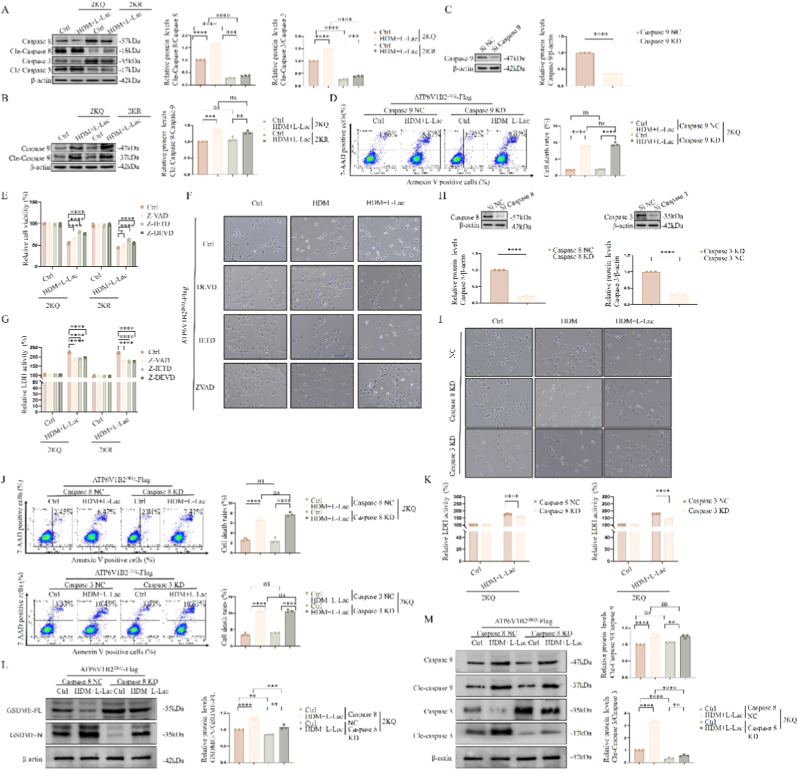
**Hierarchy analysis of Caspase family in GSDME activation and pyroptosis.** (A, B) Immunoblot detection of cleavage activation of Caspase-8, Caspase-3 (A) and Caspase-9 (B). (C, D) Knockdown efficiency verification (C) and cell death flow cytometric analysis (D) after knockdown of Caspase-9. (E–G) Cell viability (E), morphology (F), and LDH release (G) after treatment with Caspase inhibitors (Z-VAD, Z-IETD, Z-DEVD). (H) Verification of Caspase-8 and Caspase-3 siRNA knockdown efficiency. (I–K) Cell morphology (I), death rate (J), and LDH release (K) after knockdown of Caspase-8 or Caspase-3. (L) Immunoblot analysis of GSDME cleavage after knockdown of Caspase-8. (M) Immunoblot analysis of Caspase-9/3 cleavage after knockdown of Caspase-8. Data are expressed as Mean ± SEM. (n = 3 independent experiments) ∗∗P < 0.01, ∗∗∗P < 0.001, ∗∗∗∗P < 0.0001; ns, not significant.

After determining GSDME as the pyroptosis execution molecule, we further tracked its upstream protease activation mechanism. Western Blot analysis showed the 2KQ mutant significantly induced cleavage activation of Caspase-8 and Caspase-3 (Fig. 9A). Notably, although we observed mitochondrial dysfunction in previous experiments (Fig. 5, Fig. 6, Fig. 7), Caspase-9, the initiator enzyme of the mitochondrial intrinsic apoptosis pathway, did not undergo cleavage (Fig. 9B). Genetic knockdown experiments further confirmed this: specific silencing of Caspase-9 failed to rescue 2KQ-induced cell death (Fig. 9C and D), indicating this pyroptosis process is independent of the intrinsic apoptotic pathway.

Conversely, the Caspase-8/3 axis showed absolute necessity. Pharmacological blockade experiments showed that pan-Caspase inhibitor (Z-VAD), Caspase-8 specific inhibitor (Z-IETD), and Caspase-3 specific inhibitor (Z-DEVD) could all significantly reverse 2KQ-induced pyroptotic phenotypes, including restoring cell viability (Fig. 9E), reducing ballooning (Fig. 9F), and decreasing LDH release (Fig. 9G).

Genetic loss-of-function experiments further established the hierarchy of this pathway. Specific knockdown of Caspase-8 or Caspase-3 not only significantly inhibited pyroptosis and LDH release (Fig. 9H–K), crucially, they both completely blocked downstream GSDME cleavage (Fig. 9L). Further molecular ordering analysis showed that silencing upstream Caspase-8 significantly inhibited downstream Caspase-3 activation (Fig. 9M). Crucially, the reverse experiment demonstrated that specific silencing of Caspase-3 failed to affect the upstream activation and cleavage of Caspase-8 (Supplementary Fig. S8).

In summary, these results constructed a clear signal axis: ATP6V1B2 lactylation initiates the Caspase-8/Caspase-3 cascade reaction, subsequently specifically cleaving GSDME to execute pyroptosis.

### 3.10 Lysosomal dysfunction is the central node event initiating the caspase-8/3/GSDME pyroptosis cascade

Given the non-necessity of Caspase-9 (Fig. 9B), we aimed to find the initiating spark located upstream of mitochondria that directly ignites the Caspase-8/GSDME axis. Through systematic screening, we identified the lysosome as the core link.

**Fig. 10:**
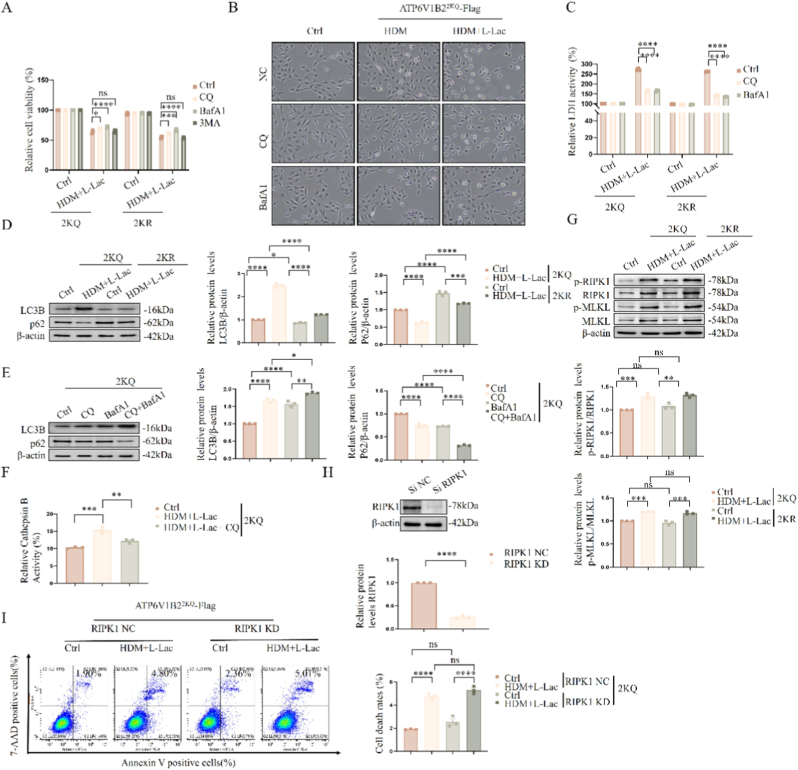
**Assessment of roles of lysosomal dysfunction, autophagy, and necroptosis in cell death.** (A–C) Cell viability (A), morphology (B), and LDH release (C) after treatment with lysosomal drugs (CQ, BafA1) or autophagy inhibitor (3-MA). (D, E) Immunoblot analysis of autophagy markers LC3B and p62 under different treatment conditions. (F) Intracellular Cathepsin B activity was measured using a fluorometric assay kit. Cells were treated with Chloroquine (CQ, 10 μM) for 24 h. (G) Immunoblot analysis of necroptosis pathway proteins (RIPK1, MLKL and their phosphorylated forms). (H) RIPK1 siRNA knockdown efficiency verification. (I) Flow cytometric analysis of cell death after knockdown of RIPK1. Data are expressed as Mean ± SEM. (n = 3 independent experiments) ∗P < 0.05, ∗∗P < 0.01, ∗∗∗P < 0.001, ∗∗∗∗P < 0.0001; ns, not significant.

Results showed that only interventions targeting lysosomes exhibited significant protective effects. Lysosomotropic agents—including V-ATPase inhibitor Bafilomycin A1 (BafA1) and lysosomal alkalinizing agent Chloroquine (CQ)—significantly rescued 2KQ-induced loss of cell viability (Fig. 10A), reversed ballooning morphology (Fig. 10B), and blocked LDH release (Fig. 10C). These data powerfully prove that lysosomal homeostasis collapse is the control switch for this lethal signal pathway.

To exclude other possible mechanisms, we rigorously evaluated the roles of autophagy and necroptosis through orthogonal experiments. Although 2KQ treatment induced compensatory enhancement of autophagic flux—manifested as increased LC3-II, accompanied by substrate p62 degradation (Fig. 10D), and further accumulation of LC3-II and p62 after combined BafA1 or CQ treatment (Fig. 10E, confirming smooth autophagic flux), to validate the mechanism of CQ-induced accumulation, we assessed lysosomal enzymatic activity. As shown in Fig. 10F, treatment with CQ resulted in a marked reduction in the enzymatic activity of Cathepsin B in BEAS-2B cells. This inhibition of lysosomal protease activity aligns with the accumulated protein levels observed in the immunoblot analysis, confirming that lysosomal dysfunction is responsible for the blockade of degradation.

However, blocking autophagy initiation (3-MA) could not rescue cell death (Fig. 10A). This indicates that autophagy enhancement is a cellular protective/concomitant response (epiphenomenon) to lysosomal stress, not the cause of death. Furthermore, we noted phosphorylation activation of necroptosis markers *p*-RIPK1 and *p*-MLKL occurred in the 2KQ group (Fig. 10G). However, this activation also existed in the non-lethal 2 KR group (Fig. 10F–ns), and specific knockdown of RIPK1 (Fig. 10H) could not reduce 2KQ-induced cell death (Fig. 10I). This ruled out the possibility of the RIPK1/MLKL pathway acting as death executioners.

In summary, by sequentially excluding mitochondria (intrinsic apoptosis), autophagic cell death, and necroptosis, we established that lysosomal dysfunction caused by ATP6V1B2 lactylation is the apex trigger event of this lethal cascade reaction. It not only causes mitochondrial damage but also directly initiates downstream pyroptosis.

### 3.11 Blocking ATP6V1B2 lactylation breaks the metabolic-inflammatory vicious cycle and alleviates asthma pathology

To confirm the pathological significance of ATP6V1B2 K108/K109 lactylation in vivo, we utilized AAV9 vectors to perform gain-of-function (2KQ) and loss-of-function (2 KR) gene intervention experiments in mouse lungs.

**Fig. 11:**
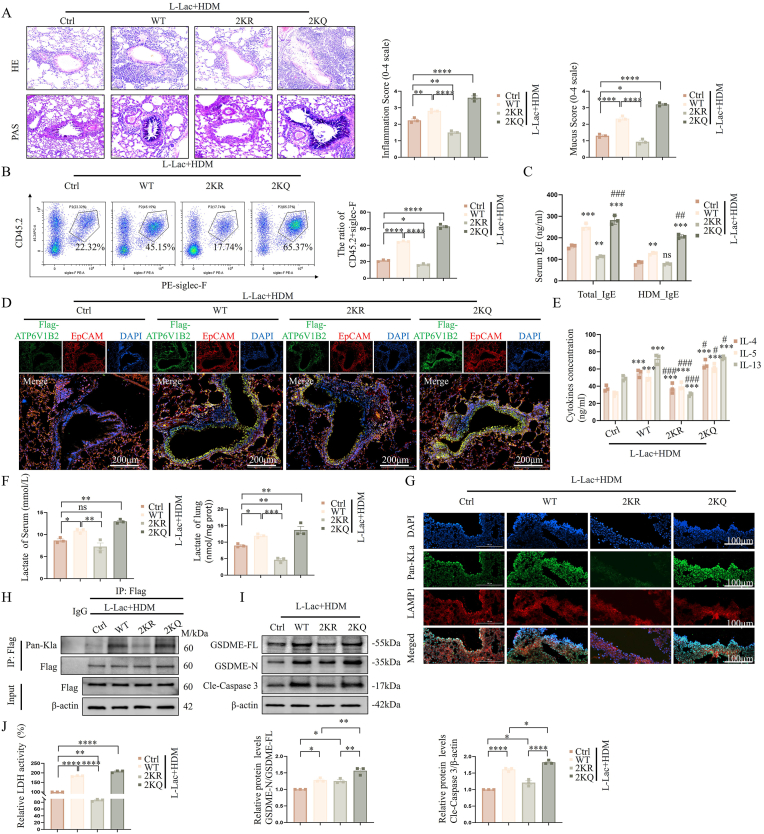
**Effect of blocking ATP6V1B2 lactylation on mouse asthma phenotypes and in vivo pyroptosis signals.** (A) H&E and PAS staining and scoring of AAV-transduced mouse lung tissues. (B) Flow cytometric analysis of eosinophils (CD11C^−^CD45.2^+^Siglec-F^+^) in BALF. (C) Detection of serum total IgE and HDM-specific IgE levels. (D) ELISA analysis of Th2 cytokines (IL-4, IL-5, IL-13) in BALF. (E) Measurement of lactate levels in serum and lung tissue homogenates. (F) Immunofluorescence staining of in situ Pan-Kla (green) and LAMP1 (red) in lung tissue frozen sections. Scale bar: 100 μm. (G) Immunoblot analysis of GSDME and Caspase-3 cleavage in lung tissue. (H) Lung tissue LDH activity detection. Data are expressed as Mean ± SEM. n = 10 per group. ∗P < 0.05, ∗∗P < 0.01, ∗∗∗P < 0.001, ∗∗∗∗P < 0.0001; ns, not significant.

In the potent L-Lac combined with HDM sensitization model, mice expressing wild-type (AAV-WT) or lactylation-mimetic (AAV-2KQ) ATP6V1B2 both exhibited severe asthma phenotypes. In sharp contrast, in vivo blockade of ATP6V1B2 lactylation (AAV-2KR) provided significant tissue protective effects. AAV-2KR group mice showed significantly reduced lung tissue pathological scores, decreased airway mucus secretion (Fig. 11A), and substantially reduced eosinophil (Siglec-F^+^) infiltration in BALF (Fig. 11B). Immunological analysis confirmed AAV-2KR effectively curbed systemic allergic responses, manifested as significant regression of serum total IgE and HDM-specific IgE levels (Fig. 11C).

To clarify the cellular context of these protective effects and the cell-type specificity of AAV delivery, we performed immunofluorescence co-staining of Flag (AAV-tag) and EpCAM (epithelial marker) in lung tissues. Consistent with the reported superior tropism of intranasal AAV9 for the respiratory tract (Yang et al., 2025), our results showed that Flag signals were predominantly localized within the EpCAM-positive airway epithelial layer (Fig. 11D). This confirms that the AAV-delivered 2 KR mutant primarily targets airway epithelial cells rather than myeloid cells, identifying the epithelium as the key site where ATP6V1B2 lactylation drives asthmatic inflammation.

At the molecular mechanism level, AAV-2KR significantly curbed hyperactivation of the Th2 inflammation cascade by blocking pyroptosis-mediated DAMPs (such as HMGB1) release. ELISA results showed that compared to AAV-WT/2KQ groups, IL-4, IL-5, and IL-13 levels in BALF of the AAV-2KR group were significantly suppressed (Fig. 11E). Notably, we observed an interesting metabolic feedback regulation phenomenon. Although all groups received the same dose of exogenous L-Lac challenge, lactate levels in lung tissue and serum of the AAV-2KR group were significantly lower than controls (Fig. 11F). This suggests inflammation driven by ATP6V1B2 lactylation might conversely promote glycolytic metabolism, and blocking this modification successfully severed this "metabolic-inflammatory positive feedback loop."

Finally, we verified the state of the in vivo pyroptosis pathway. Immunofluorescence analysis showed AAV-2KR significantly maintained lysosomal integrity (LAMP1 recovery), reduced global lactylation levels (Pan-Kla) (Fig. 11G). Furthermore, to rigorously quantify the site-specific inhibition in vivo, we conducted endogenous Co-IP assays in lung tissues. The results demonstrated that the lactylation level of ATP6V1B2 protein in the AAV-2KR group was significantly lower than that in the AAV-WT/2KQ group (Fig. 11H), confirming the precise attenuation of this modification at the biochemical level. Consequently, AAV-2KR effectively blocked downstream activation cleavage of Caspase-3/GSDME and LDH release (Fig. 11I and J).

In summary, these in vivo data provide decisive evidence that lactylation at ATP6V1B2 K108/K109 sites is a key molecular switch driving asthmatic airway inflammation and GSDME pyroptosis, and blocking modification at these sites holds significant therapeutic potential.

## 4 Discussion

**Fig. 12:**
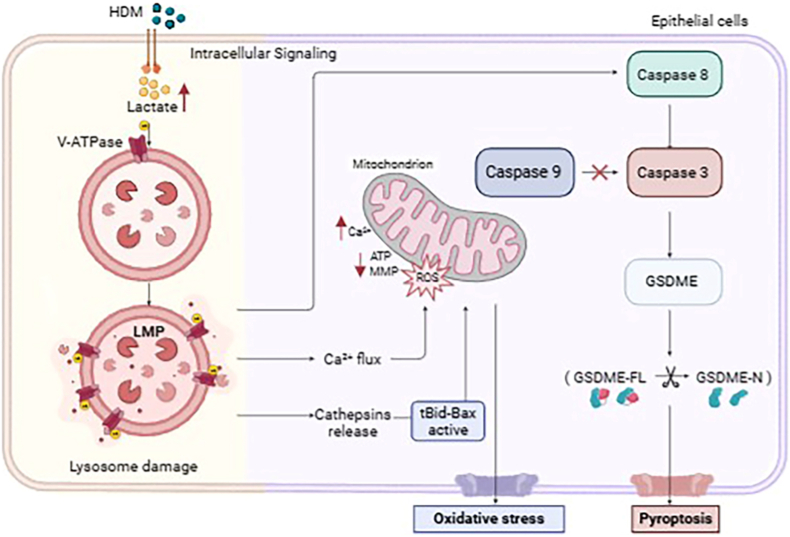
Schematic model of the mechanism by which l-lactate drives lysosomal dysfunction and GSDME-dependent pyroptosis via ATP6V1B2 lactylation. HDM induces intracellular l-lactate accumulation, triggering lactylation at ATP6V1B2 K108/K109 sites, leading to disruption of V-ATPase assembly and lysosomal membrane permeabilization (LMP). LMP initiates two downstream pathways: (1) lysosomal Cathepsin B leakage activates the tBid-Bax axis, resulting in mitochondrial damage; (2) initiation of a non-canonical Caspase-8/3 cascade reaction, specifically cleaving GSDME to execute pyroptosis, thereby exacerbating airway inflammation.

This study is the first to systematically reveal the core driving role of l-lactate metabolic abnormality in asthma pathogenesis and map a complete molecular landscape from metabolic reprogramming to immune inflammation: "l-lactate accumulation - ATP6V1B2 key site lactylation - lysosomal dysfunction - GSDME-dependent pyroptosis." The findings suggest that targeting ATP6V1B2 lactylation modification could be a potential strategy to alleviate airway inflammation and block pyroptosis cascades (Fig. 12).

Our data confirm l-lactate as a key metabolic factor driving chronic airway inflammation. HDM-induced asthma models are accompanied by a sharp rise in lung tissue lactate levels, while exogenous L-Lac supplementation or inhibition of endogenous lactate generation (using Oxamate) can bi-directionally regulate airway inflammation, eosinophil infiltration, and IgE production. This finding resonates with recent reports regarding lactate increasing vascular permeability in septic acute lung injury (ALI) [29], supporting the view of lactate as a key proinflammatory mediator. To fully substantiate the role of l-lactate as a primary signal, we performed intranasal (i.n.) lactate administration, which confirmed that direct local airway exposure to l-lactate similarly exacerbates inflammatory and pathological phenotypes, strongly validating the robustness of our conclusions regarding lactate's local contribution.

The core innovation of this study lies in identifying ATP6V1B2 as a key downstream effector protein sensing high lactate environments. Although global upregulation of lactylation exists in asthma models, our quantitative proteomics analysis was primarily employed as an unbiased discovery tool in the defined L-Lac-stimulated cell system to pinpoint core modification targets. Quantitative proteomics pinpointed ATP6V1B2 as a core target. Molecular dynamics simulations and biochemical experiments showed lactylation at K108/K109 sites restricts ATP6V1B2 conformational flexibility, hindering correct assembly of the V-ATPase complex. To rigorously validate this causality between lactylation and complex disassembly, we employed orthogonal approaches. Recognizing the technical limitations of extraction-based methods like BN-PAGE in maintaining the integrity of large transmembrane complexes, we utilized super-resolution quantitative co-localization imaging as a robust in situ alternative. Our data provided direct physical evidence that the 2KQ mutant exhibits significantly reduced spatial association with the V0 subunit compared to the 2 KR mutant. This was further corroborated by endogenous Co-IP assays, which demonstrated that the pathological combination of HDM and l-lactate significantly weakens the interaction between endogenous V1 and V0 subunits. Crucially, by comparing the 2KQ mutant with ATP6V1B2 knockdown (siRNA) in proton-pumping assays, we demonstrated functional equivalence: the degree of lysosomal alkalinization induced by lactylation was comparable to that of protein loss-of-function. Collectively, these data confirm that K108/K109 lactylation is the direct cause of V-ATPase dysfunction and lysosomal alkalinization. This is similar to pathogenic mechanisms of elevated lysosomal pH observed in lymphoma [30] and Alzheimer's disease [20]. Studies have shown that gain-of-function mutations in ATP6V1B2 causing hyper-acidification are also pathogenic [24,31], suggesting that maintaining strict lysosomal pH homeostasis is crucial for cell survival.

Although our global lactylome analysis identified several lysosome-associated proteins with altered modification levels, a stringent screening strategy highlighted ATP6V1B2 as the dominant functional target. Bioinformatically, ATP6V1B2 exhibited a specific 'modification hotspot' pattern, characterized by synchronized hyper-lactylation at the adjacent K108 (Ratio = 1.88, P < 0.05) and K109 (Ratio = 1.60, P < 0.05) residues. This stands in sharp contrast to other high-abundance candidates such as HSPA8, which displayed significant downregulation (K601, Ratio = 0.58, P < 0.001) despite the high-lactate microenvironment. Functionally, unlike the chaperone role of HSPA8 in protein folding, ATP6V1B2 serves as the catalytic engine of the V-ATPase complex. Consequently, its specific hyper-lactylation provides the most direct mechanistic link to the observed lysosomal acidification failure and subsequent GSDME-dependent pyroptosis.

Importantly, to address concerns regarding cell type specificity, we performed orthogonal validation in primary human bronchial epithelial cells (HBEs). The results confirmed that HDM challenge alone is sufficient to induce ATP6V1B2 lactylation, which is further amplified by exogenous L-Lac (Fig. 3A). This finding decisively establishes the clinical relevance and robustness of ATP6V1B2 as the core lactylation target in human airway epithelial cells under asthmatic conditions. The subsequent in vivo AAV-2KRrescue data (Fig. 11) provides definitive evidence that this specific modification drives the full cascade of asthmatic inflammation in the whole organism.

We further confirmed that ATP6V1B2 lactylation-induced lysosomal membrane permeabilization (LMP) is the upstream initiating event for subsequent lethal signals. To distinguish the specific contribution of l-lactate from HDM basal stimulation, we demonstrated that while HDM challenge alone induced moderate lysosomal de-acidification and mild mitochondrial dysfunction, the supplementation of l-lactate acted as a synergistic amplifier, significantly exacerbating these defects. The occurrence of LMP leads to two parallel pathological processes: First, LMP causes Cathepsin B release and activates the tBid-Bax axis, triggering mitochondrial dysfunction. We focused on Cathepsin B because of its unique ability to cleave the pro-apoptotic protein BID into tBID upon cytosolic leakage, a critical mechanism linking LMP to mitochondrial outer membrane permeabilization MOMP [32,33].

This result supports the view that the V-ATPase B2 subunit is crucial for maintaining lysosomal membrane integrity [25] and explains mitochondrial-related apoptotic phenotypes caused by ATP6V1B2 mutations [23]. Furthermore, the downregulation of the lysosomal calcium channel TRPML1, which contributes to calcium dyshomeostasis, was demonstrated to occur at the post-translational level, as qPCR showed no change in mRNA, while the protein half-life was significantly reduced in 2KQ cells. Second, lysosomal damage directly initiates GSDME-dependent pyroptosis. Unlike the classical NLRP3-GSDMD pathway [34], we confirmed this process relies on the Caspase-8/3/GSDME axis, leading to the release of damage-associated molecular patterns (DAMPs, such as HMGB1) [35,36].

Although we observed clear tBid mitochondrial translocation and cytochrome *c* release, the classical intrinsic apoptosis initiator enzyme Caspase-9 surprisingly remained silent (Fig. 9B), and its gene silencing failed to rescue cell death. This seems contrary to the traditional "MOMP - Apaf-1 - Caspase-9″ apoptosis paradigm. We believe a severe bioenergetic crisis is the key mechanism causing this " signaling diversion" and determining death fate. Correct assembly of the apoptosome and activation of Caspase-9 is a highly ATP/dATP-dependent process [37,38]. Our data clearly show synergistic effects of V-ATPase loss-of-function triggered by ATP6V1B2 lactylation and subsequent mitochondrial damage, leading to a severe decline in intracellular ATP production rates (Fig. 5C). This extreme ATP depletion likely physically prevents Apaf-1 oligomerization and subsequent Caspase-9 recruitment, effectively "cutting off" energy-dependent classical apoptosis procedures. Thus, mitochondria act not merely as "bystanders" in this model, but by creating metabolic collapse, force cells to abandon high-energy-consuming apoptosis pathways and instead execute GSDME pyroptosis mediated by Caspase-8/3 independent of apoptosomes. This mechanism also explains why lysosomal damage-induced Caspase-8 activation becomes the sole, irreversible execution switch in this lethal cascade reaction.

Notably, while classical pyroptosis typically induces Th1/Th17-type immune responses by releasing IL-1β and IL-18, in our study model, we observed a non-canonical pyroptosis phenotype dominated by HMGB1 release. Previous studies indicate alarmins released by damaged epithelial barriers are key signals initiating type 2 immune responses [39,40]. Particularly HMGB1, which can enhance eosinophil survival and activation, and significantly exacerbate type 2 airway inflammation and airway hyperresponsiveness [41,42]. Furthermore, we must emphasize the fundamental role of the lactate environment itself in immune polarization. High concentrations of l-lactate have been proven to promote macrophage polarization towards M2 type and support Th2 cell differentiation [12]. Therefore, our data support a "two-hit" model: l-lactate metabolic reprogramming first creates a Th2-skewed permissive immune milieu, while ATP6V1B2 lactylation-mediated GSDME pyroptosis and subsequent explosive release of HMGB1 act as potent "danger signal amplifiers," pushing this basal inflammation to pathological heights of severe asthma. Indeed, existing studies corroborate that lowering HMGB1 levels through drug or probiotic intervention can synchronously inhibit Th2 cytokines and improve airway remodeling [42,43], further confirming that blocking ATP6V1B2 lactylation (AAV-2KR) significantly lowers Th2 factors precisely because it severs this critical inflammatory amplification circuit. *In vivo* AAV gene intervention experiments confirmed the pathological significance of ATP6V1B2 lactylation. Blocking modification at this site (AAV-2KR) significantly attenuated airway inflammation, IgE levels, and Th2 cytokine release. Notably, lactate levels in the lung tissue of AAV-2KR group mice were also significantly reduced. This suggests a positive feedback loop exists between inflammation and metabolism, where inflammation promotes glycolysis to produce lactate, and lactate further exacerbates inflammation through ATP6V1B2 lactylation; blocking this modification can effectively break this vicious cycle.

Finally, to bridge our molecular findings to the clinical setting, we analyzed public transcriptome data from human asthma patients. The significant upregulation of the pyroptosis execution gene GSDME (DFNA5) mRNA in severe asthma patients (P = 0.023) provides robust translational support for the involvement of the GSDME-dependent pyroptosis pathway in advanced human disease. Conversely, the expression of ATP6V1B2 mRNA remained stable across different asthma severity groups. This finding strongly corroborates our conclusion that the mechanism driving ATP6V1B2's role in asthma is its susceptibility to l-lactate-induced post-translational modification rather than changes in its mRNA transcription.

Additionally, the metabolic regulatory function of ATP6V1B2 may have broader significance. For instance, elevated ATP6V1B2 transcription levels in monocytes of COVID-19 patients correlate with impaired oxidative phosphorylation [3], suggesting this molecule might serve as a cross-disease immunometabolic hub involved in regulating various inflammatory diseases.

This study opens several key translational directions. First, validating the relevance of this signal axis in clinical samples from severe asthma patients is urgent. Second, identifying specific enzymes (writers) that catalyze ATP6V1B2 lactylation [44], and developing small molecule inhibitors targeting K108/K109 lactylation sites [45,46], will provide new drug targets for asthma treatment. Finally, deeply investigating intercellular propagation mechanisms of this "pyroptosis-DAMPs-inflammation" cycle is crucial. Notably, besides soluble DAMPs (like HMGB1), recent studies indicate extracellular vesicles (EVs) can also mediate intercellular propagation of "death signals" during cell death processes (like ferroptosis), with mitochondrial-lysosomal interactions playing key roles [47]. Future research combining dual-targeting fluorescent probes developed in this study to track organelle dynamics, and investigating whether EVs participate in the diffusion of lethal signals in asthmatic airways, will provide new perspectives for parsing spatiotemporal evolution mechanisms of airway inflammation.

Limitations of the study While our study delineates the l-lactate-ATP6V1B2-GSDME axis in asthma pathogenesis, there are limitations. First, our findings are based on murine models and human epithelial cell lines; validation in clinical samples from asthmatic patients is warranted to confirm the relevance of ATP6V1B2 lactylation in human pathology. Second, although we identified the lactylation sites, the specific lactyltransferase (writer) and delactylase (eraser) regulating ATP6V1B2 remain to be identified. Future studies will focus on characterizing these upstream enzymes to provide more precise therapeutic targets.

In summary, this study establishes ATP6V1B2 lactylation as a key mechanism linking lactate metabolic abnormalities with lysosome-mediated GSDME pyroptosis. This discovery provides a new potential target for immunometabolic treatment of asthma with severe pathology.

## Data and materials availability

The PTM-proteomics (lactylation and succinylation) dataset generated in this study is available via the ProteomeXchange Consortium (iProX partner repository) with the accession number PXD070618.

## Funding

National Natural Science Foundation of China (grant 32560211)National Natural Science Foundation of China (grant 82560010)National Natural Science Foundation of China (grant 82160329)Natural Science Research Foundation of Jilin Province for Sciences and Technology DepartmentProject (grant 20240404025 ZP)

## CRediT authorship contribution statement

**Qiaoyun Bai:** Formal analysis, Investigation, Software, Writing – original draft. **Ningpo Ding:** Data curation, Formal analysis. **Rixin Feng:** Investigation, Resources. **Fengxiang Shang:** Investigation, Methodology. **Zongqi Wang:** Data curation, Project administration. **Liangchang Li:** Funding acquisition, Supervision. **Zhiguang Wang:** Software, Visualization. **Yihua Piao:** Conceptualization, Project administration. **Guangyu Jin:** Formal analysis, Investigation. **Yilan Song:** Software, Supervision. **Guanghai Yan:** Funding acquisition, Investigation, Supervision, Visualization.

## Declaration of competing interest

The authors declare that they have no competing interests concerning the materials, methods, or findings presented in this manuscript, which is consistent with the "Competing interests" statement provided in the main text.
