# Supplementary material for: Lactate-driven ATP6V1B2 lactylation triggers asthmatic inflammation by linking lysosomal dysfunction to mitochondrial ROS-dependent pyroptosis
Source: Redox Biol. 2026 Jan 30;90:104059. doi: 10.1016/j.redox.2026.104059 (PMC12891965; doi:10.1016/j.redox.2026.104059)
Supplement: Multimedia component 1 [file mmc1.docx]

Supplementary Materials for

Lactate-driven ATP6V1B2 lactylation triggers asthmatic inflammation by linking lysosomal dysfunction to mitochondrial ROS-dependent pyroptosis

Qiaoyun Bai *et al*.

* Corresponding authors:

Guanghai Yan,

Department of Anatomy, Histology and Embryology, Yanbian University Medical College, No. 977, Gongyuan Road, Yanji 133002, P. R. China. Tel: +86-433-243-5137. Fax: +86-433-243-5136. E-mail: ghyan2015@sina.com & ghyan@ybu.edu.cn

Yilan Song,

Department of Anatomy, Histology and Embryology, Yanbian University Medical College, No. 977, Gongyuan Road, Yanji 133002, P. R. China. Tel: +86-433-243- 5135. Fax: +86-433-243-5135. E-mail: [songyl@ybu.edu.cn](mailto:songyl@ybu.edu.cn)

Guangyu Jin,

Department of Radiology, Affiliated Hospital of Yanbian University, Yanji, 133000, No.1327, Juzi Street, Jilin Province, P.R. China. Tel: 86-15526770516. Email: [kimguangyu@163.com](mailto:kimguangyu@163.com)

**
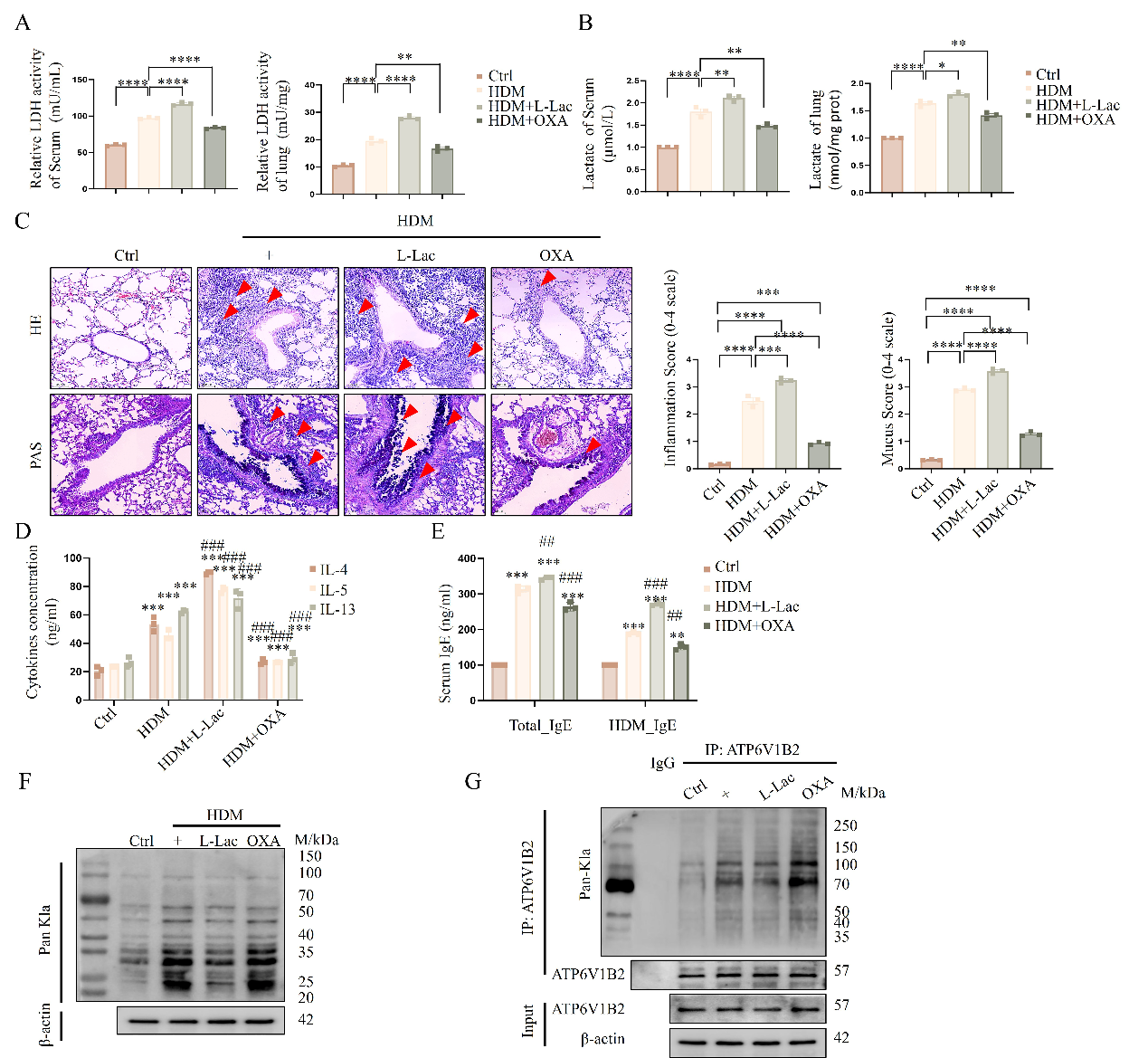
**

**Supplementary Figure S1.**

**Validation of intranasal L-lactate administration inducing airway inflammation and ATP6V1B2 lactylation.** Mice were treated with intranasal (i.n.) administration of L-lactate (1.25 mg/mouse, pH 7.0) combined with HDM challenge. (A, B) Quantitative analysis of LDH activity and L-lactate concentrations in lung tissue homogenates and serum. (C) Representative H&E and PAS staining of lung tissue sections (scale bar: 50 µm). Histological scores for inflammation and mucus secretion are shown on the right. (D) ELISA detection of Th2 cytokines (IL-4, IL-5, IL-13) in BALF. (E) ELISA detection of serum total IgE levels. (F, G) Immunoprecipitation (IP) analysis of ATP6V1B2 lactylation in lung tissues. Lysates were immunoprecipitated with anti-ATP6V1B2 antibody, followed by immunoblot analysis with anti-Pan-Kla antibody. Data are expressed as Mean ± SEM (n=10 mice per group). *P < 0.05, **P < 0.01, ***P < 0.001, ****P < 0.0001 (One-way ANOVA).


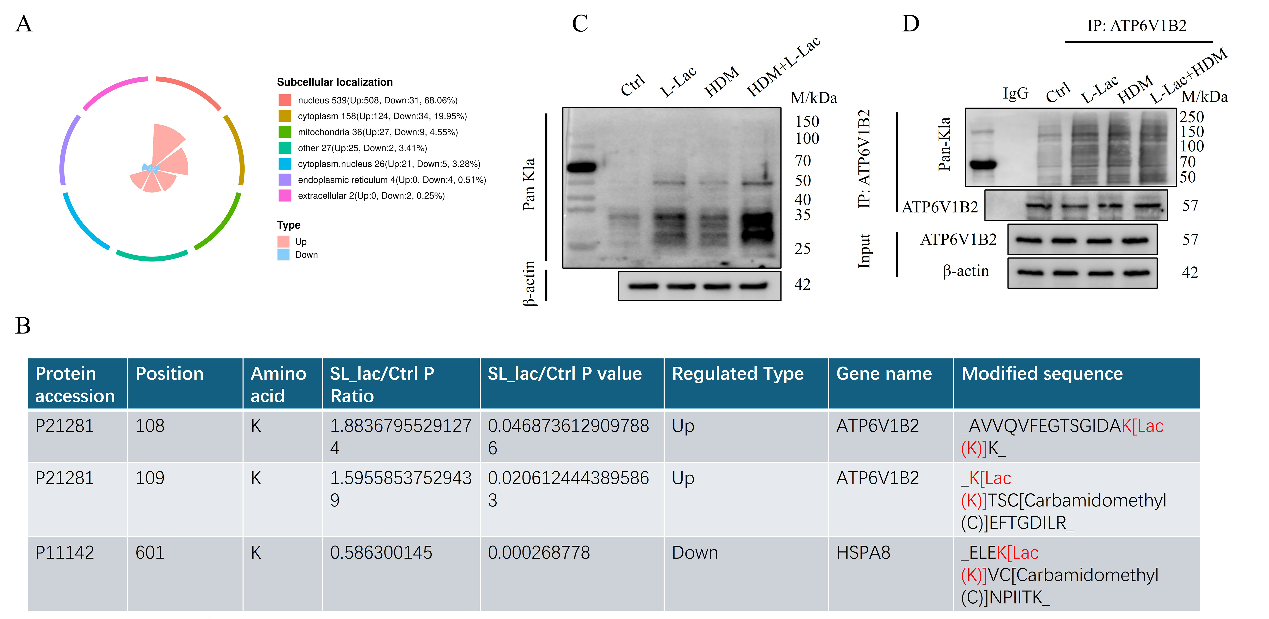


**Supplementary Figure S2.**

**Subcellular distribution of lactylated proteins and validation in primary human bronchial epithelial cells (HBEs).** (A) Subcellular localization chart of differentially lactylated proteins identified by 4D-FastDIA quantitative proteomics. (B) Detailed mass spectrometry information for the identified lactylation sites (K108, K109) on ATP6V1B2 and the site (K601) on HSPA8, showing fold change ratios and P values. (C) Immunoblot analysis of Pan-Kla levels in primary human bronchial epithelial cells (HBEpiC) treated with Control, HDM, L-Lac, or HDM+L-Lac. (D) Validation of ATP6V1B2 lactylation in primary HBEpiC. Cell lysates were immunoprecipitated with anti-ATP6V1B2 antibody, and lactylation levels were detected using anti-Pan-Kla antibody. Data are representative of three independent experiments.


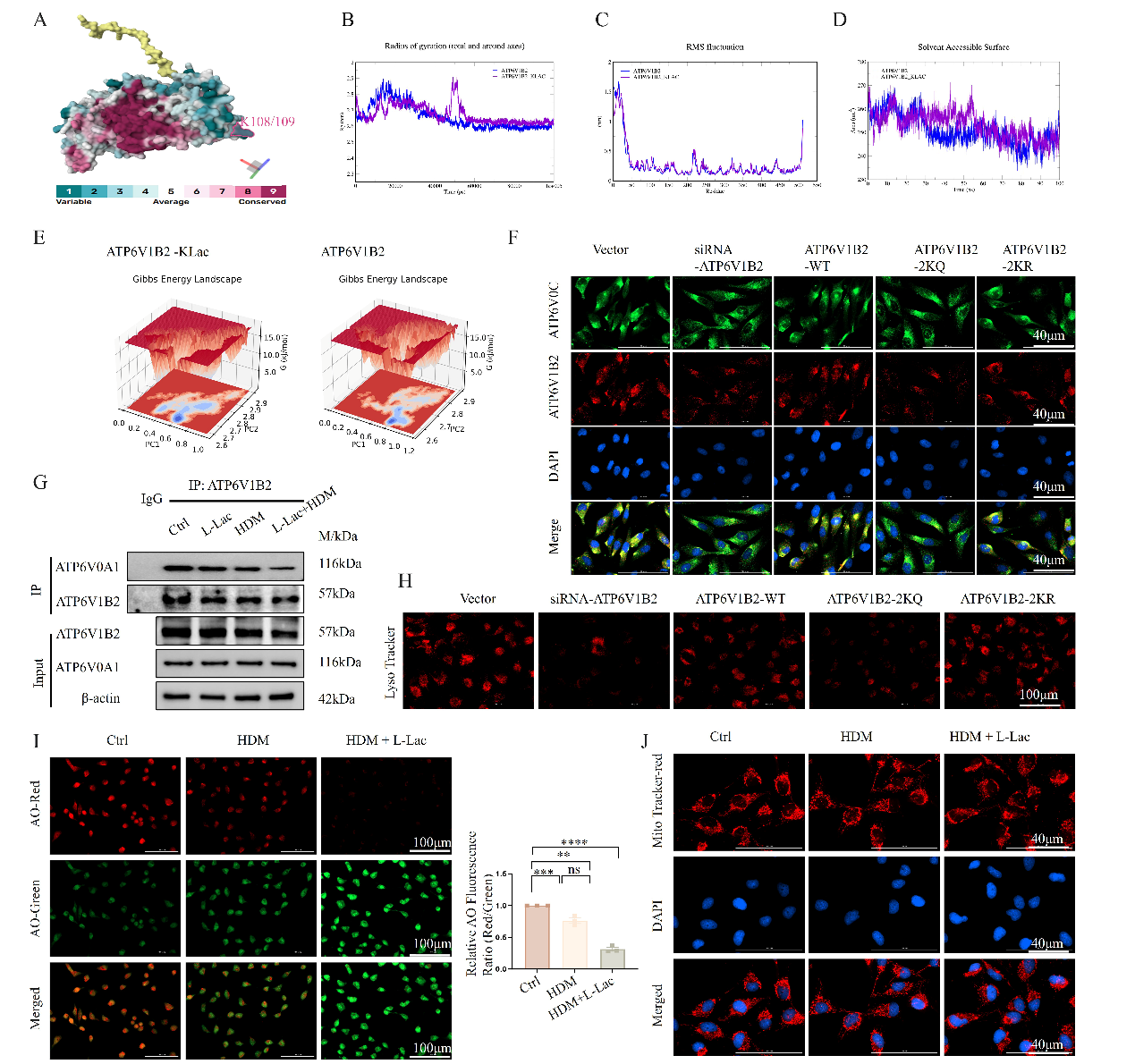


**Supplementary Figure S3.**

**Structural analysis of ATP6V1B2 lactylation and its impact on V-ATPase assembly and acidification function.** (A) Structural representation of ATP6V1B2 showing the surface exposure of K108/K109 residues. (B-E) Molecular dynamics (MD) simulation analysis (100 ns) comparing Wild-Type (WT) and Lactylated (KLac) ATP6V1B2 models: (B) Radius of Gyration (Rg), (C) Root-Mean-Square Fluctuation (RMSF), (D) Solvent Accessible Surface Area (SASA), and (E) Gibbs Free Energy Landscape (FEL). (F) Immunofluorescence imaging analysis of V1-V0 domain assembly. 2KR or 2KQ cells were co-stained for Flag (V1 subunit, green) and ATP6V0A1 (V0 subunit, red). (G) Endogenous Co-IP assay in BEAS-2B cells. Interaction between endogenous ATP6V1B2 (V1) and ATP6V0A1 (V0) was assessed under Control, L-Lac, HDM, and HDM+L-Lac conditions. (H) V-ATPase proton-pumping activity assay. Lysosomal acidification (LysoTracker MFI) was compared among 2KR, 2KQ, and ATP6V1B2 knockdown (siRNA) groups. (I) Representative images and quantitative analysis of Acridine Orange (AO) staining comparing the independent and synergistic effects of HDM and L-Lac on lysosomal pH. (J) Analysis of mitochondrial fragmentation under HDM vs. HDM+L-Lac conditions using MitoTracker Red. Data are expressed as Mean ± SEM (n=3). **P < 0.01, ***P < 0.001, ****P < 0.0001; ns, not significant.


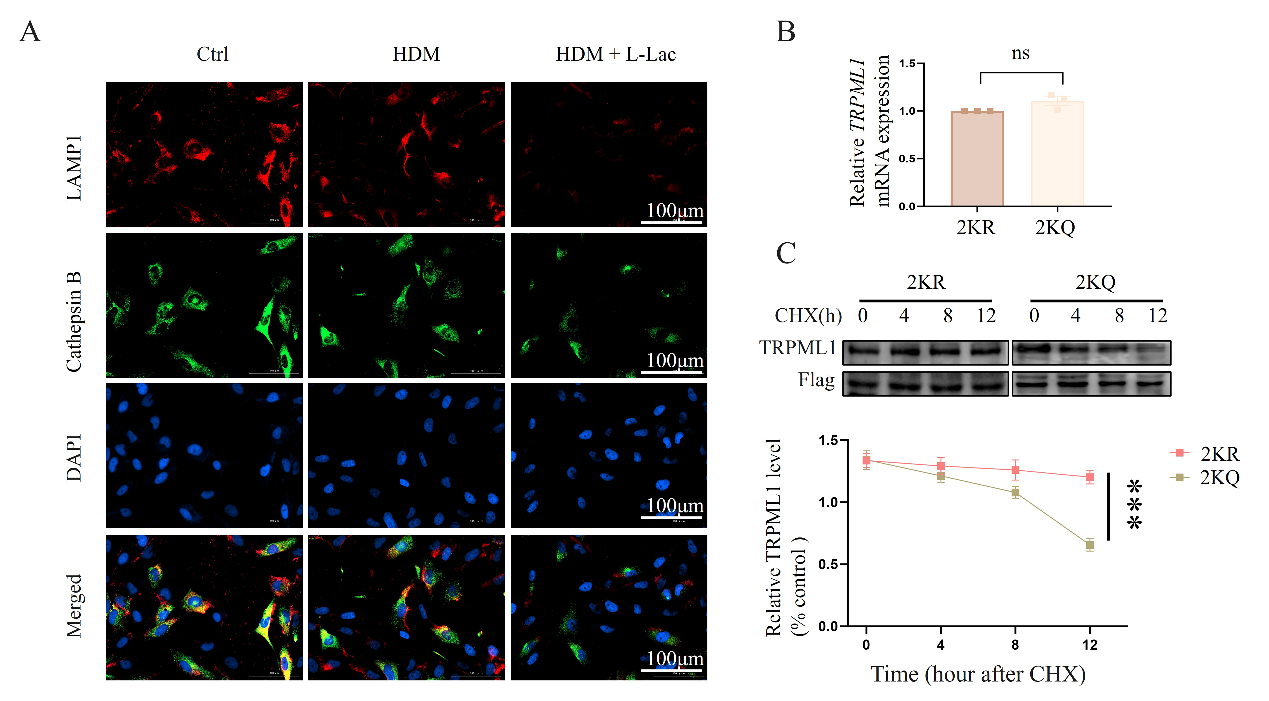


**Supplementary Figure S4.**

**Synergistic effect of L-lactate on lysosomal membrane permeabilization (LMP) and mechanism of TRPML1 downregulation.** (A) Immunofluorescence analysis of Cathepsin B leakage (LMP). BEAS-2B cells were treated with Control, L-Lac, HDM, or HDM+L-Lac. Cytosolic diffusion of Cathepsin B (green) indicates LMP. (B) RT-qPCR analysis of TRPML1 mRNA expression levels in BEAS-2B cells expressing 2KR or 2KQ mutants. (C) Cycloheximide (CHX) chase assay. Cells were treated with CHX (100 µg/mL) for the indicated times (0, 4, 8, 12 h). TRPML1 protein degradation rates were analyzed by Western Blot. Data are expressed as Mean ± SEM (n=3). ***P < 0.001; ns, not significant.

**
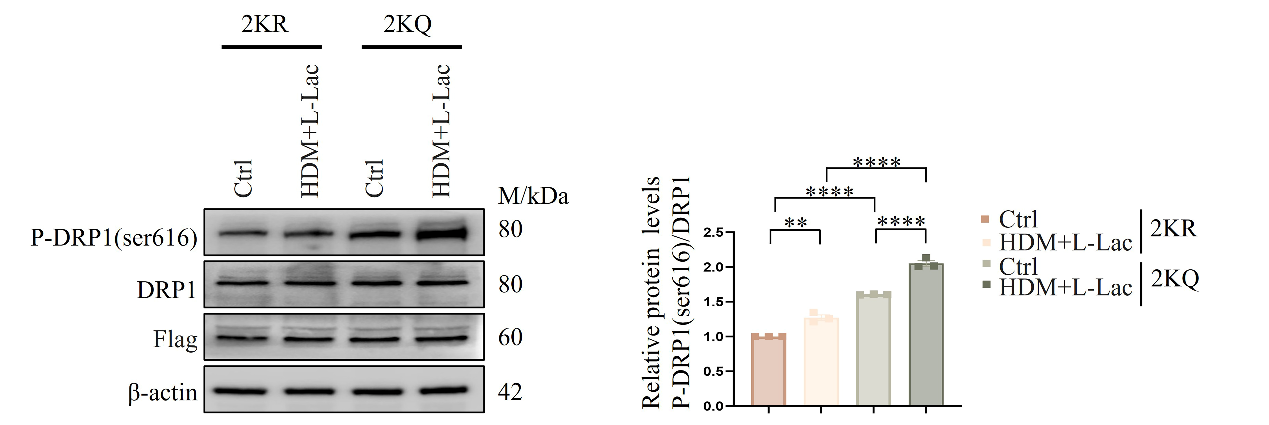
**

**Supplementary Figure S5.**

**ATP6V1B2 lactylation promotes DRP1 activation via phosphorylation at Ser616.** Immunoblot analysis of total DRP1 and phosphorylated DRP1 (Ser616) in BEAS-2B cells expressing 2KR or 2KQ mutants under L-Lac+HDM stimulation. The ratio of p-DRP1(Ser616)/Total DRP1 was quantified to assess the activation of mitochondrial fission. Data are expressed as Mean ± SEM (n=3). **P < 0.01, ****P < 0.0001.


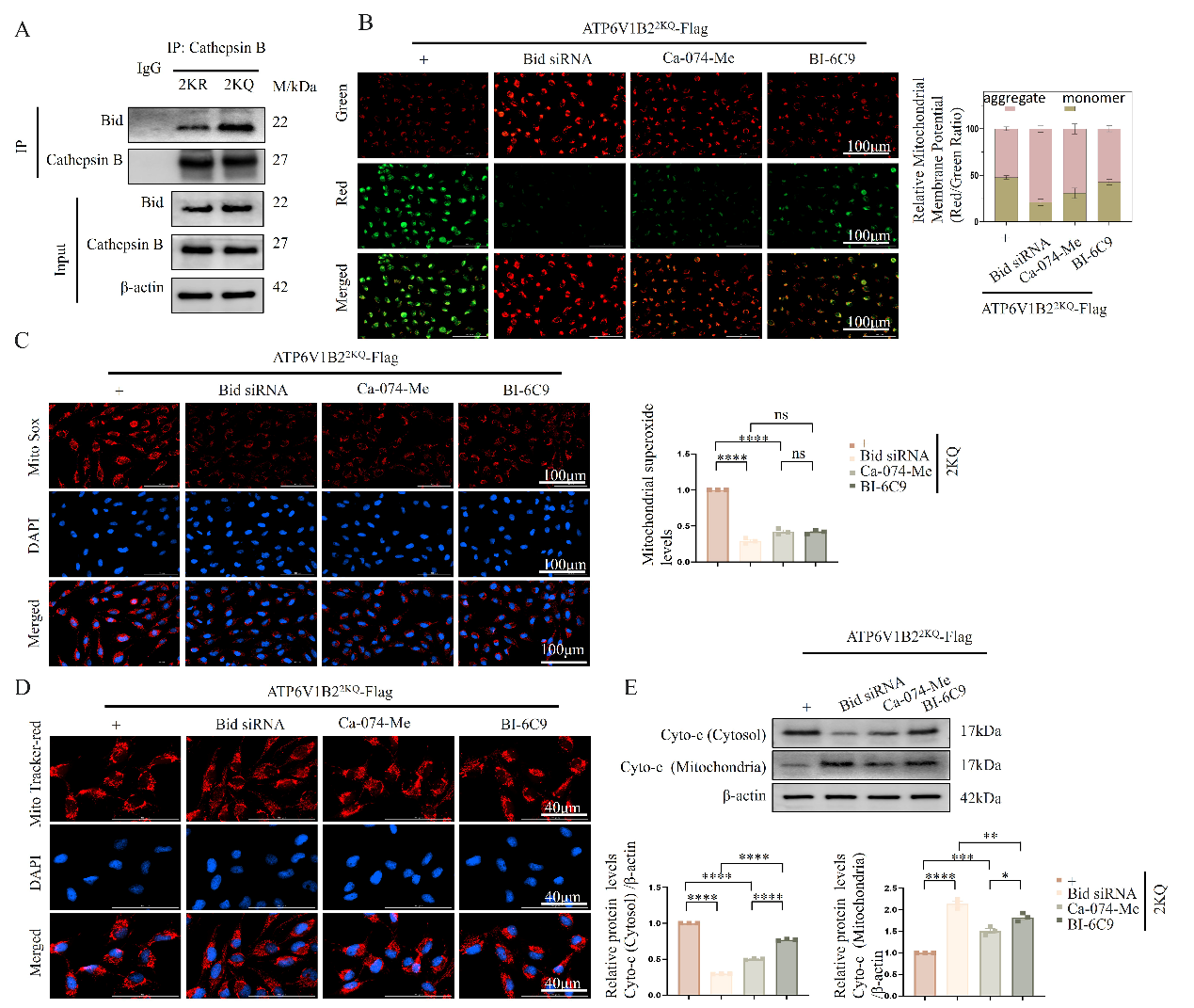


**Supplementary Figure S6.**

**Mechanism of Cathepsin B-mediated Bid cleavage and mitochondrial damage.** (A) Co-immunoprecipitation (Co-IP) analysis of the interaction between endogenous Cathepsin B and Bid in 2KR and 2KQ cells. (B-E) Evaluation of the tBid-mitochondrial axis using Bid siRNA (Si Bid) and Bid inhibitor (BI-6C9). (B) Mitochondrial membrane potential (JC-1). (C) Mitochondrial superoxide levels (MitoSOX Red). (D) Mitochondrial morphology (MitoTracker Red). (E) Release of Cytochrome c from mitochondria to cytosol (Western Blot). Data are expressed as Mean ± SEM (n=3). *P < 0.05, **P < 0.01, ***P < 0.001, ****P < 0.0001.

**
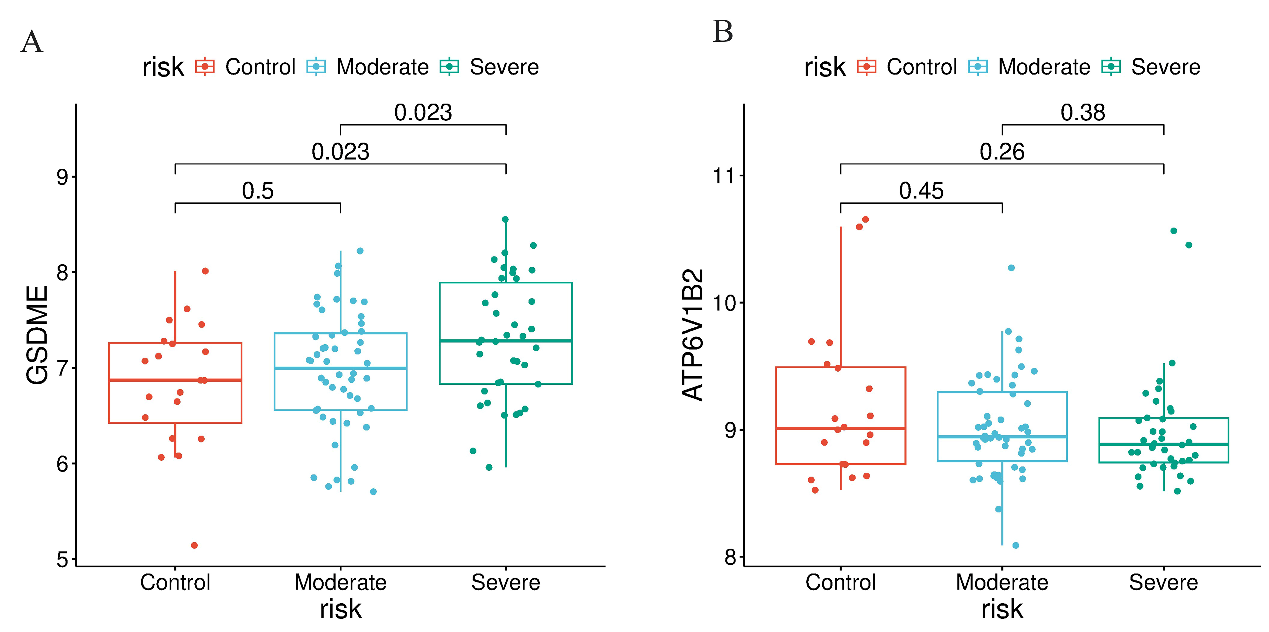
**

**Supplementary Figure S7.**

**Clinical relevance analysis of GSDME and ATP6V1B2 in human asthma transcriptomes.** Analysis of the public microarray dataset GSE43696 (bronchial epithelial cells from asthma patients). (A) Expression levels of GSDME (DFNA5) mRNA in Healthy Control, Moderate Asthma, and Severe Asthma groups. (B) Expression levels of ATP6V1B2 mRNA in the same groups. Data are presented as box plots. P values were calculated using Student's t-test.

**
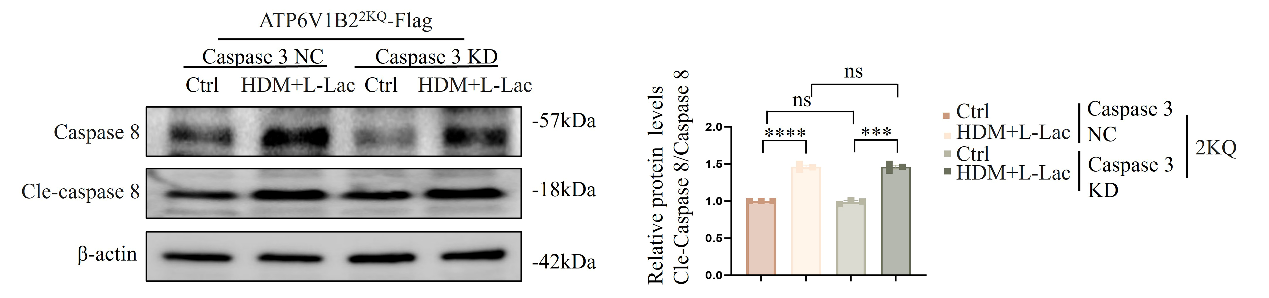
**

**Supplementary Figure S8.**

**Validation of the hierarchical relationship between Caspase-8 and Caspase-3.** To confirm that Caspase-8 is upstream of Caspase-3, BEAS-2B cells expressing the 2KQ mutant were transfected with Caspase-3 siRNA (Caspase 3 KD). Western Blot analysis showing the levels of Cleaved-Caspase-8. Data are expressed as Mean ± SEM (n=3). ***P < 0.001, ****P < 0.0001; ns, not significant.
