# Supplementary material for: Lactate-driven ATP6V1B2 lactylation triggers asthmatic inflammation by linking lysosomal dysfunction to mitochondrial ROS-dependent pyroptosis
Source: Redox Biol. 2026 Jan 30;90:104059. doi: 10.1016/j.redox.2026.104059 (PMC12891965; doi:10.1016/j.redox.2026.104059)
Supplement: Multimedia component 2 [file mmc2.pdf]

Figure 2

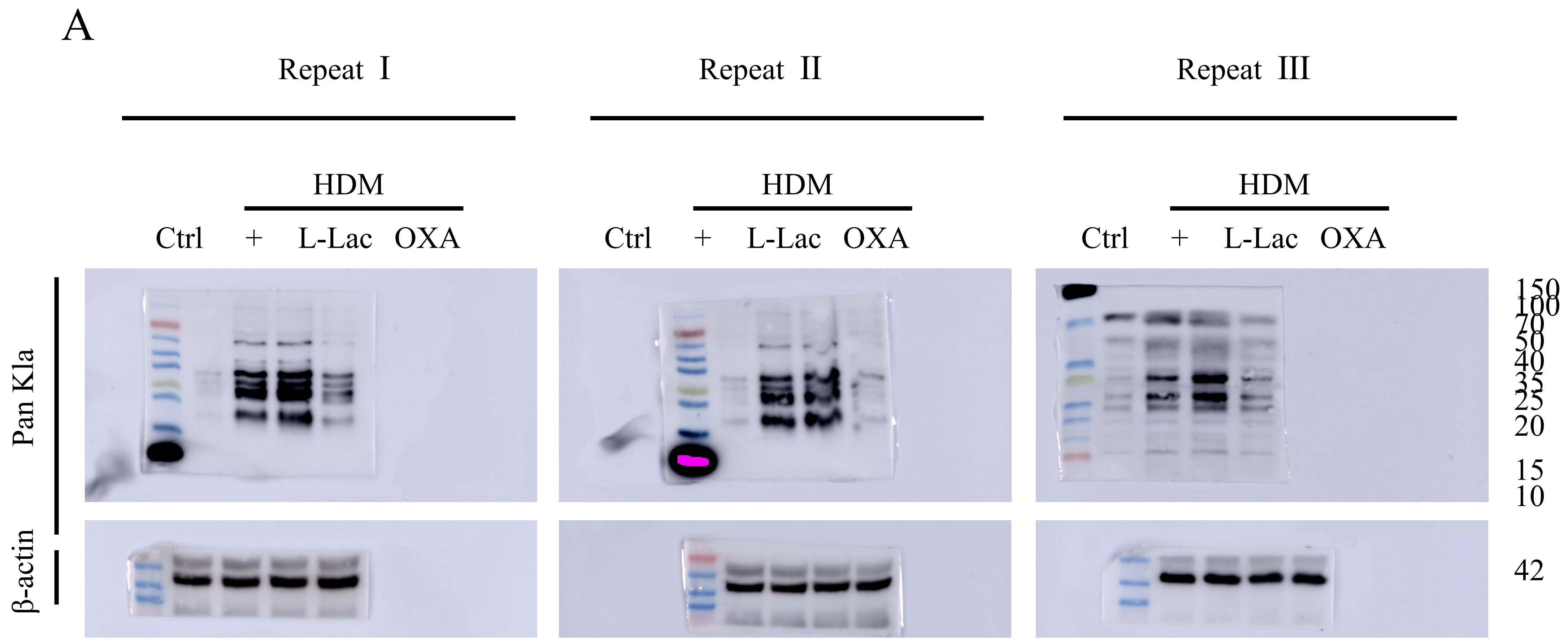
A
Repeat I
Repeat II
Repeat III
HDM
HDM
HDM
Ctrl + L-Lac OXA
Ctrl + L-Lac OXA
Ctrl + L-Lac OXA
Pan Kla
β-actin
150
100
70
50
40
35
25
20
15
10
42

Figure 2

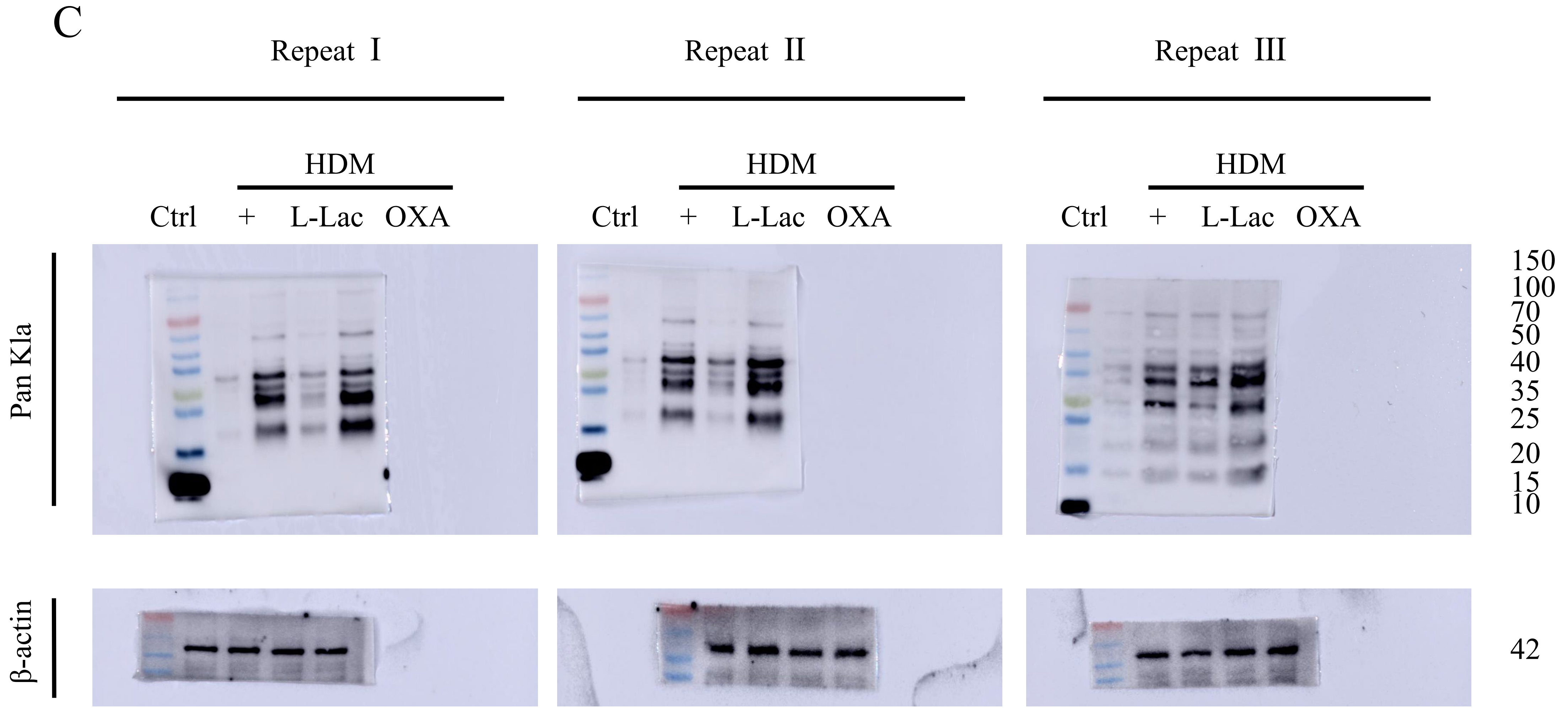
C
Repeat I
Repeat II
Repeat III
HDM
Ctrl
+
L-Lac
OXA
Pan Kla
β-actin
150
100
70
50
40
35
25
20
15
10
42

Figure 2

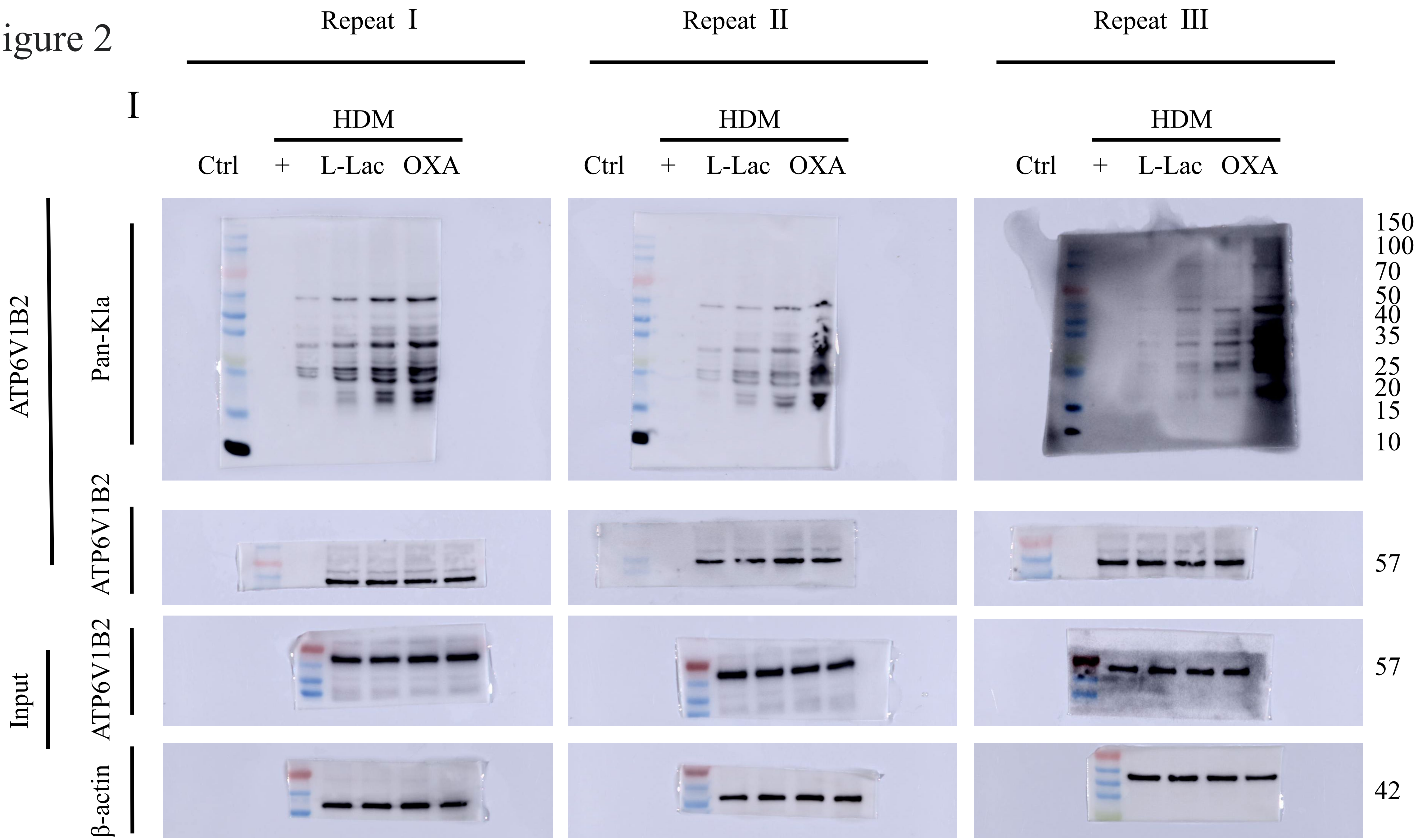
Repeat Ⅰ
Repeat Ⅱ
Repeat Ⅲ
I
HDM
Ctrl
+
L-Lac
OXA
ATP6V1B2
Pan-Kla
ATP6V1B2
Input
ATP6V1B2
β-actin
150
100
70
50
40
35
25
20
15
10
57
57
42

Figure 3

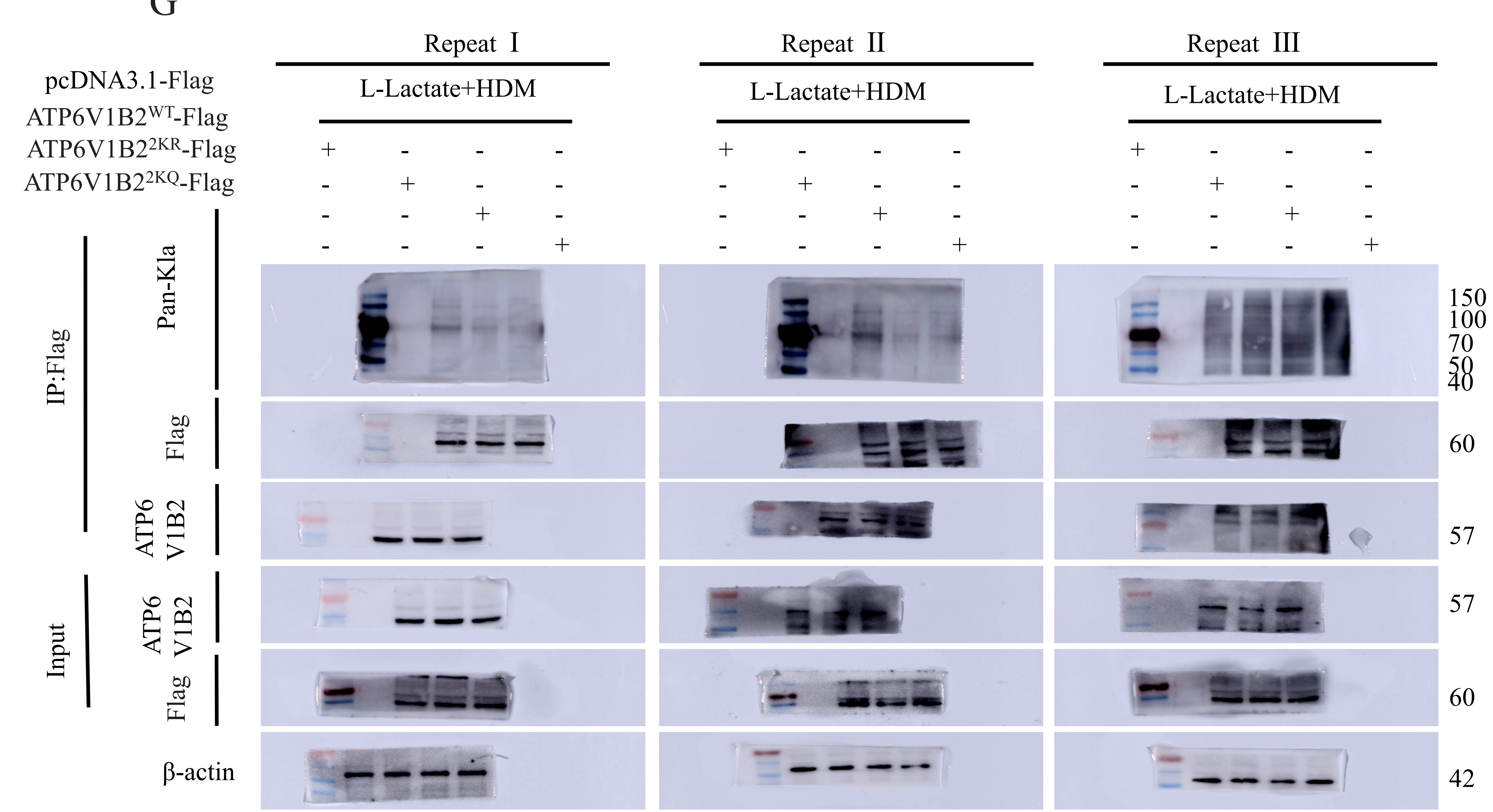
G
Repeat I
Repeat II
Repeat III
L-Lactate+HDM
L-Lactate+HDM
L-Lactate+HDM
pcDNA3.1-Flag
ATP6V1B2WT-Flag
ATP6V1B22KR-Flag
ATP6V1B22KQ-Flag
IP:Flag
Input
Pan-Kla
Flag
ATP6 V1B2
ATP6 V1B2
Flag
β-actin
150
100
70
50
40
60
57
57
60
42

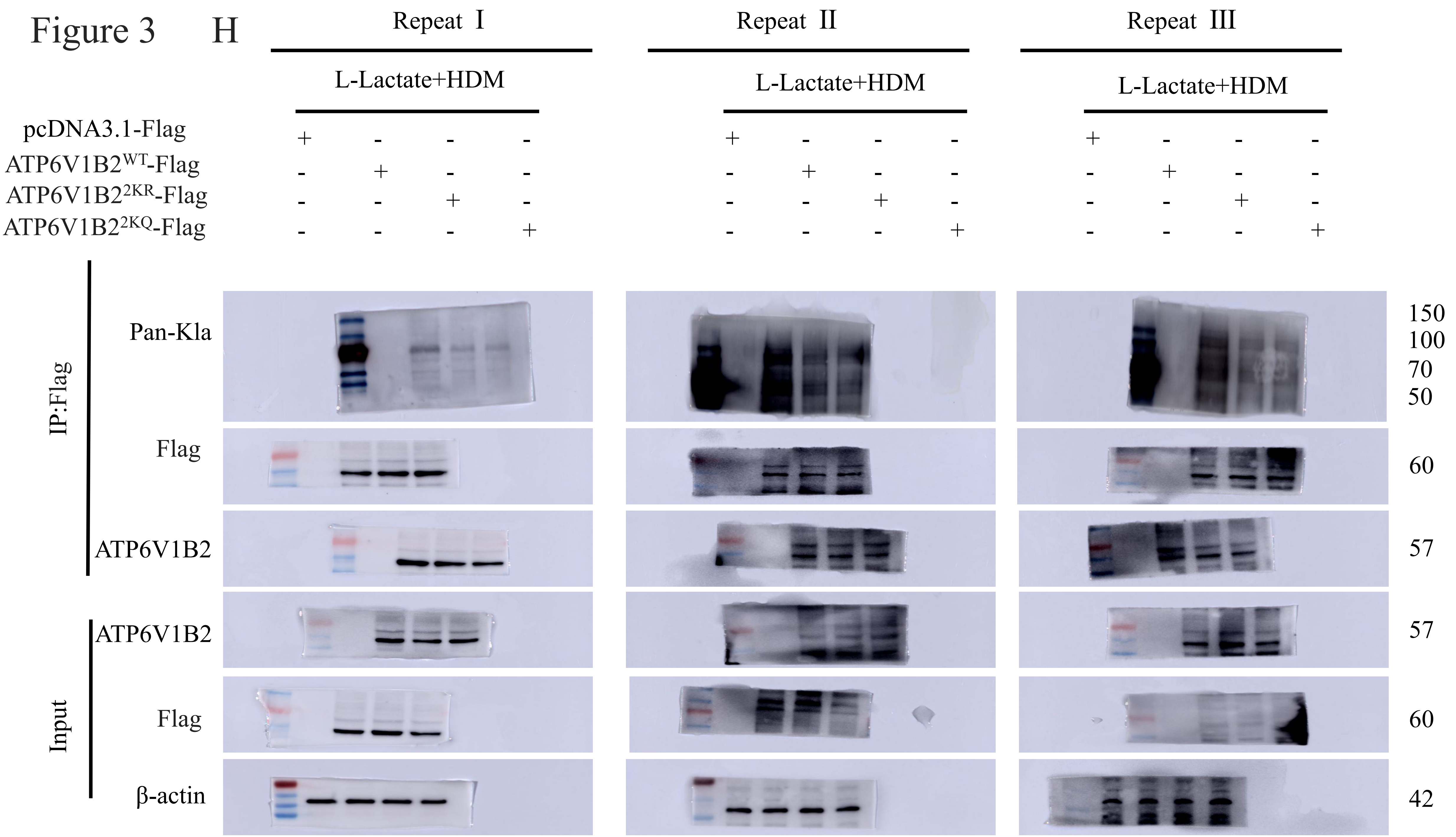
Figure 3
H
Repeat Ⅰ
Repeat Ⅱ
Repeat Ⅲ
L-Lactate+HDM
L-Lactate+HDM
L-Lactate+HDM
pcDNA3.1-Flag
ATP6V1B2WT-Flag
ATP6V1B22KR-Flag
ATP6V1B22KQ-Flag
IP:Flag
Pan-Kla
Flag
ATP6V1B2
Input
ATP6V1B2
Flag
β-actin
150
100
70
50
60
57
57
60
42

Figure 3

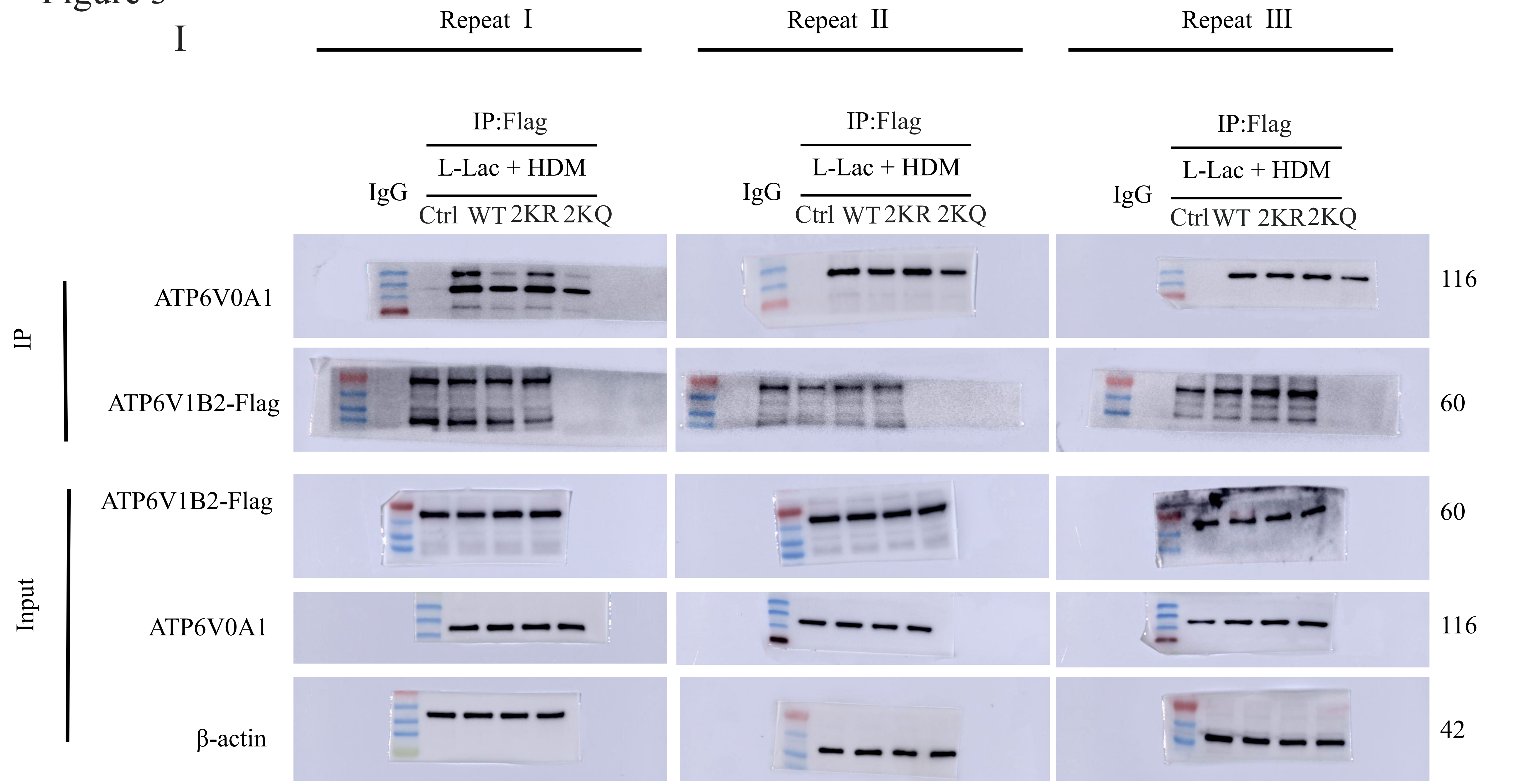
I
Repeat I
Repeat II
Repeat III
IP:Flag
L-Lac + HDM
IgG
Ctrl WT 2KR 2KQ
IP
Input
ATP6V0A1
ATP6V1B2-Flag
ATP6V1B2-Flag
ATP6V0A1
β-actin
116
60
60
116
42

Figure 4

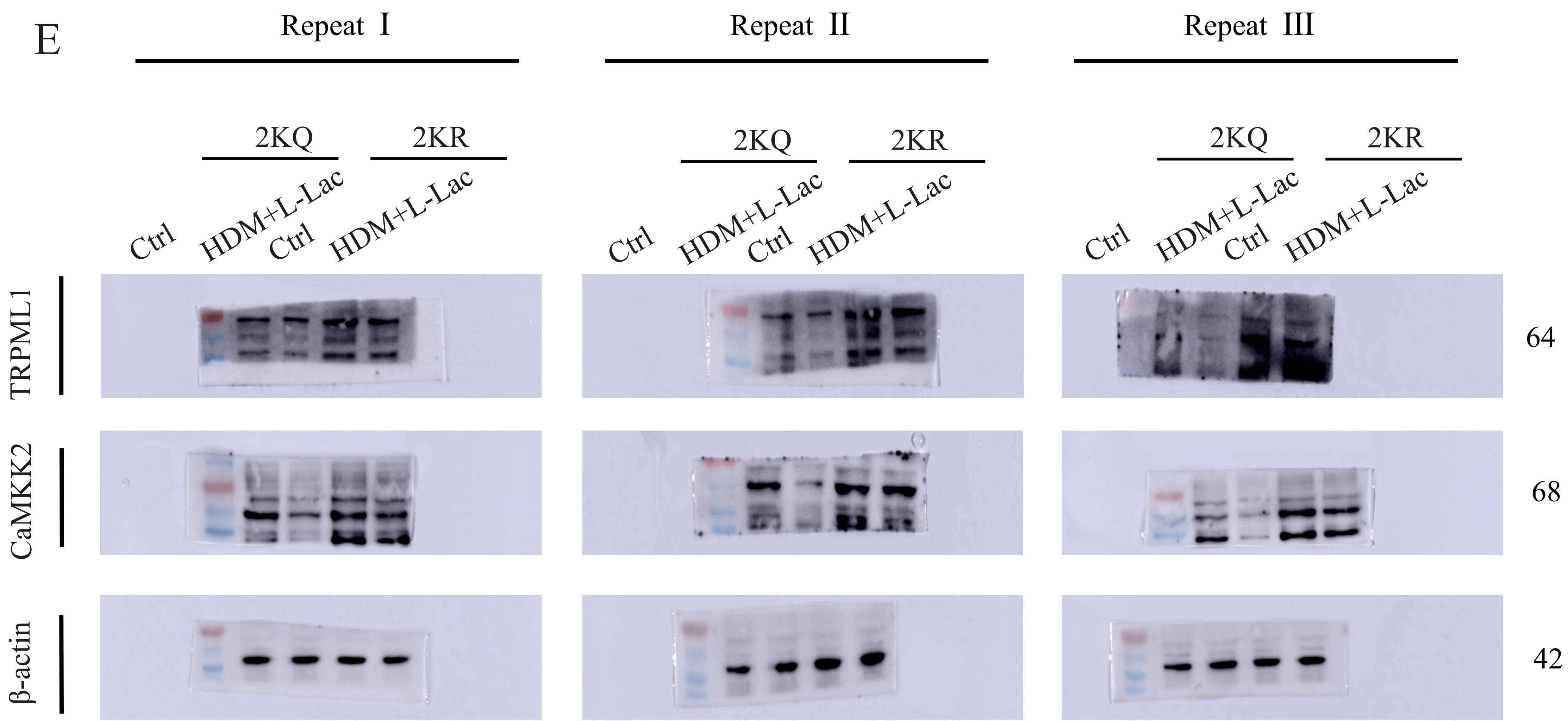
E
Repeat I
Repeat II
Repeat III
2KQ
2KR
Ctrl
HDM+L-Lac
Ctrl
HDM+L-Lac
TRPML1
CaMKK2
β-actin
64
68
42

Figure 6

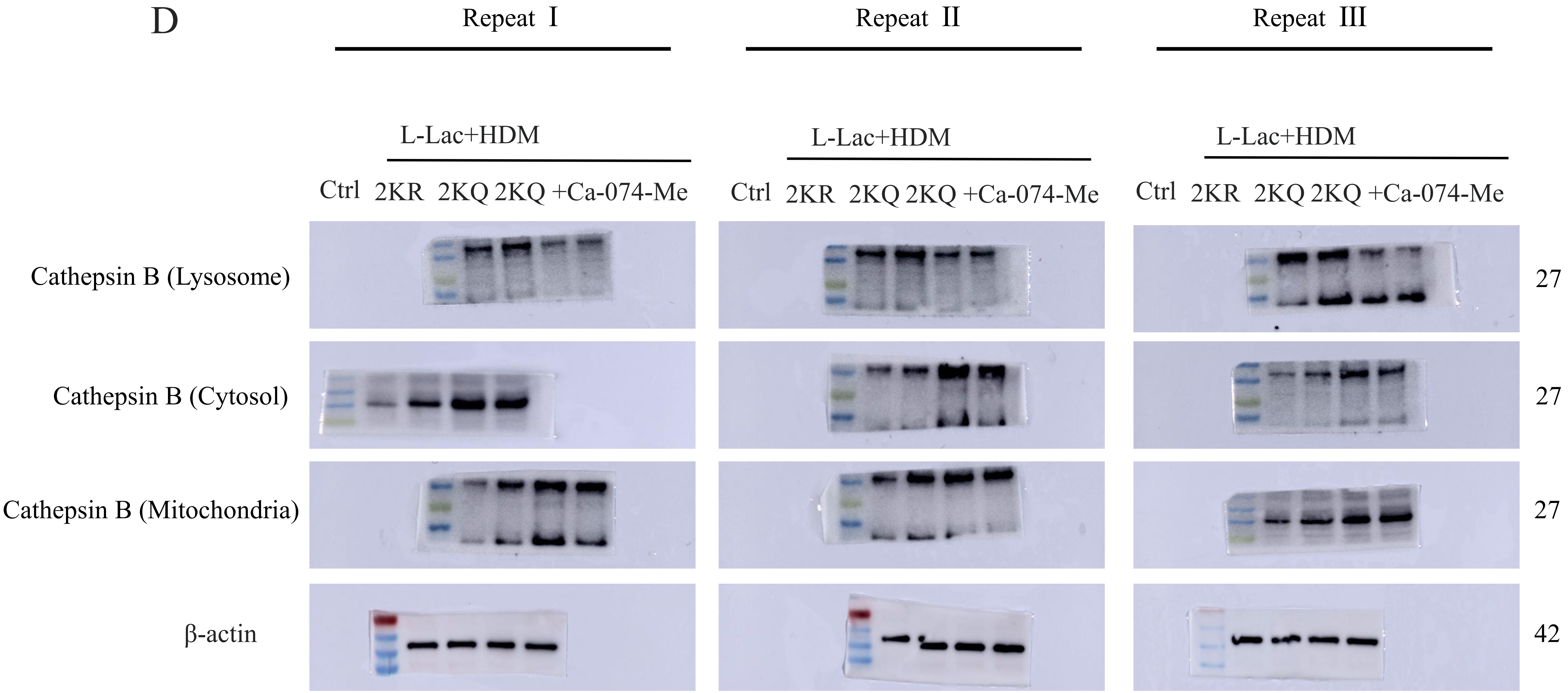

D
Repeat I
Repeat II
Repeat III
L-Lac+HDM
L-Lac+HDM
L-Lac+HDM
Ctrl 2KR 2KQ 2KQ +Ca-074-Me
Ctrl 2KR 2KQ 2KQ +Ca-074-Me
Ctrl 2KR 2KQ 2KQ +Ca-074-Me
Cathepsin B (Lysosome)
Cathepsin B (Cytosol)
Cathepsin B (Mitochondria)
β-actin
27
27
27
42

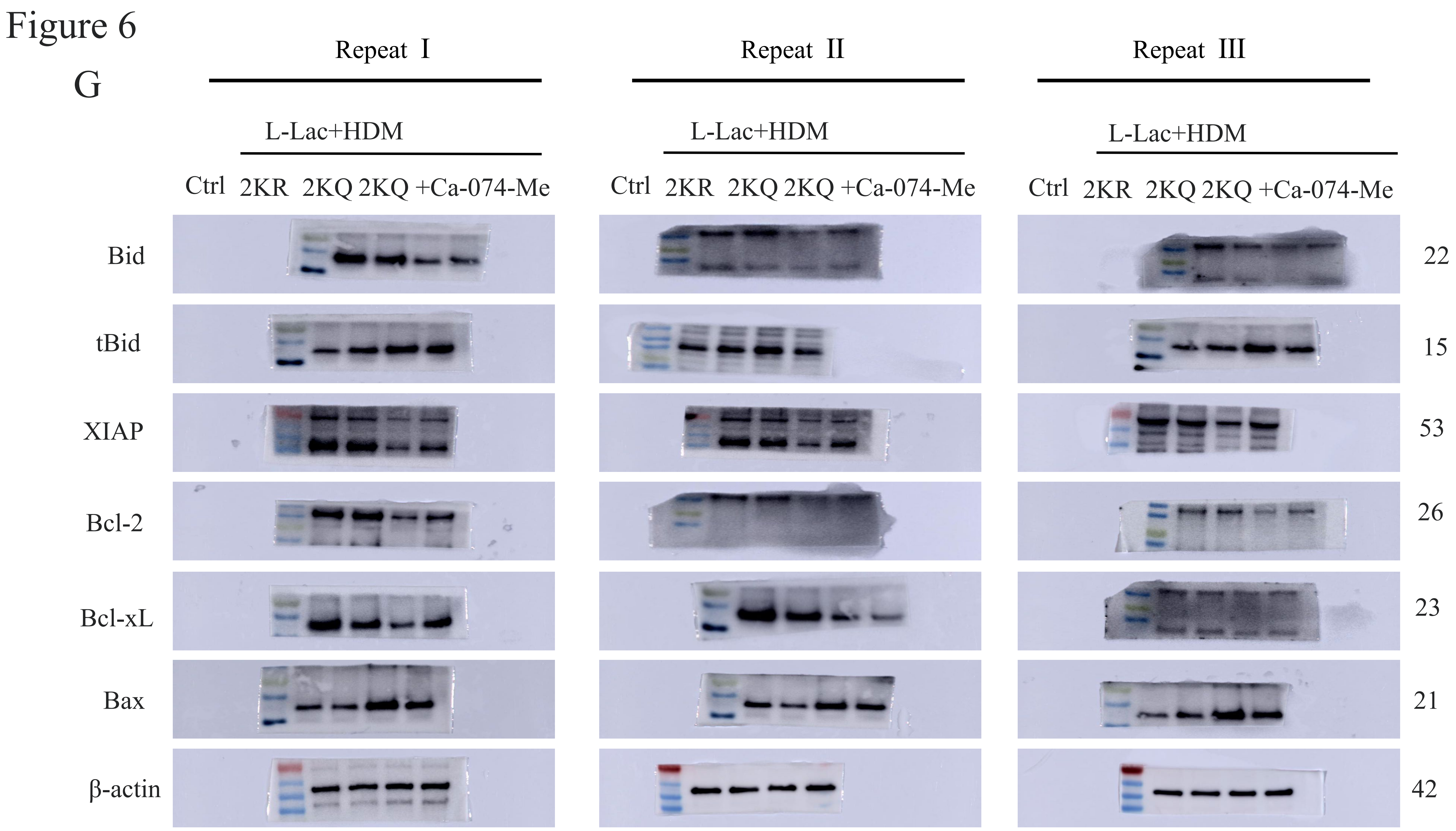
Figure 6
G
Repeat Ⅰ
Repeat Ⅱ
Repeat Ⅲ
L-Lac+HDM
L-Lac+HDM
L-Lac+HDM
Ctrl 2KR 2KQ 2KQ +Ca-074-Me
Ctrl 2KR 2KQ 2KQ +Ca-074-Me
Ctrl 2KR 2KQ 2KQ +Ca-074-Me
Bid
tBid
XIAP
Bcl-2
Bcl-xL
Bax
β-actin
22
15
53
26
23
21
42

Figure 8

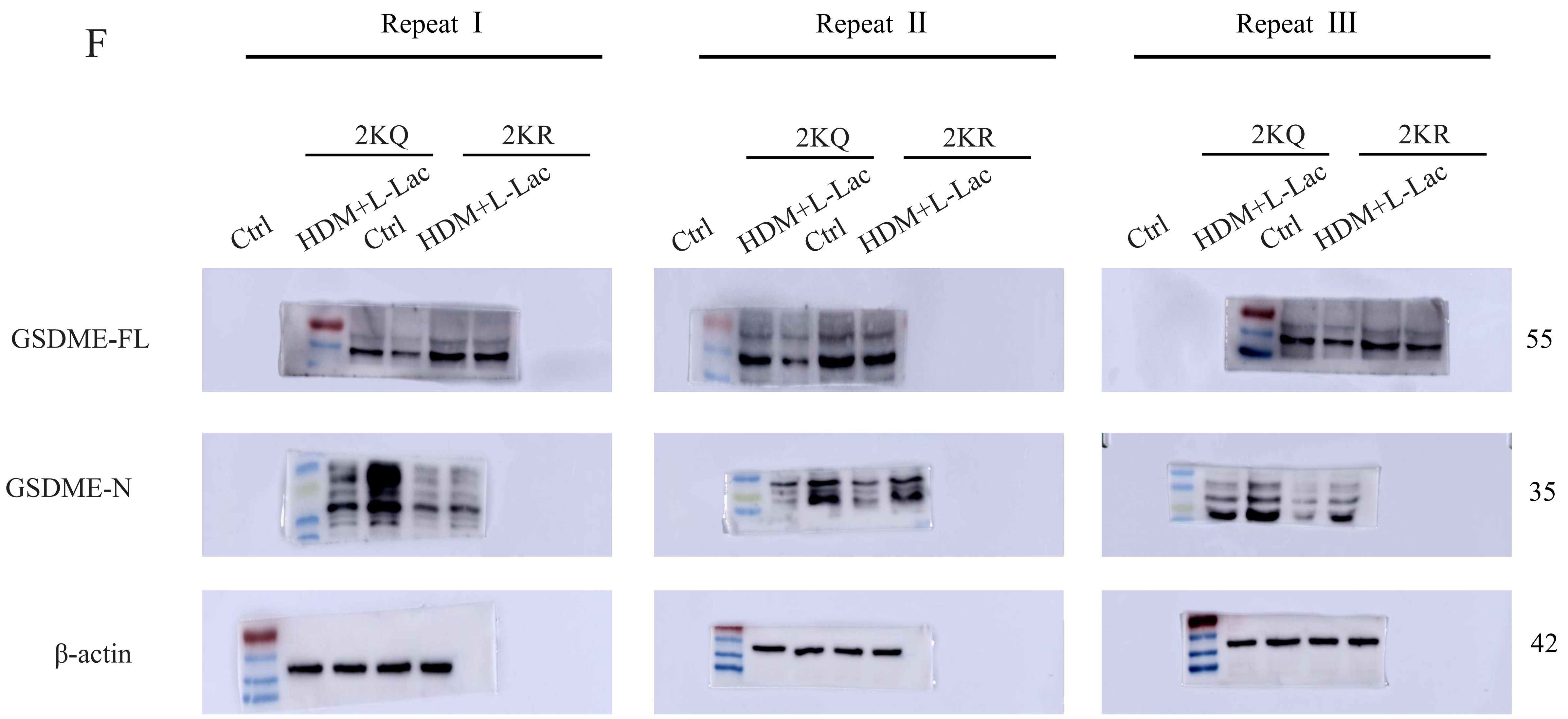
F
Repeat I
Repeat II
Repeat III
2KQ
2KR
Ctrl
HDM+L-Lac
GSDME-FL
GSDME-N
β-actin
55
35
42

Figure 8

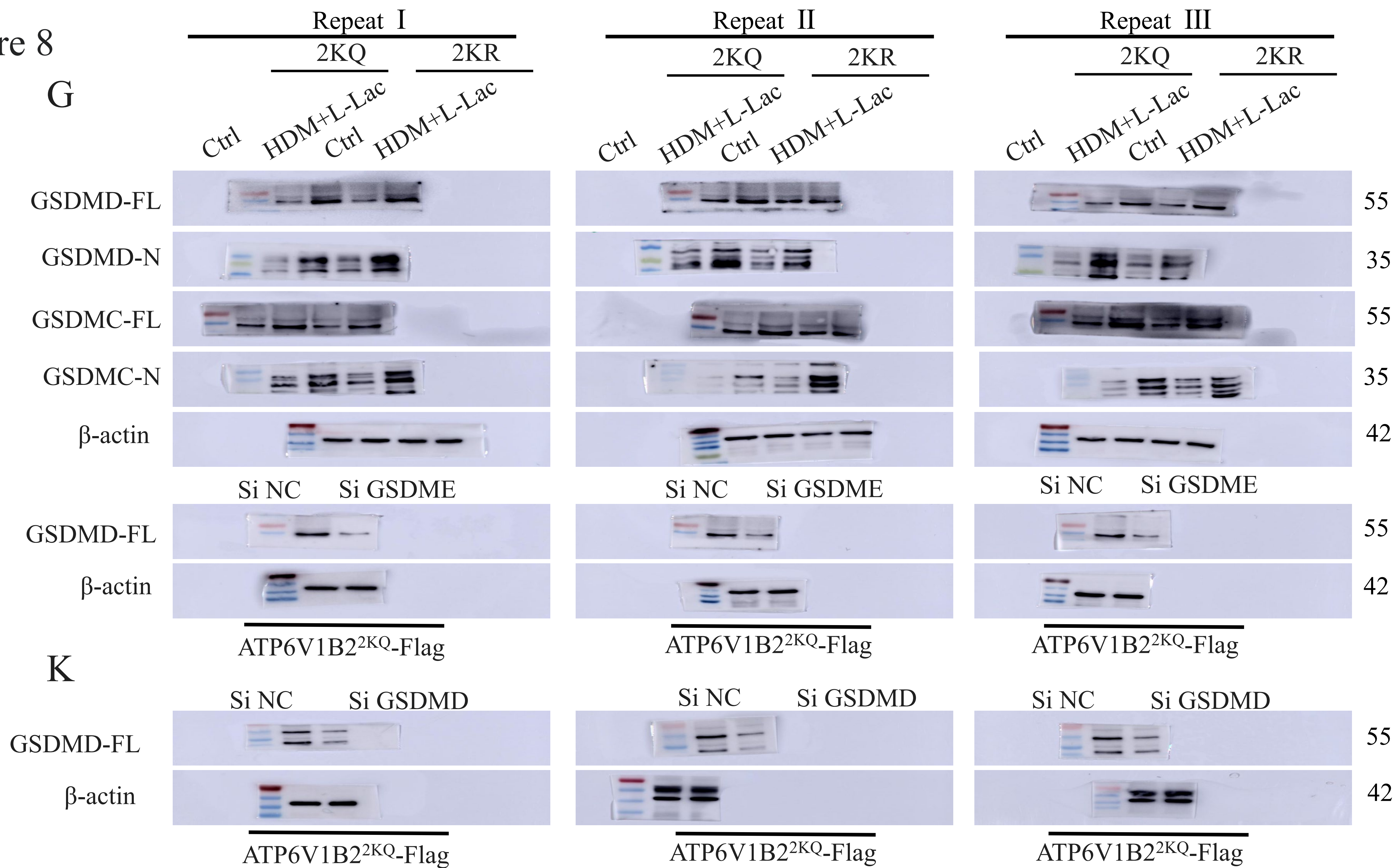
G
Repeat I
Repeat II
Repeat III
2KQ
2KR
Ctrl
HDM+L-Lac
GSDMD-FL
GSDMD-N
GSDMC-FL
GSDMC-N
β-actin
55
35
55
35
42
Si NC
Si GSDME
GSDMD-FL
β-actin
55
42
ATP6V1B2^2KQ-Flag
K
Si NC
Si GSDMD
GSDMD-FL
β-actin
55
42
ATP6V1B2^2KQ-Flag

Figure 9

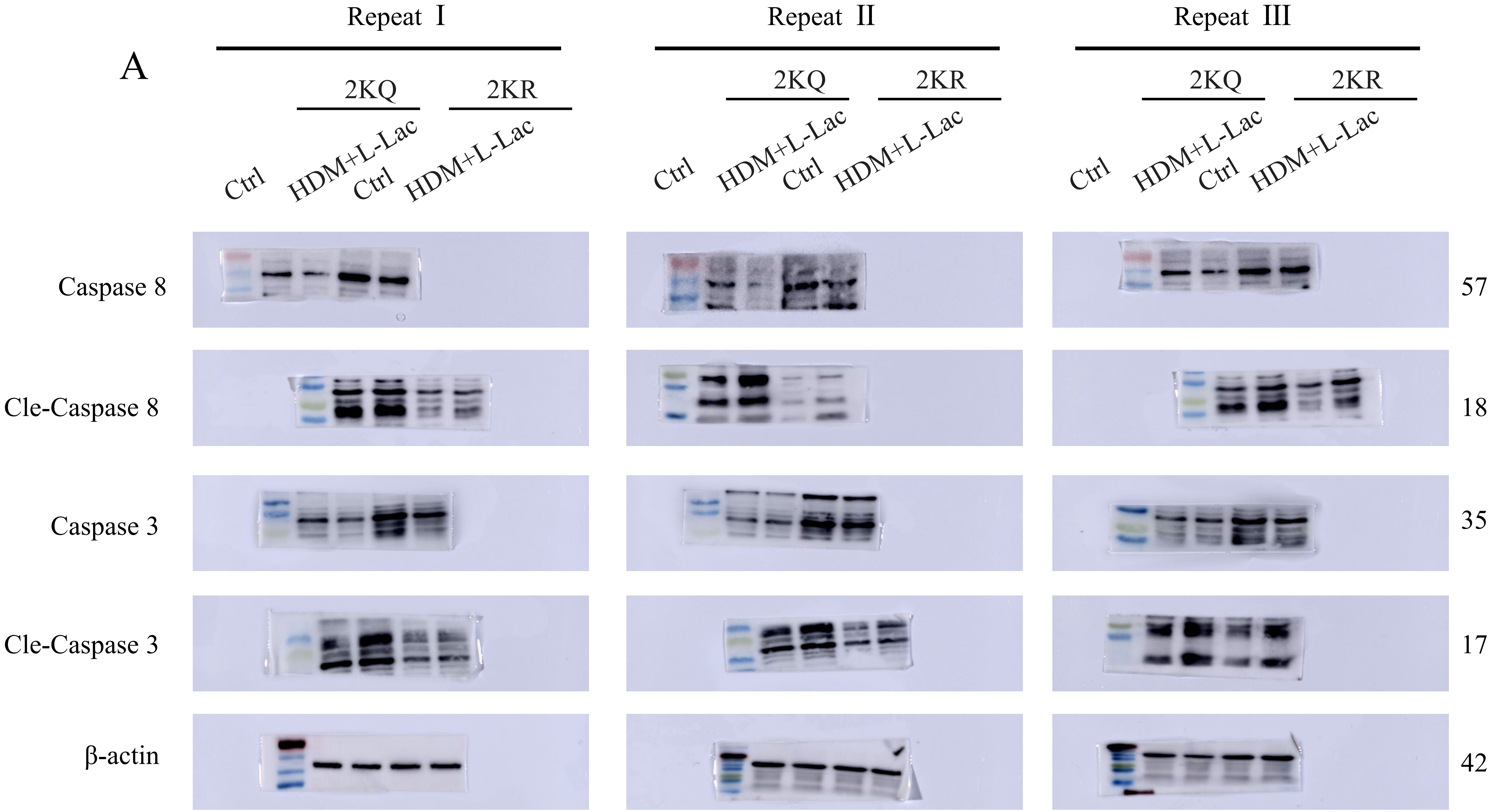
A
Repeat Ⅰ
Repeat Ⅱ
Repeat Ⅲ
2KQ
2KR
Ctrl
HDM+L-Lac
Ctrl
HDM+L-Lac
Caspase 8
Cle-Caspase 8
Caspase 3
Cle-Caspase 3
β-actin
57
18
35
17
42

Figure 9

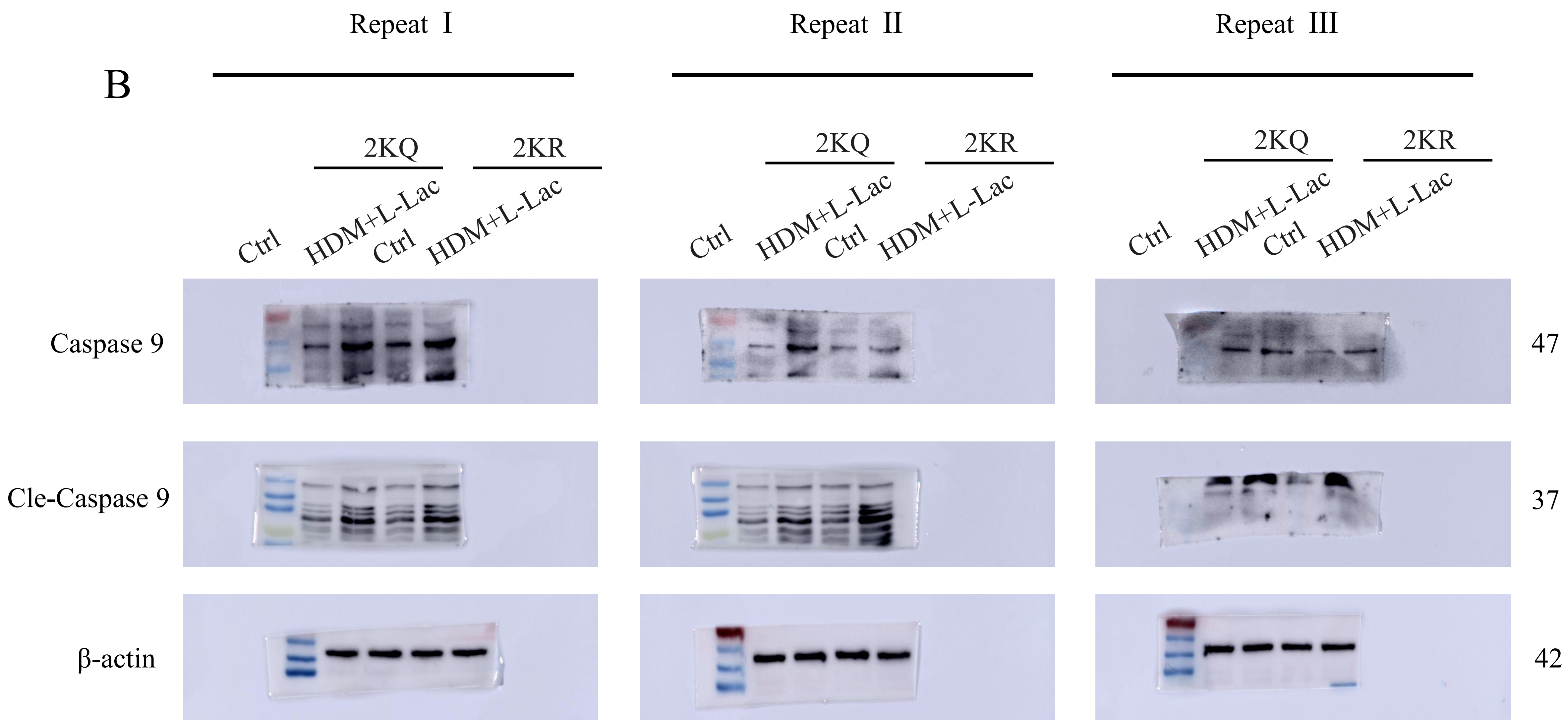
B
Repeat Ⅰ
Repeat Ⅱ
Repeat Ⅲ
2KQ
2KR
Ctrl
HDM+L-Lac
Ctrl
HDM+L-Lac
Caspase 9
Cle-Caspase 9
β-actin
47
37
42

Figure 9

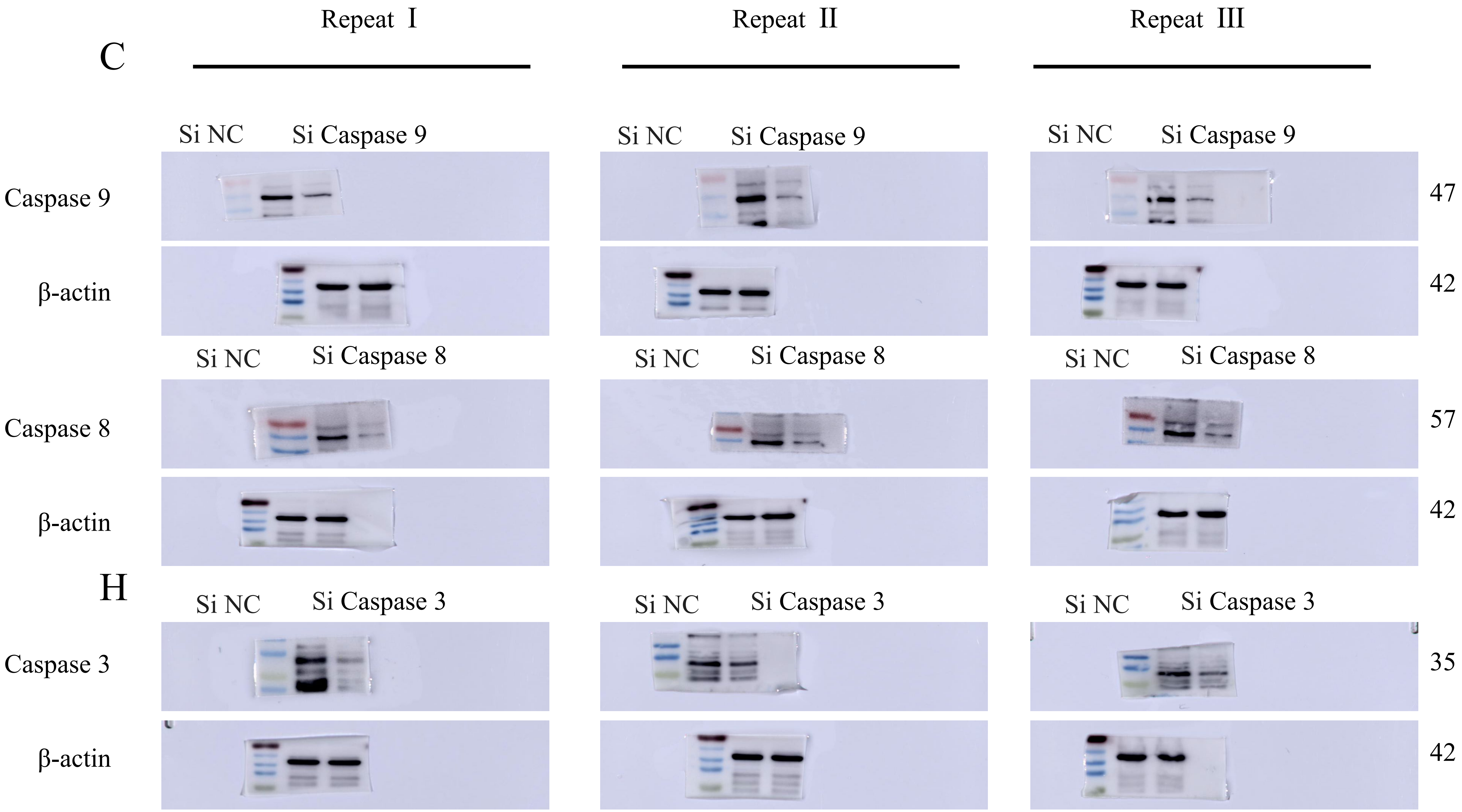
C
Repeat Ⅰ
Repeat Ⅱ
Repeat Ⅲ
Si NC
Si Caspase 9
Caspase 9
β-actin
Si NC
Si Caspase 8
Caspase 8
β-actin
H
Si NC
Si Caspase 3
Caspase 3
β-actin
47
42
57
42
35
42

Figure 9

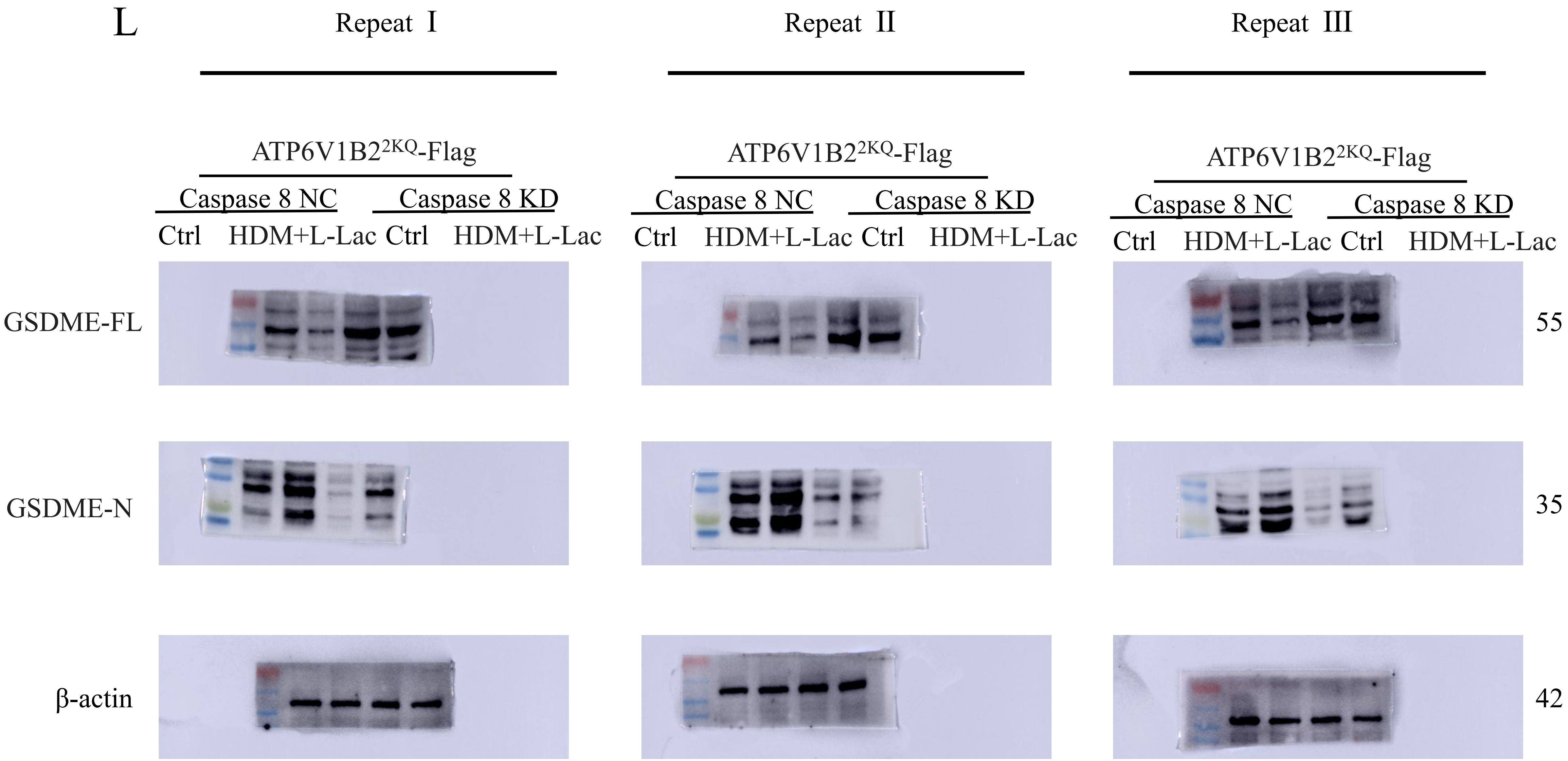
L
Repeat I
Repeat II
Repeat III
ATP6V1B2 2KQ-Flag
Caspase 8 NC
Caspase 8 KD
Ctrl
HDM+L-Lac
Ctrl
HDM+L-Lac
GSDME-FL
GSDME-N
β-actin
55
35
42

Figure 9

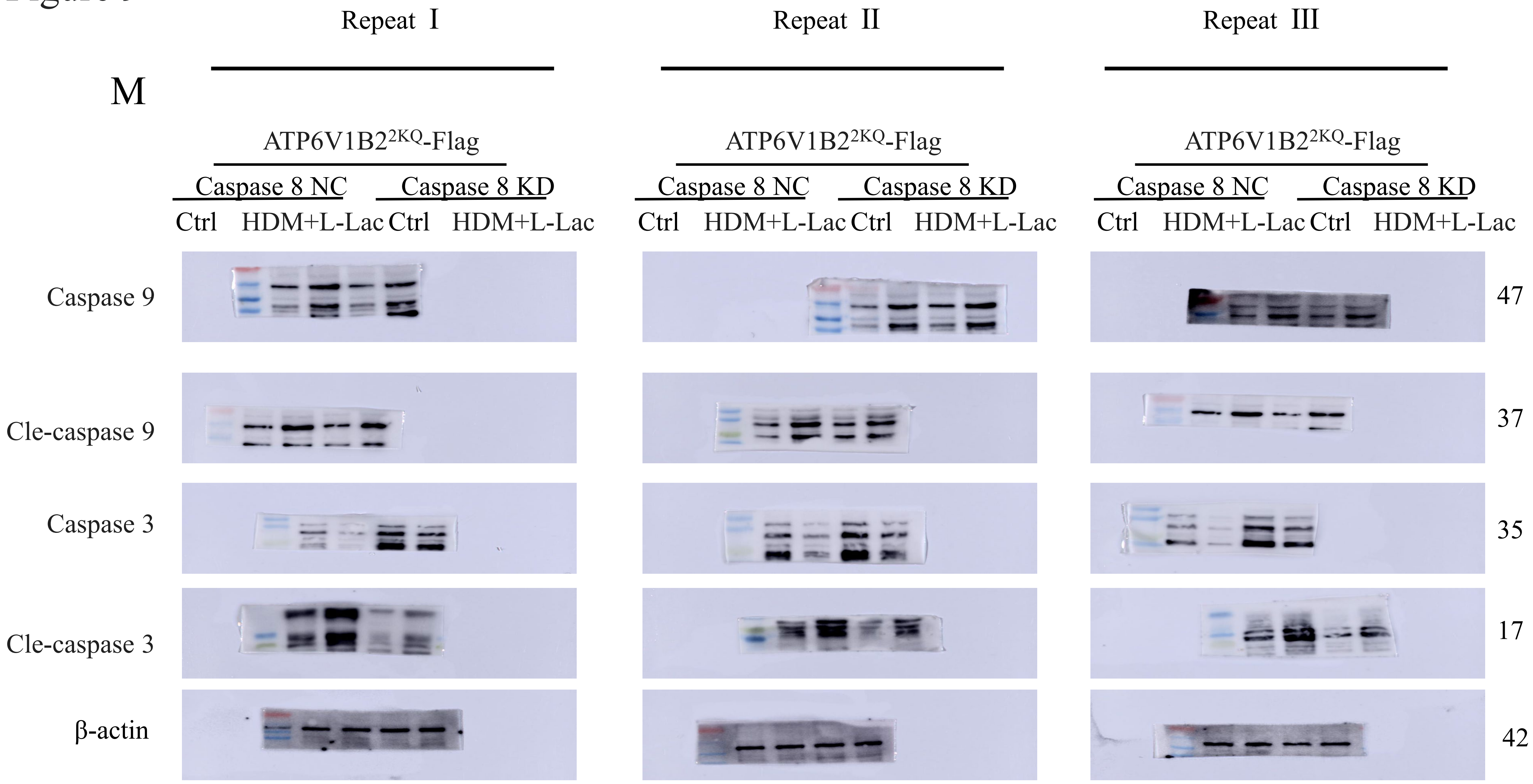
Repeat I
Repeat II
Repeat III
M
ATP6V1B2^2KQ-Flag
Caspase 8 NC
Caspase 8 KD
Ctrl
HDM+L-Lac
Caspase 9
Cle-caspase 9
Caspase 3
Cle-caspase 3
β-actin
47
37
35
17
42

Figure 10

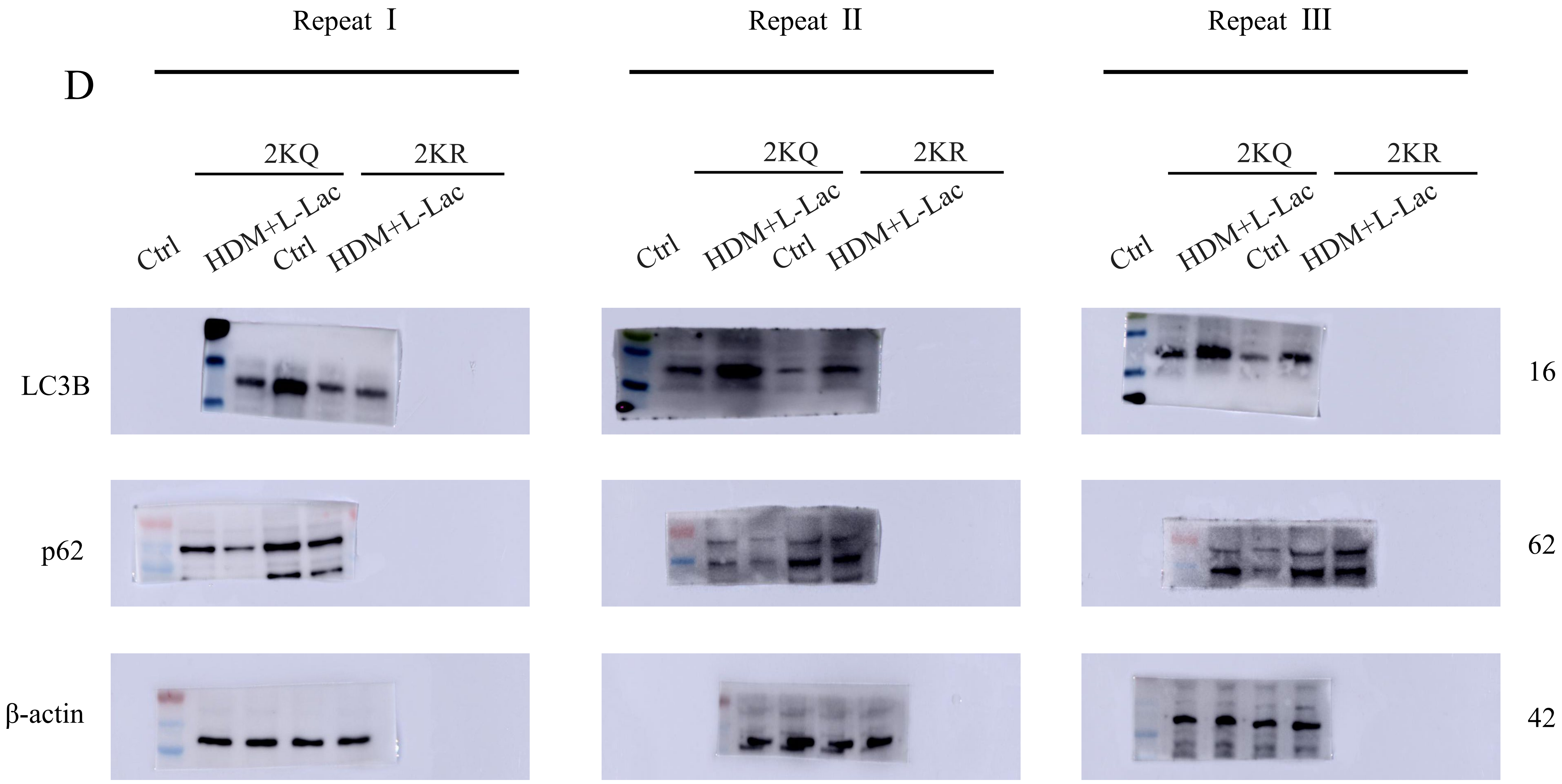
Repeat Ⅰ
Repeat Ⅱ
Repeat Ⅲ
D
2KQ
2KR
Ctrl
HDM+L-Lac
Ctrl
HDM+L-Lac
LC3B
p62
β-actin
16
62
42

Figure 10

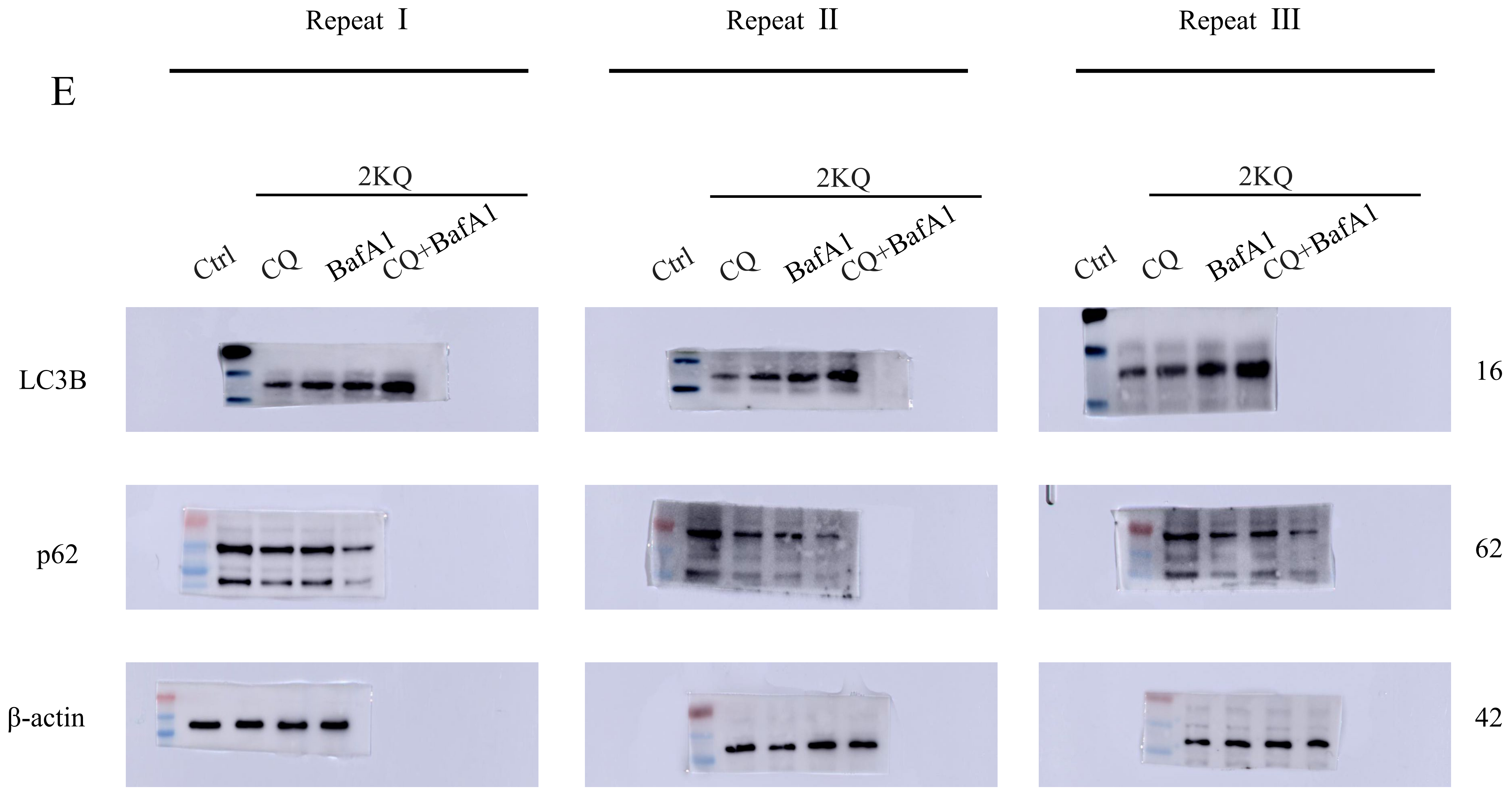
E
Repeat Ⅰ
Repeat Ⅱ
Repeat Ⅲ
2KQ
2KQ
2KQ
Ctrl
CQ
BafA1
CQ+BafA1
Ctrl
CQ
BafA1
CQ+BafA1
Ctrl
CQ
BafA1
CQ+BafA1
LC3B
p62
β-actin
16
62
42

Figure 10

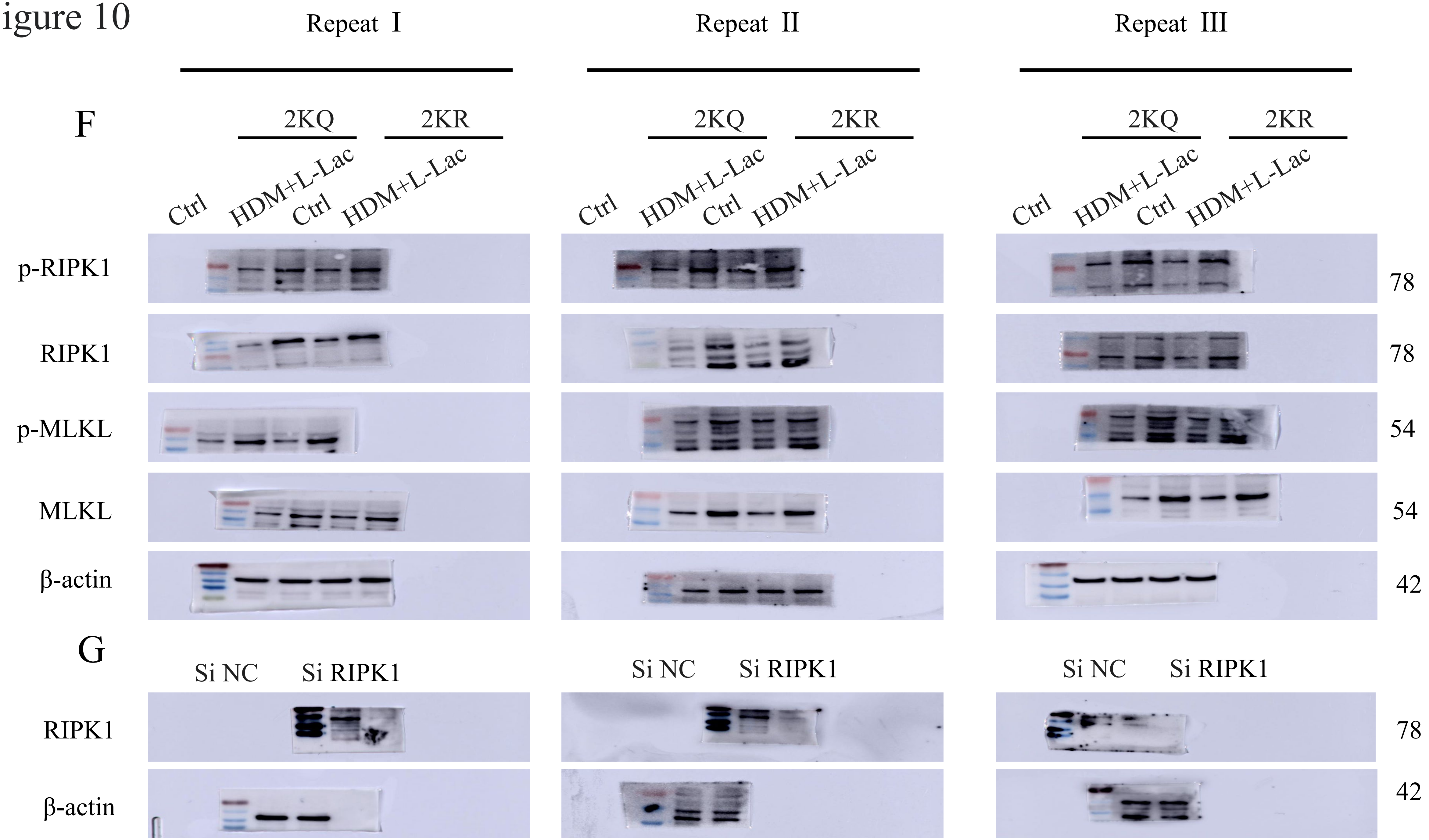
Repeat I
Repeat II
Repeat III
F
2KQ
2KR
Ctrl
HDM+L-Lac
Ctrl
HDM+L-Lac
p-RIPK1
RIPK1
p-MLKL
MLKL
β-actin
78
78
54
54
42
G
Si NC
Si RIPK1
RIPK1
β-actin
78
42

Figure 11

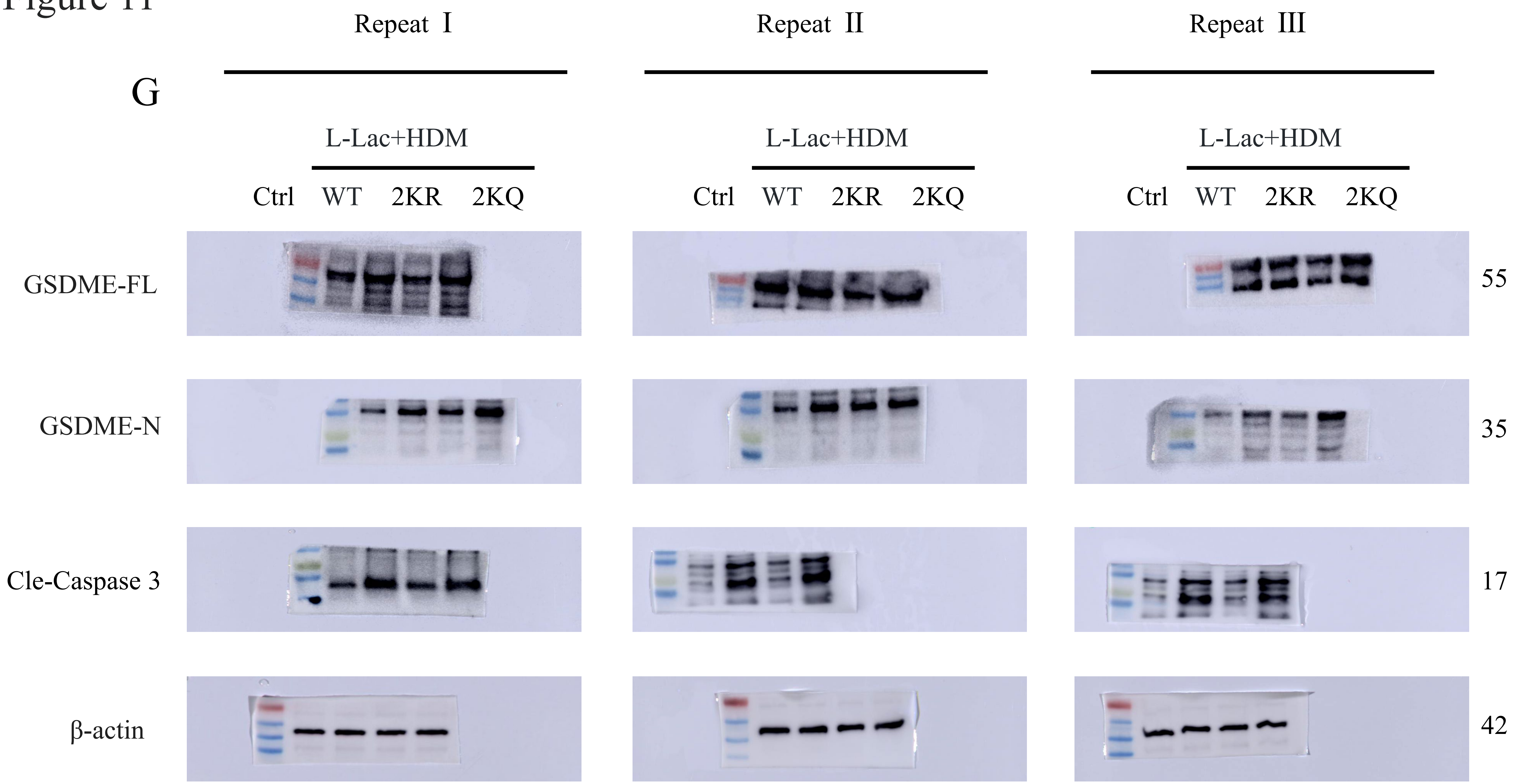
G
Repeat I
Repeat II
Repeat III
L-Lac+HDM
L-Lac+HDM
L-Lac+HDM
Ctrl
WT
2KR
2KQ
Ctrl
WT
2KR
2KQ
Ctrl
WT
2KR
2KQ
GSDME-FL
GSDME-N
Cle-Caspase 3
β-actin
55
35
17
42

Figure 11

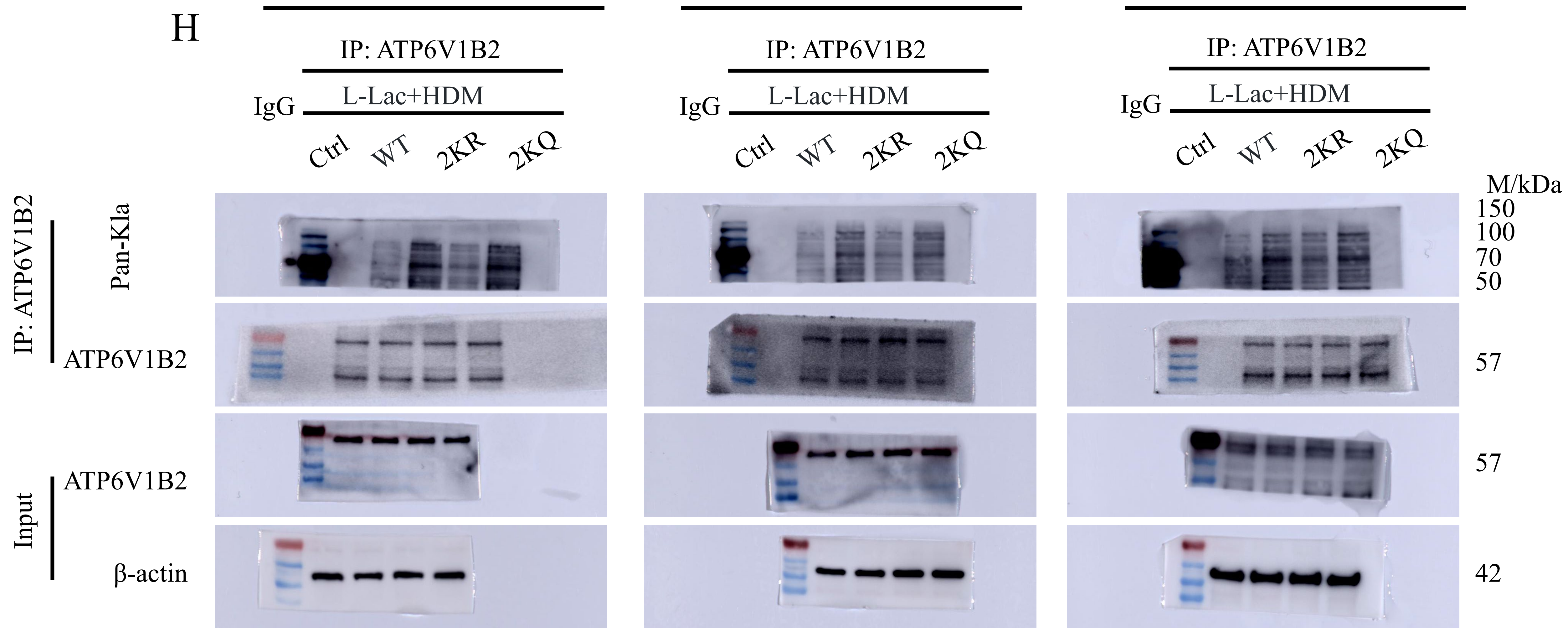
H
Repeat I
Repeat II
Repeat III
IP: ATP6V1B2
IgG
L-Lac+HDM
Ctrl
WT
2KR
2KQ
M/kDa
IP: ATP6V1B2
Pan-Kla
ATP6V1B2
Input
ATP6V1B2
β-actin
150
100
70
50
57
57
42

Figure S1

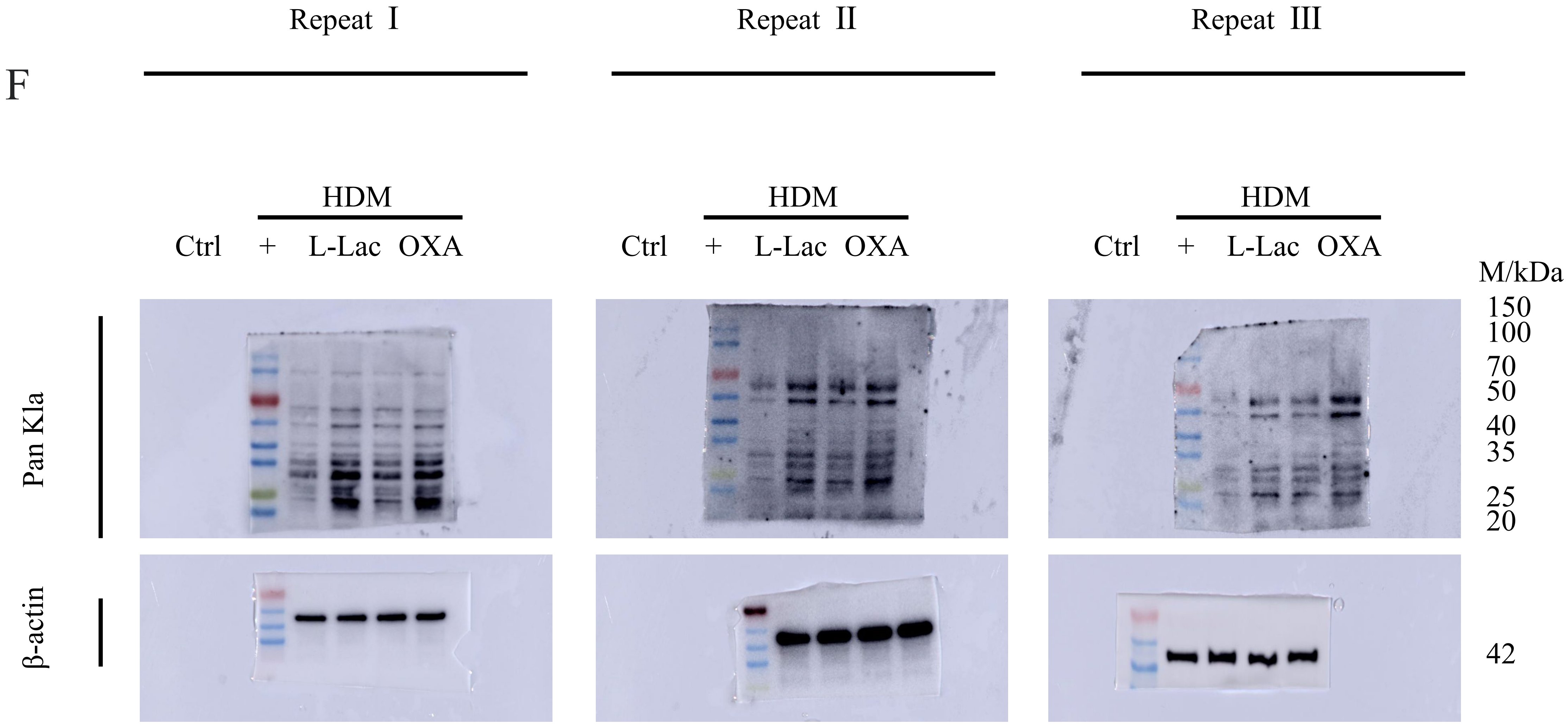
F
Repeat I
Repeat II
Repeat III
HDM
Ctrl
+
L-Lac
OXA
M/kDa
150
100
70
50
40
35
25
20
42
Pan Kla
β-actin

Figure S1

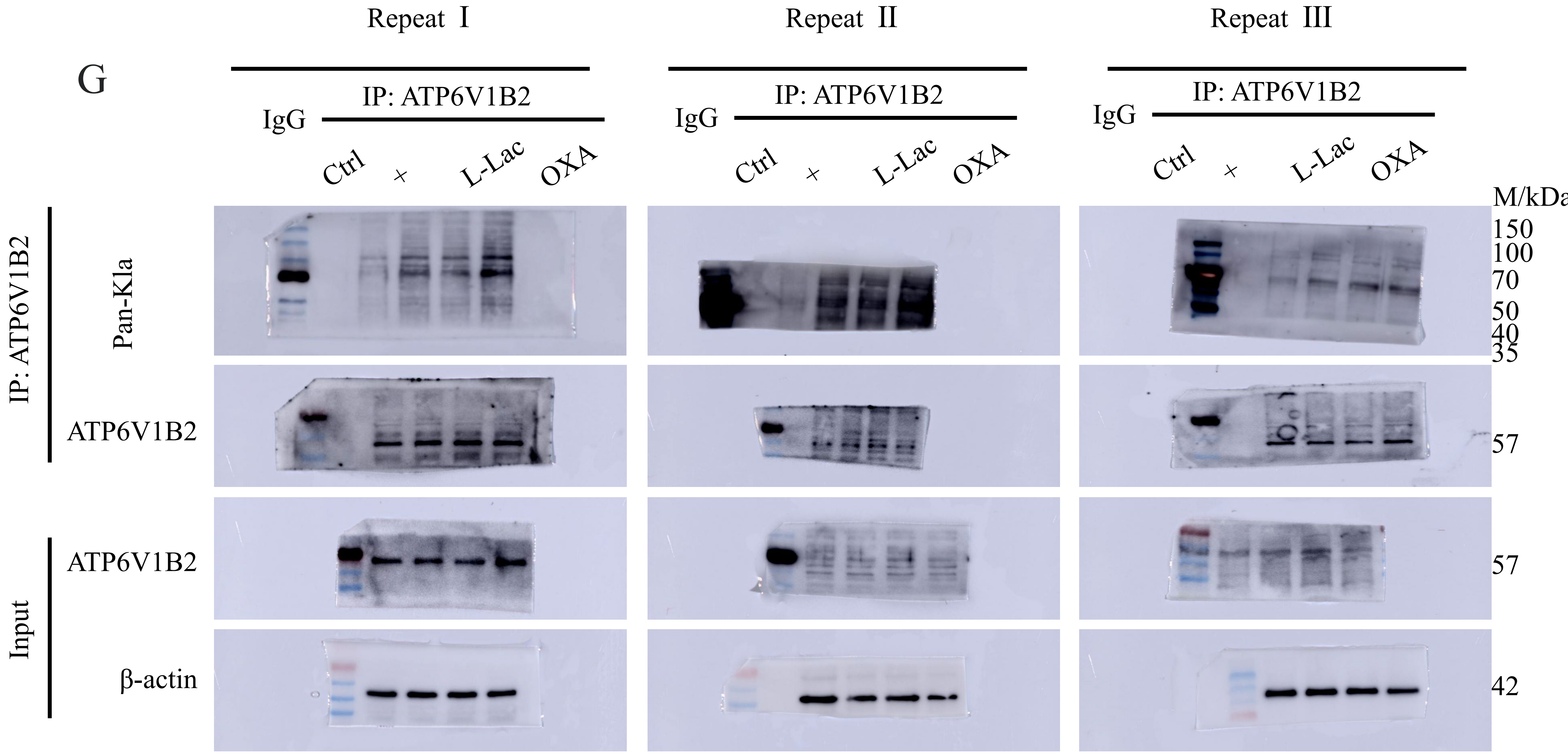

G
Repeat I
Repeat II
Repeat III
IgG
IP: ATP6V1B2
Ctrl
+
L-Lac
OXA
M/kDa
150
100
70
50
40
35
57
57
42
IP: ATP6V1B2
Pan-Kla
ATP6V1B2
Input
ATP6V1B2
β-actin

Figure S2

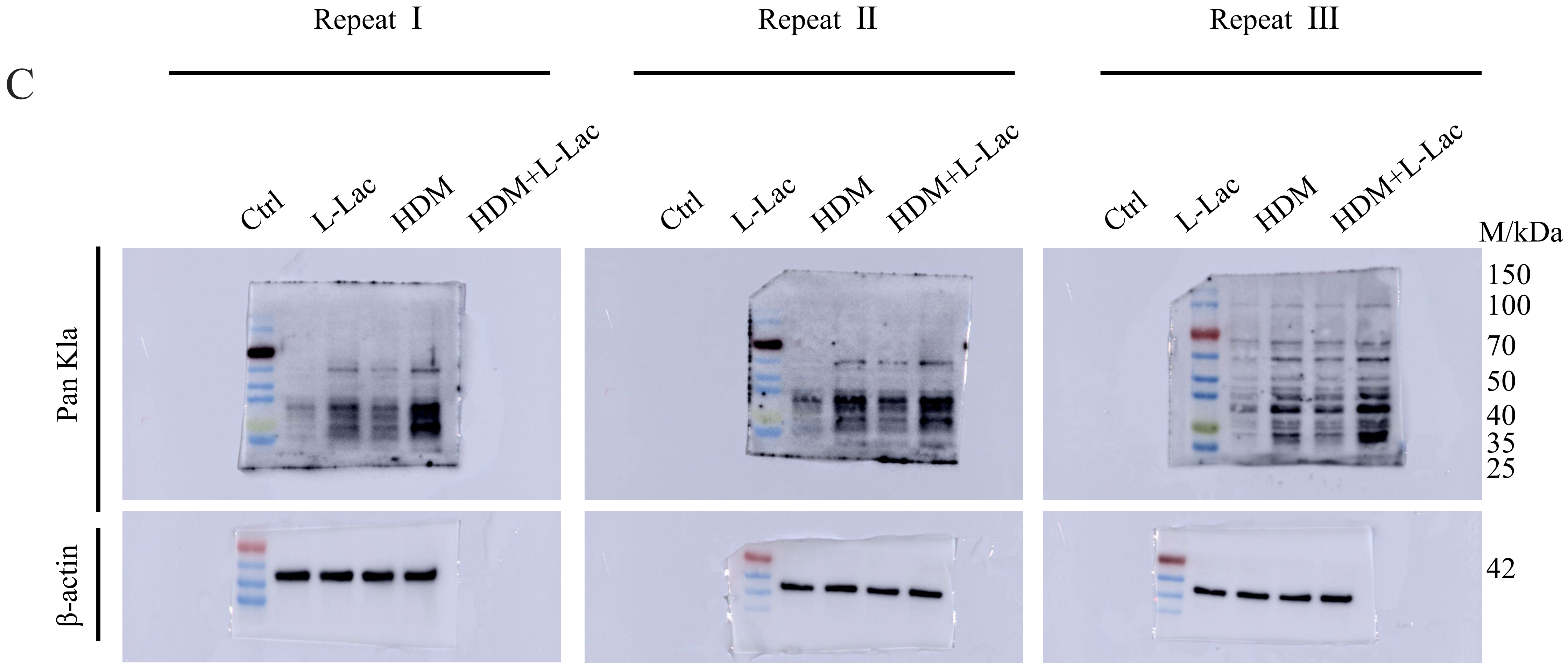

C
Repeat I
Repeat II
Repeat III
Ctrl
L-Lac
HDM
HDM+L-Lac
M/kDa
150
100
70
50
40
35
25
42
Pan Kla
β-actin

Figure S2

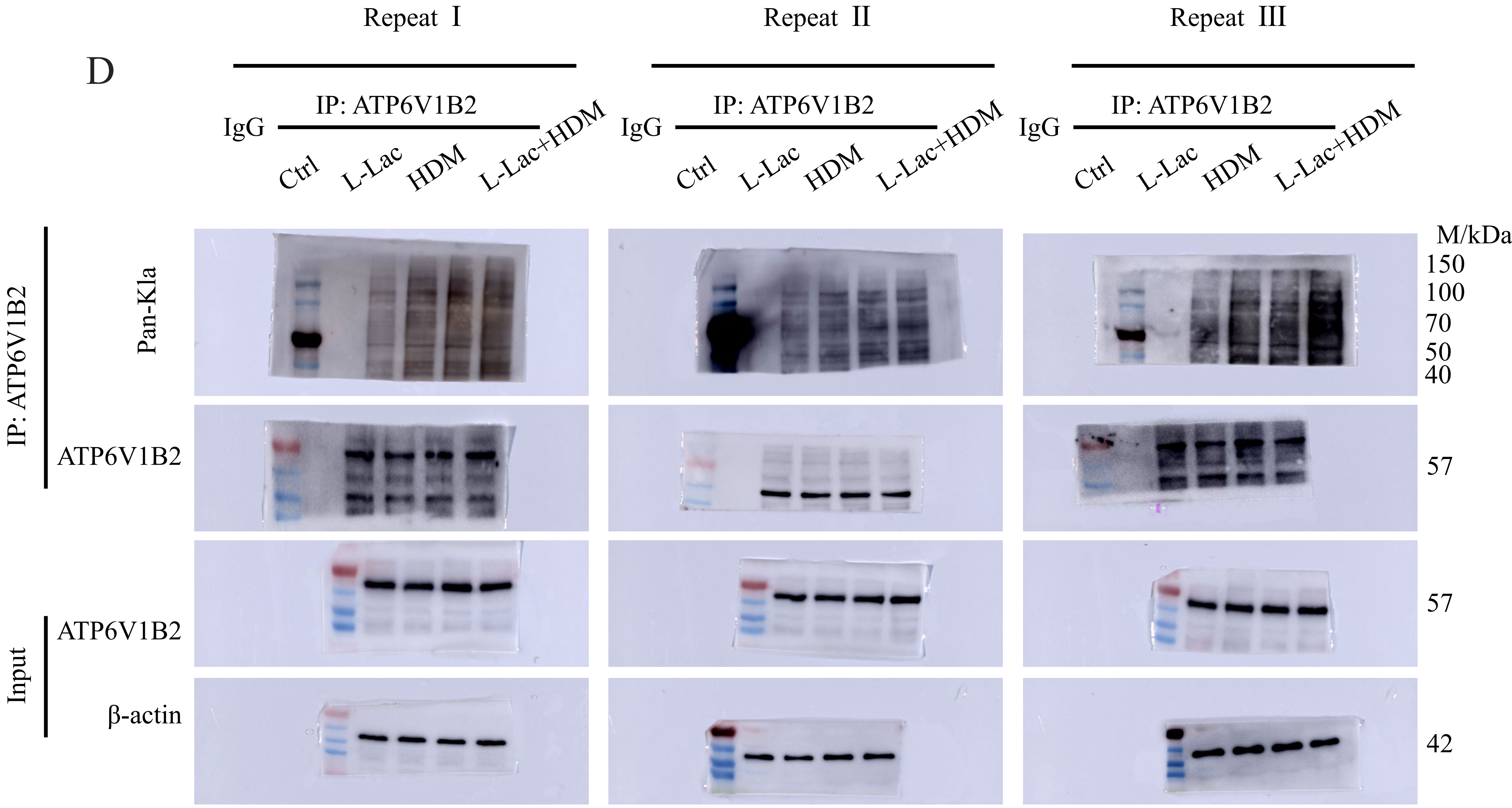

D
Repeat I
Repeat II
Repeat III
IgG
IP: ATP6V1B2
Ctrl
L-Lac
HDM
L-Lac+HDM
IP: ATP6V1B2
Pan-Kla
ATP6V1B2
Input
ATP6V1B2
β-actin
M/kDa
150
100
70
50
40
57
57
42

Figure S3

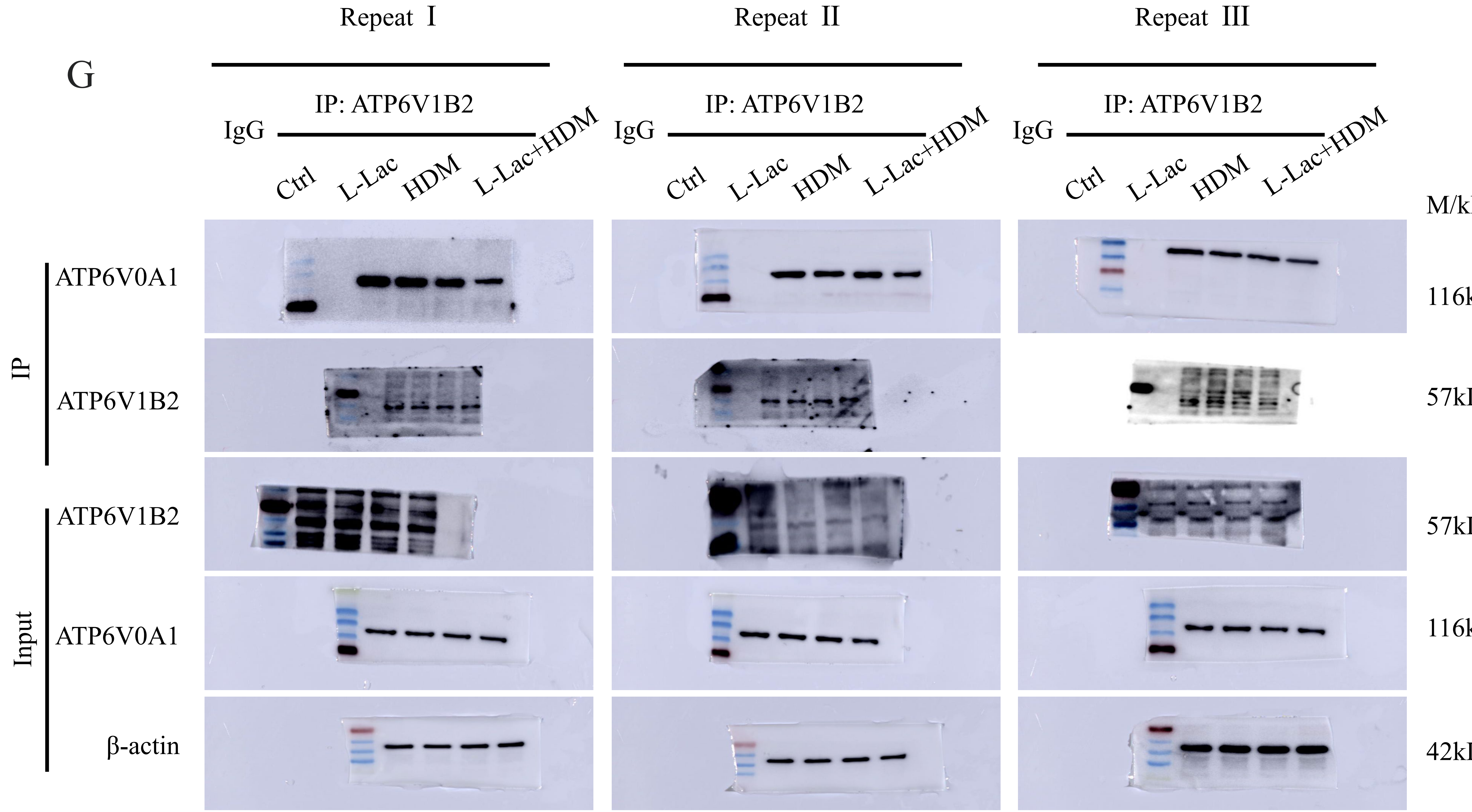
G
Repeat Ⅰ
Repeat Ⅱ
Repeat Ⅲ
IP: ATP6V1B2
IgG
Ctrl
L-Lac
HDM
L-Lac+HDM
IP
Input
ATP6V0A1
ATP6V1B2
β-actin
116kD
57kD
42kD

Figure S4

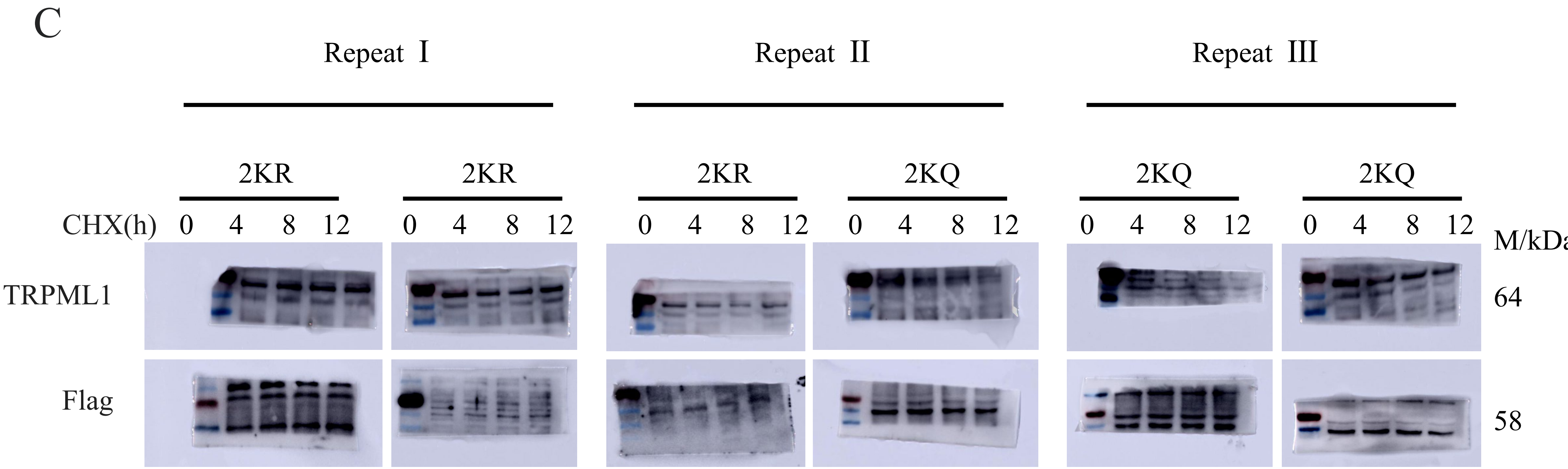
C
Repeat Ⅰ
Repeat Ⅱ
Repeat Ⅲ
2KR
2KR
2KR
2KQ
2KQ
2KQ
CHX(h) 0 4 8 12 0 4 8 12 0 4 8 12 0 4 8 12 0 4 8 12 0 4 8 12
M/kDa
TRPML1
64
Flag
58

Figure S5

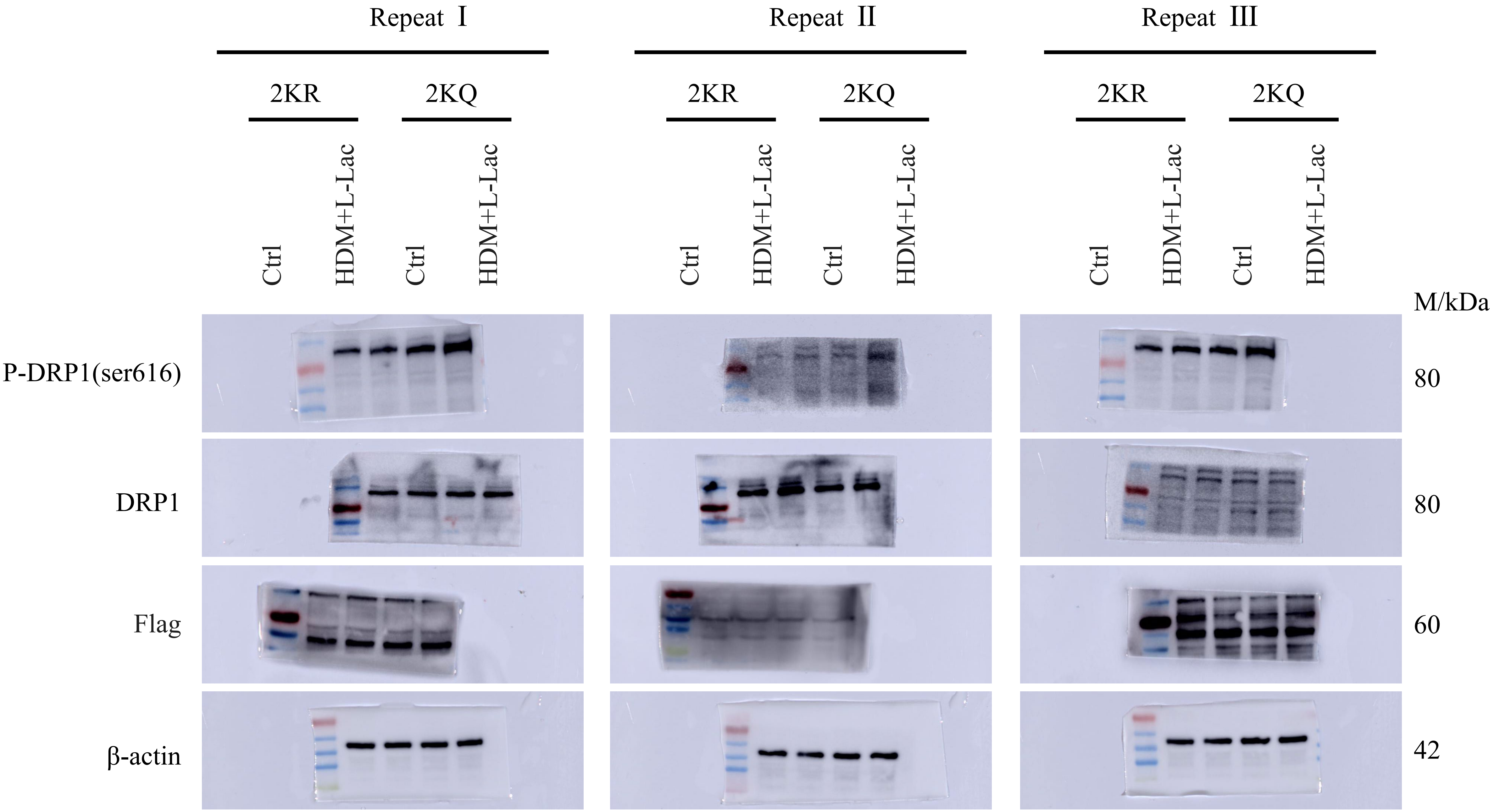
Repeat Ⅰ
Repeat Ⅱ
Repeat Ⅲ
2KR
2KQ
Ctrl
HDM+L-Lac
M/kDa
P-DRP1(ser616)
DRP1
Flag
β-actin
80
80
60
42

Figure S6

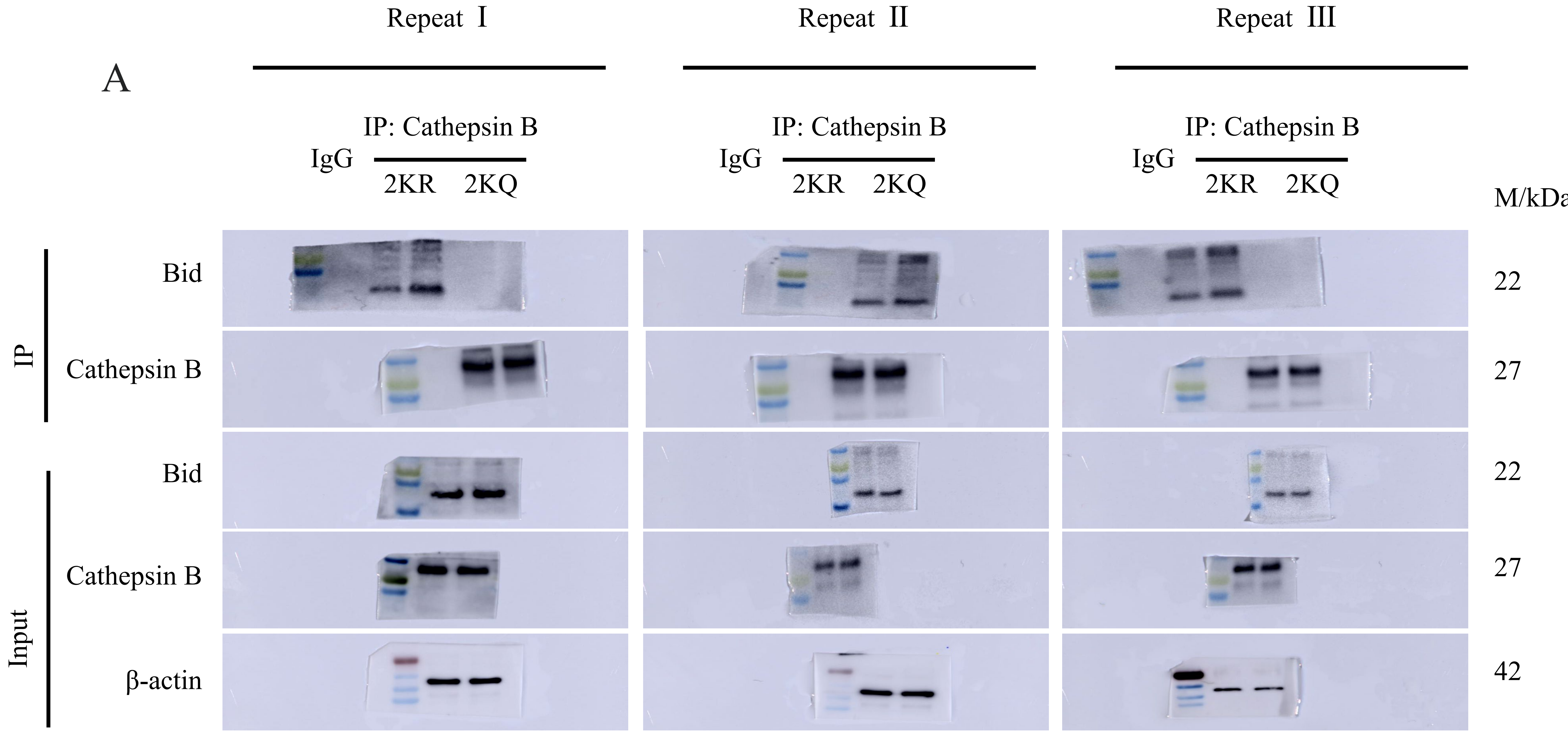
A
Repeat Ⅰ
Repeat Ⅱ
Repeat Ⅲ
IP: Cathepsin B
IgG
2KR
2KQ
M/kDa
IP
Input
Bid
Cathepsin B
Bid
Cathepsin B
β-actin
22
27
22
27
42

Figure S6

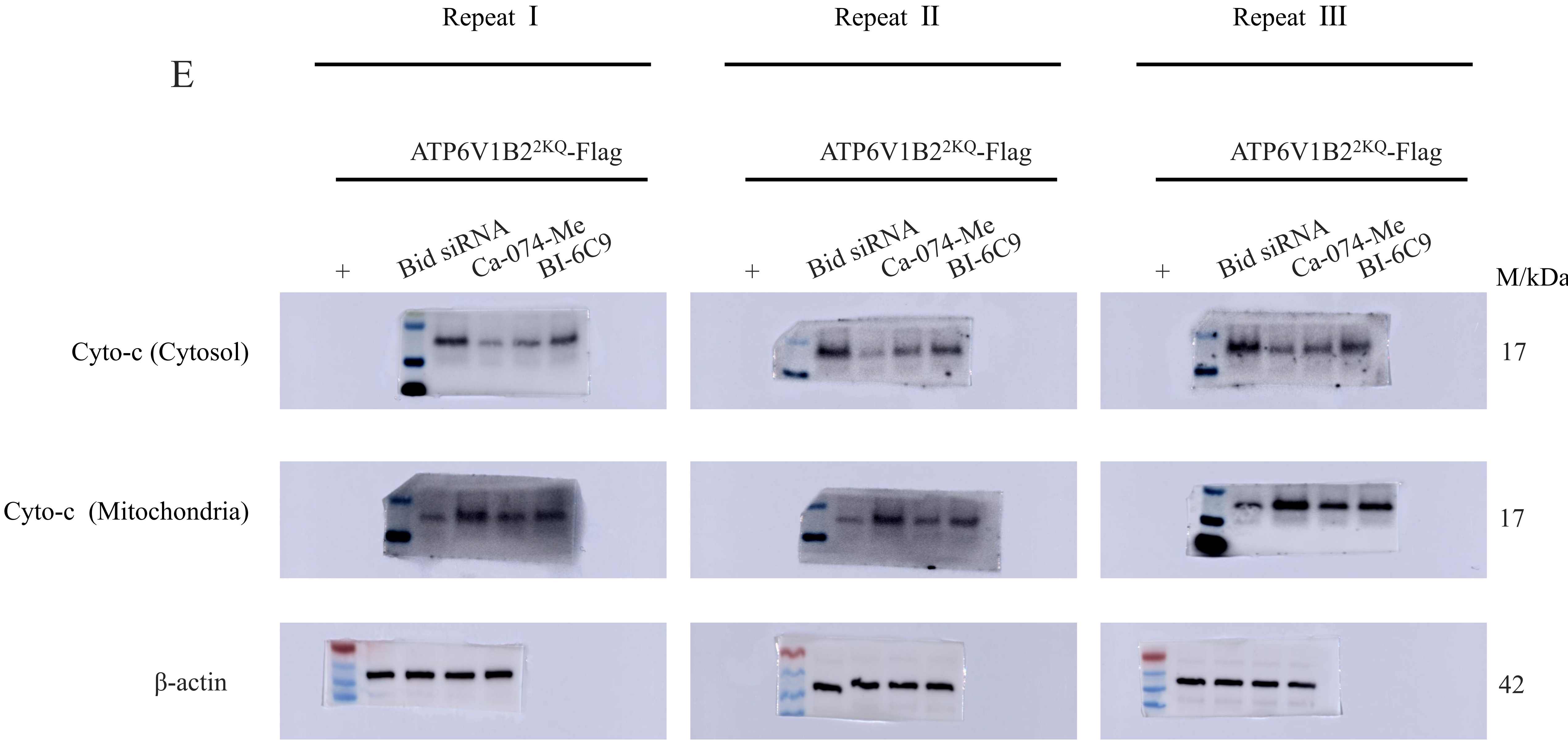
E
Repeat I
Repeat II
Repeat III
ATP6V1B2^2KQ-Flag
ATP6V1B2^2KQ-Flag
ATP6V1B2^2KQ-Flag
+
Bid siRNA
Ca-074-Me
BI-6C9
M/kDa
Cyto-c (Cytosol)
17
Cyto-c (Mitochondria)
17
β-actin
42

Figure S8

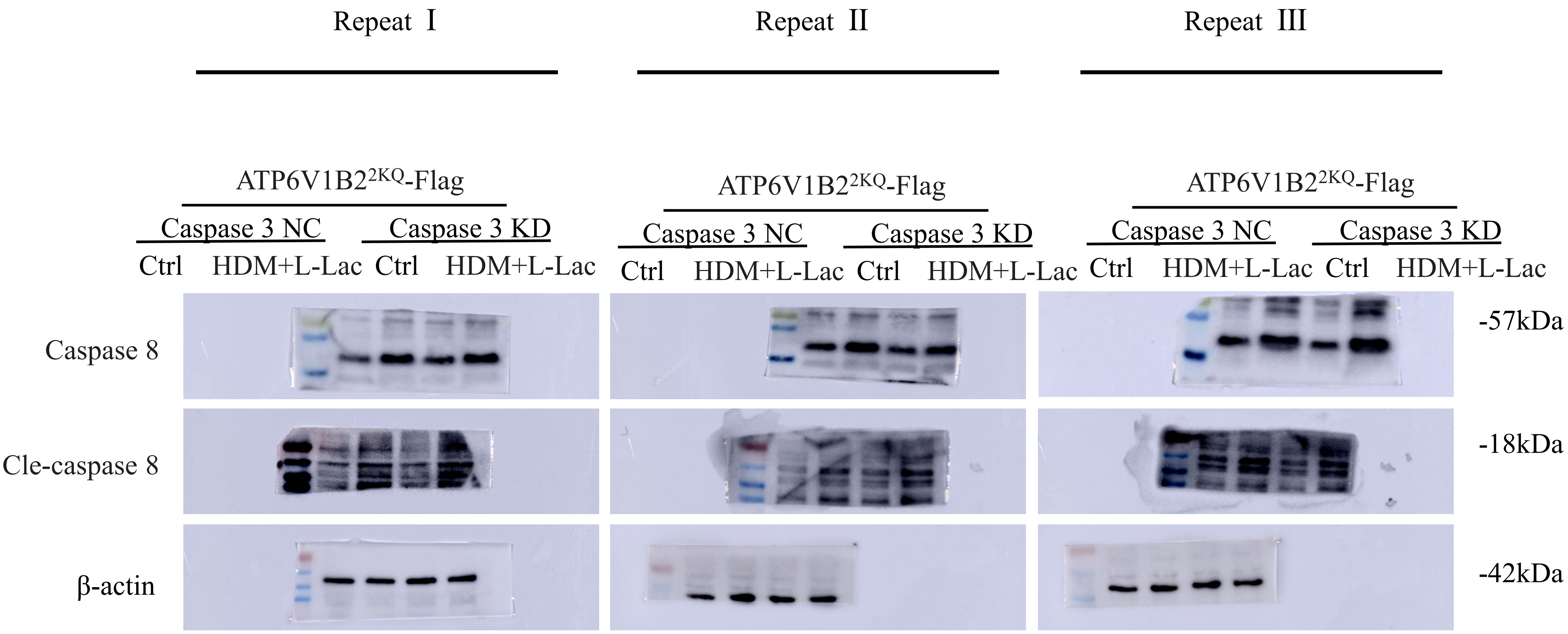
Repeat I
Repeat II
Repeat III
ATP6V1B2^2KQ-Flag
Caspase 3 NC
Caspase 3 KD
Ctrl
HDM+L-Lac
Caspase 8
Cle-caspase 8
β-actin
-57kDa
-18kDa
-42kDa
